# A RsrC-RsrA-RsrB transcriptional circuit positively regulates polysaccharide-degrading enzyme biosynthesis and development in *Penicillium oxalicum*

**DOI:** 10.1038/s42003-024-06536-4

**Published:** 2024-07-11

**Authors:** Yuan-Ni Ning, Xue Liang, Xin Shen, Di Tian, Wen-Tong Li, Xue-Mei Luo, Jia-Xun Feng, Shuai Zhao

**Affiliations:** 1 State Key Laboratory for Conservation and Utilization of Subtropical Agro-bioresources, Guangxi University, 100 Daxue Road, Nanning, Guangxi 530004 P. R. China; 2 Guangxi Research Center for Microbial and Enzyme Engineering Technology, Guangxi University, 100 Daxue Road, Nanning, Guangxi 530004 P. R. China; 3 College of Life Science and Technology, Guangxi University, 100 Daxue Road, Nanning, Guangxi 530004 P. R. China

**Keywords:** Microbiology, Epigenetics

## Abstract

Filamentous fungi produce polysaccharide-degrading enzymes, which is controlled by poorly understood transcriptional circuits. Here we show that a circuit comprising RsrC-RsrA-RsrB (Rsr: production of **r**aw-**s**tarch-degrading enzyme **r**egulator) that positively regulates production of raw starch-degrading enzymes in *Penicillium oxalicum*. Transcription factor (TF) RsrA is essential for biosynthesis of raw starch-degrading enzymes. RsrB and RsrC containing Zn2Cys6- and C2H2-zinc finger domains, act downstream and upstream of RsrA, respectively. RsrA activates *rsrB* transcription, and three nucleotides (G^-286^, G^-287^ and G^-292^) of *rsrB* promoter region are required for RsrA, in terms of TF, for binding. RsrB_165−271_ binds to DNA sequence 5’-TCGATCAGGCACGCC-3’ in the promoter region of the gene encoding key raw-starch-degrading enzyme PoxGA15A. RsrC specifically binds *rsrA* promoter, but not amylase genes, to positively regulate the expression of *rsrA* and the production of raw starch-degrading enzymes. These findings expand complex regulatory network of fungal raw starch-degrading enzyme biosynthesis.

## Abstract (web summary)

A novel circuit comprising RsrC-RsrA-RsrB (Rsr: production of raw-starch-degrading enzyme regulator) was identified to positively regulate the production of raw starch-degrading enzymes in Penicillium oxalicum.

## Introduction

Amylase is an important industrial enzyme^1^, which can convert starch into glucose and/or maltose^2,3^. Traditional starch processing comprising of gelatinisation, liquefaction and saccharification, involves soluble starch-degrading enzyme, with all steps requiring high energy input^4^. In general, soluble starch-degrading enzymes are recognized as glycoside hydrolases degrading soluble or cooked starch. By contrast, raw starch-degrading enzymes are capable of directly degrading raw or uncooked starch granules into oligosaccharides or glucose below gelatinisation temperature^5^. Application of raw starch-degrading enzymes can improve production efficiency and reduce input costs and environmental pollution, giving them great market application prospects^6^.

*Penicillium* can secrete intact and highly active raw starch-degrading enzyme^7^, including raw starch-degrading glucoamylase PoxGA15A^8^. However, low production of native raw starch-degrading enzymes means they cannot meet the needs of large-scale industrial applications, hence it is urgent to elucidate the regulatory mechanisms of amylase gene expression to guide molecular breeding of fungal strains that produce large quantities of raw starch-degrading enzymes.

Expression of amylase genes in *Penicillium oxalicum* is controlled by a variety of regulatory factors. For instance, activator AmyR binds to the promoters of genes encoding prominent amylases including PoxGA15A and α-amylase Amy13A. AmyR interacts with histone acetyltransferase complex Hat1-Hat2, acting as a molecular brake to regulate expression of amylase genes^9^. Moreover, CxrC negatively regulates the expression of genes encoding major raw starch-degrading enzymes^10^. Other specific regulatory factors such as translational elongation factor eEF1A^11^, G protein γ subunit GNG-1^12^, protein kinases PoxMK1^13^, PoxMKK1^14^ and POGSK‑3β^15^, GATA-type zinc finger protein NsdD^16^ and putative methyltransferase Mtr23B^17^, are known to participate in raw starch-degrading enzyme biosynthesis in *P*. *oxalicum*.

Previous studies identified a transcription factor (TF), RsrA (formerly POX01907), containing two SANT-like domains with different roles^18^. SANT1 is responsible for DNA-binding, while SANT2 interacts with a putative 3-hydroxyisobutyryl-CoA hydrolase. RsrA positively regulates the expression of *PoxGA15A* and *amy13A*, and this regulation is dependent on its phosphorylation and the recruitment of Mediator complex^19^. However, the regulatory circuit associated with RsrA remains unknown.

In present study, TFs RsrB and RsrC acting downstream and upstream of RsrA, respectively, were identified in *P*. *oxalicum* through comparative transcriptomic profiling and yeast one-hybridisation (Y1H). Both of them positively regulated the biosynthesis of raw starch-degrading enzymes and conidiation, constructing a regulatory circuit RsrC-RsrA-RsrB.

## Results

### POX_g08691 positively regulates amylase production of *P*. *oxalicum*

Comparative analysis of transcriptomes from *P*. *oxalicum* mutant Δ*rsrA* and parental strain Δ*ku70* was performed following culture in medium containing commercial starch of corn for 4 h, and 15 differentially expressed genes (DEGs) encoding putative TFs were identified in Δ*rsrA* relative to Δ*ku70*, using thresholds of |Log2 fold change| >1.5 and *p* value < 0.05^18^. These thresholds were selected with aim to increase the probability of screening target genes. Of these, six DEGs were successfully deleted in Δ*ku70* in previous reports^20^. Herein, deletion mutants of the remaining nine DEGs were constructed (Supplementary Fig. S1). Further measurement of raw starch-degrading enzyme activity revealed seven mutants with significantly altered enzyme production relative to Δ*ku70*. Notably, mutant Δ*POX_g08691* had the greatest reduction in enzyme production (49.5%) when directly cultured on commercial starch of corn for 6 days (Supplementary Fig. S2). Therefore, POX_g08691 was selected for further analysis.

**Fig. 1:**
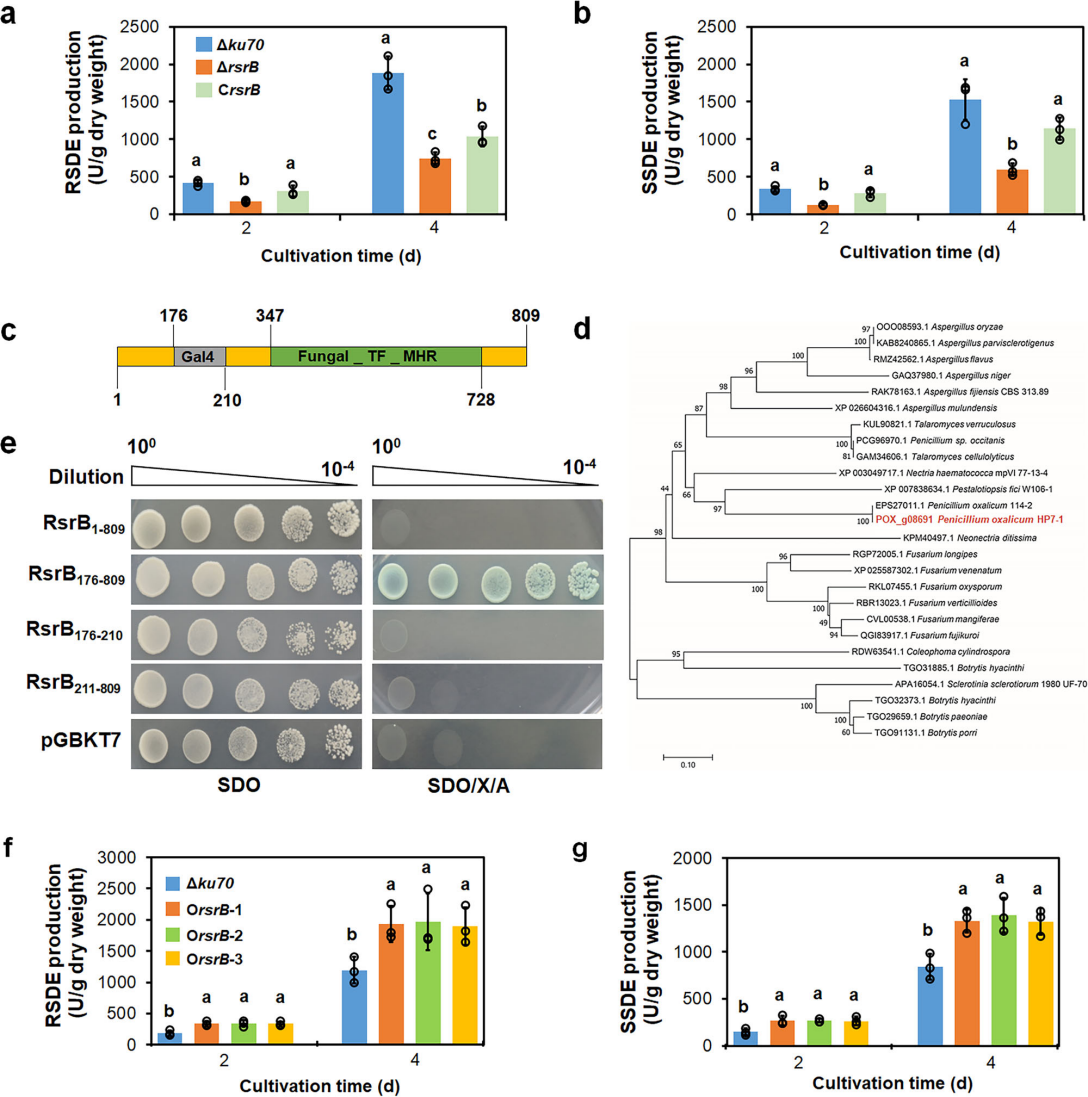
Analysis of RsrB (POX_g08691) functions and protein sequences. **a** Production of raw starch-degrading enzymes (RSDEs) and soluble starch-degrading enzymes (SSDEs) (**b**) by *P*. *oxalicum* mutant Δ*rsrB*, parental strain Δ*ku70* and complementation strain C*rsrB* in the presence of soluble corn starch (SCS). Lowercase letters represent *p* < 0.05. Different letters indicate significant differences, evaluated by one-way ANOVA. **c** Conserved domains in RsrB. Grey and green areas indicate the Gal4-like Zn2Cys6 zinc finger domain (Gal4) and Fungal_TF_MHR domain, respectively. **d** Phylogenetic analysis of RsrB and its homologues. The cladogram was constructed by MEGA version X using the neighbour-joining method and a Poisson model. Values displayed on branches were calculated using 1000 bootstrap replicates. **e** Yeast self-activation assay. Yeast cells carrying different lengths of RsrB peptides were cultured on SDO (SD/-Trp) and SDO/X/A (SD-Trp/+ X-α-Gal /+ aureobasidin A) for 4 days. **f** Effects of *rsrB* overexpression on RSDE and SSDE (**g**) production of *P. oxalicum*. All tested *P. oxalicum* strains were cultured in medium containing soluble corn starch for 2–4 days after transfer from glucose. Each mutant included three independent transformants. Results are mean ± SD. All experiments were performed at least three times. Uppercase and lowercase letters represent *p* < 0.01 and *p* < 0.05, respectively. Different letters indicate significant differences, evaluated by one-way ANOVA.

Moreover, complementation strain C*POX_g08691* was constructed and confirmed (Supplementary Fig. S3), where the complementation cassette of *POX_g08691* was introduced into an ectopic locus of *POX_d05452* encoding aspartic protease PepA. Deletion of *POX_d05452* did not affect the production of amylases by *P*. *oxalicum*^21^. Subsequently, it was found that production of raw starch-degrading enzymes and soluble starch-degrading enzymes by Δ*POX_g08691* was 58.4–63.0% lower than that by Δ*ku70* when cultivated for 2 or 4 days. Complementation strain C*POX_g08691* partially restored amylase production compared with Δ*ku70* (Fig. 1a, b). In addition, sodium dodecyl sulphate-polyacrylamide gel electrophoresis (SDS-PAGE) analysis indicated that the secreted extracellular proteins by Δ*POX_g08691* were lower than that by Δ*ku70* when cultivated for 4 days. The extracellular proteins of C*POX_g08691* were comparable to those of Δ*ku70* (Supplementary Fig. S3c). These results indicate that POX_g08691 positively regulates the biosynthesis of amylases in *P. oxalicum*.

### RsrB is a TF containing a Zn2Cys6 zinc finger domain

Genome annotation of *P*. *oxalicum* HP7-1^22^ indicated that POX_g08691 consisting of 809 amino acid residues, contained a Gal4-like Zn2Cys6 zinc finger domain and a Fungal_trans domain (Fig. 1c). POX_g08691 shares 98.9% identity with PDE_01952 (EPS27011.1) from *P. oxalicum* 114-2, but <50% identity with orthologs in other fungal strains. Evolutionary analysis indicated that POX_g08691 and its orthologs were similar in *Penicillium* and *Aspergillus* (Fig. 1d). To facilitate further study, POX_g08691 was re-designated RsrB (Production of **r**aw-**s**tarch-degrading enzyme **r**egulator **B**).

To determine whether RsrB has transcriptional activation ability, an autoactivation experiment was conducted in yeast Y2HGold cells. The full-length *rsrB* gene and a DNA fragment encoding polypeptides RsrB_176–809_, RsrB_176–210_ and RsrB_211–809_ were separately cloned into plasmid pGBKT7, and the resulting recombinant plasmids were introduced into yeast Y2HGold cells. Recombinant Y2HGold/pGBKT7-*rsrB*_1–809_, Y2HGold/pGBKT7-*rsrB*_176–210_ and Y2HGold/pGBKT7-*rsrB*_211–809_ grew normally on SDO (SD/-Trp) plates but not on SDO/X/A (SD/-Trp/+X-α-Gal/+aureobasidin A). By comparison, recombinant Y2HGold/pGBKT7-*rsrB*_176–809_ grew normally on both SDO and SDO/X/A plates. In addition, colonies of Y2HGold/pGBKT7-*rsrB*_176–809_ turned blue on SDO/X/A plates (Fig. 1e), indicating that RsrB_176–809_ has transcriptional activation activity.

### RsrB affects mycelial growth and conidiation of *P*. *oxalicum*

To explore the effects of RsrB on colony growth and sporulation, Δ*rsrB* was cultivated on solid plates containing PDA, glucose and commercial starch of corn, and the parental strain Δ*ku70* and complementation strain C*rsrB* served as controls. When cultured for 5 days, the diameter of ∆*rsrB* colonies was smaller than that of Δ*ku70* and C*rsrB* on PDA, but larger than on glucose and commercial starch of corn, respectively (Supplementary Fig. S4a). Quantitative analysis of asexual spores revealed that mutant Δ*rsrB* production of asexual spores was increased by 63.0%, 49.0% and 49.0% compared with Δ*ku70* on PDA, glucose, and commercial starch of corn, respectively (Supplementary Fig. S4b).

Investigation of mycelial growth affected by RsrB displayed that mycelial accumulation in Δ*rsrB* was enhanced by 17.2–43.2% and 17.2–28.8% in glucose and commercial starch of corn, respectively (Supplementary Fig. S4c, d). Under microscopy, the hyphae of Δ*rsrB* cultivated for 48 h produced conidiospores earlier than those of both Δ*ku70* and C*rsrB* after cultivation on PDA, glucose and commercial starch of corn, whereas C*rsrB* conidiospores were similar to those of Δ*ku70* (Supplementary Fig. S5).

### Overexpression of *rsrB* markedly improves amylase production

Moreover, overexpression strain O*rsrB* was constructed (Supplementary Fig. S6). In O*rsrB*, the overexpression cassette of *rsrB* was introduced into an ectopic locus of *POX_d05452*^21^ in Δ*ku70*, where *rsrB* is controlled by its own promoter. Further RT-qPCR assay revealed that the transcriptional level of *rsrB* was considerably enhanced by 0.69–1.87-fold in O*rsrB* relative to Δ*ku70* in the presence of commercial starch of corn for 12–48 h (Supplementary Fig. S6c). The production of raw starch-degrading enzymes and soluble starch-degrading enzymes by O*rsrB* was enhanced by 61.4–81.2% after induction of commercial starch of corn for 2–4 days (Fig. 1f, g). SDS-PAGE analysis displayed that the secreted extracellular proteins by O*rsrB* was enhanced than that by Δ*ku70* when cultivated for 2 or 4 days (Supplementary Fig. S6d). However, colony phenotypes and sporulation of O*rsrB* on plates containing PDA, glucose and commercial starch of corn were similar to those of Δ*ku70* (Supplementary Fig. S7).

### RsrB regulates expression of genes encoding amylase and sporulation

To elucidate the effects of RsrB on expression of genes related to amylase production, RNA-sequencing (RNA-seq) was employed. Total RNAs of both ∆*ku70* and Δ*rsrB* were collected following cultivation in commercial starch of corn medium for 24 h after pre-growth in glucose. Statistical analysis of sequencing data from three biological replicates yielded a high Pearson correlation coefficient (Supplementary Fig. S8a), suggesting that these transcriptomic data were suitable for subsequent analysis.

**Fig. 2:**
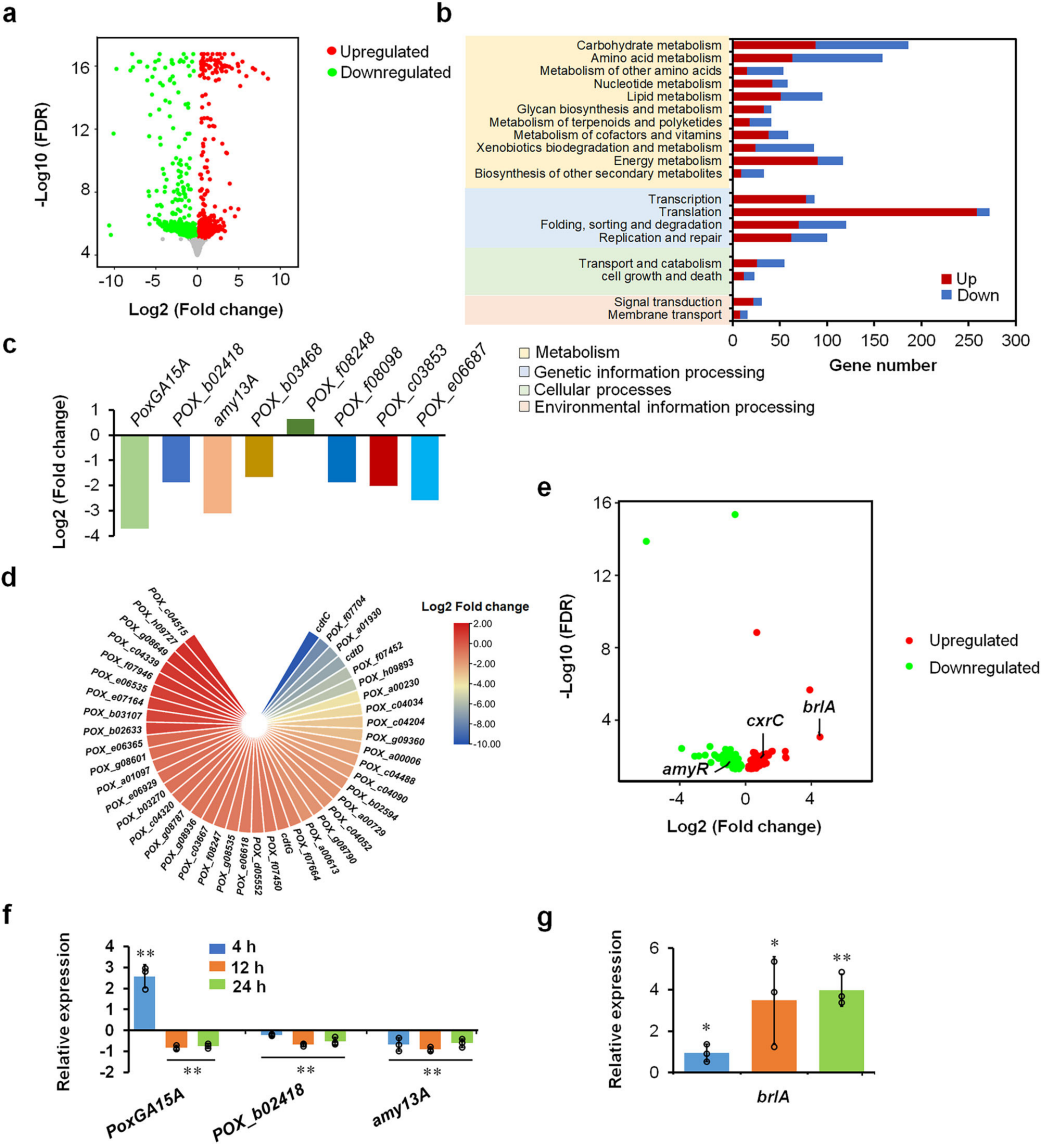
Effects of RsrB on gene expression in *P. oxalicum* in the presence of SCS. **a** Volcano plot indicating differentially expressed genes (DEGs). DEGs were screened and detected with thresholds of 0 <False Discovery Rate (FDR) ≤ 0.05. **b** KEGG annotation of DEGs modulated by RsrB. **c** DEGs encoding amylases. **d** DEGs encoding putative sugar transporters. **e** DEGs encoding putative transcription factors. **f** Real-time reverse transcription quantitative PCR (RT-qPCR) analysis of genes encoding major amylases, as well as sporulation-regulatory gene *brlA* (**g**). All *P*. *oxalicum* strains were cultured for 4–24 h in the presence of SCS. Gene expression in Δ*rsrB* was normalised to the level of Δ*ku70*. Results are mean ± SD. **p < 0.01 and *p < 0.05 indicate significant differences between Δ*rsrB* and Δ*ku70*, calculated by Student’s *t* test. PoxGA15A, raw starch-degrading glucoamylase; POX_b02418, glucoamylase; Amy13A, α-amylase.

The clean reads obtained by RNA-seq were mapped onto the genome of *P*. *oxalicum* strain HP7-1^22^, and the expressed genes were screening. Comparative analysis identified 4041 DEGs in Δ*rsrB* compared with ∆*ku70*, with a threshold False Discovery Rate (FDR) < 0.05, consisting of 2265 upregulated (0.08 <log2 fold change <8.5) and 1776 downregulated (-10.6 <log2 fold change <-0.08) genes (Fig. 2a and Supplementary Data 1). These DEGs were mainly involved in metabolism (651 genes) and genetic information processing (521 genes), especially carbohydrate metabolism (186 genes), and translation (272 genes; Fig. 2b).

Among the 4041 DEGs, eight genes encoding amylolytic enzymes were identified, including key raw starch-degrading glucoamylase gene *PoxGA15A*, glucoamylase gene *POX_b02418* and α-amylase gene *amy13A*, the expression of which was downregulated (-3.71 <log2 fold change <-1.88) in Δ*rsrB* compared with ∆*ku70* (Fig. 2c). Forty-three DEGs encoding predicted sugar transporters were identified, of which nine were upregulated (0.63 <log2 fold change <1.31) and 34 were downregulated (-9.75 <log2 fold change <-0.44) in Δ*rsrB*. Three key cellulodextrin transporter genes *cdtC*, *cdtD* and *cdtG* were included, with downregulated transcription (Fig. 2d).

Moreover, 184 DEGs encoding putative TFs were identified, 84 of which were upregulated (0.18 <log2 fold change <4.53) and 100 were downregulated (-6.04 <log2 fold change <-0.26) in Δ*rsrB*. Of them, several known TF-encoding genes regulating the biosynthesis of RSDEs in *P*. *oxalicum* were found, such as *cxrC*/*POX_a00541*^10^ and *amyR*/*POX_f08097*^9^, with 84.1%-increased and 47.0%-reduced expression in Δ*rsrB*. The transcription of the key sporulation-regulated *POX_c03558*/*brlA* gene increased 22.6-fold in Δ*rsrB* (Fig. 2e).

Two genes (*POX_a00279*/*rodA-like* and *POX_c03497/rodB*) that are involved in fungal mycelial growth and sporulation^16^ were markedly upregulated by 31.57- and 35.01-fold, respectively, following *rsrB* deletion.

Real-time quantitative reverse transcription PCR (RT-qPCR) assay found that expression of the *PoxGA15A*, *POX_b02418*, *amy13A* and *brlA*, varied in Δ*rsrB* compared with Δ*ku70*. After culturing for 4 h, transcription of *PoxGA15A* in Δ*rsrB* was increased 2.57-fold, whereas *POX_b02418* and *amy13A* were downregulated by 20.8% and 67.5%, respectively. At both 12 and 24 h, transcription of *PoxGA15A*, *POX_b02418* and *amy13A* genes was downregulated 51.3–90.6% (Fig. 2f). By comparison, at both 4, 12 and 24 h, transcription of *brlA* increased 0.94–3.98-fold (Fig. 2g).

### RsrB binds to the promoter regions of amylase genes and *brlA*

**Fig. 3:**
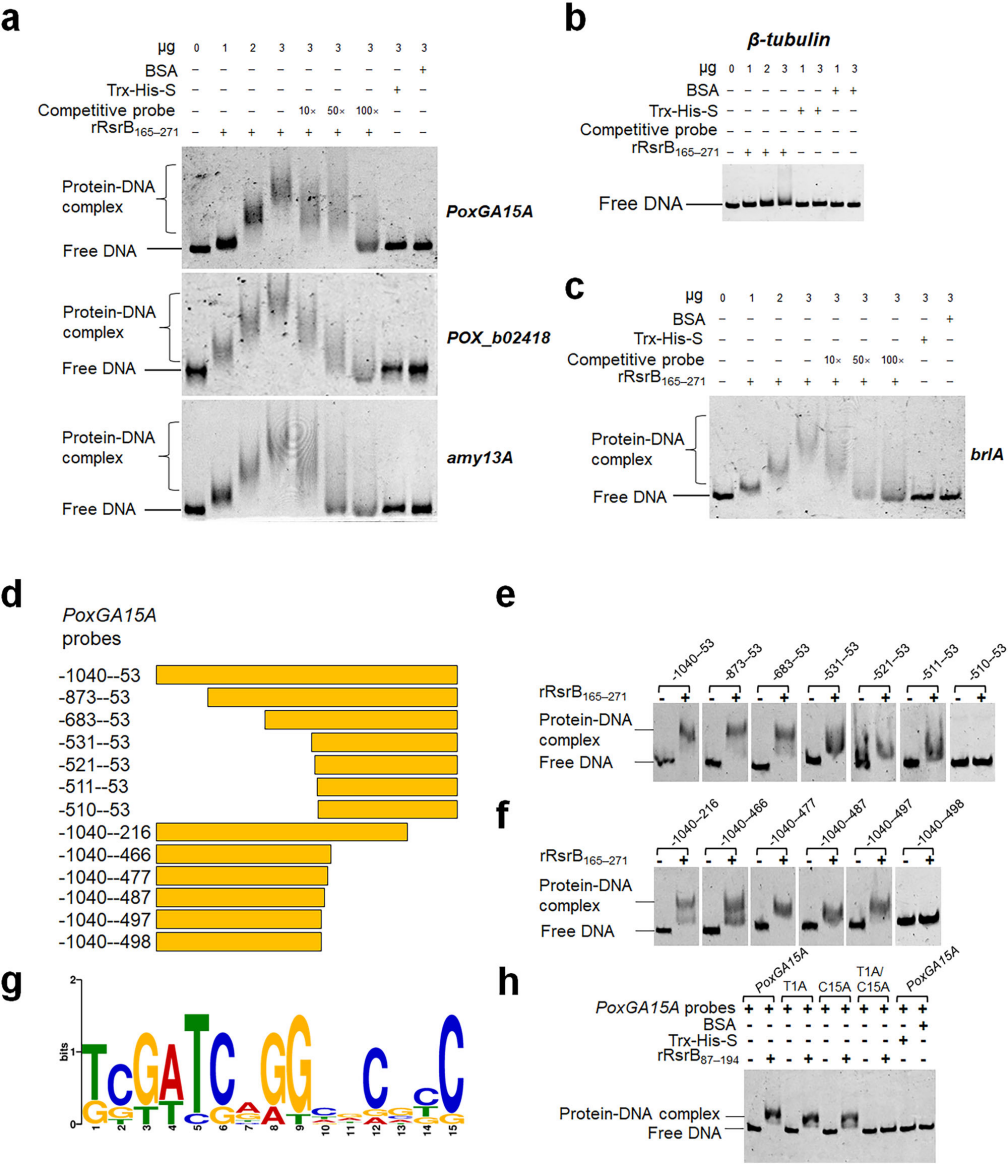
In vitro electrophoretic mobility shift assay (EMSA) indicating RsrB binding to target genes, and identification of the key DNA sequence bound. **a**–**c** Each reaction contained Trx-His-S-tagged rRsrB_165–271_ (0–3 μg) and 6-carboxyfluorescein-tagged probe (~50 ng). Trx-His-S peptide and bovine serum albumen (BSA) served as controls, along with the promoter region of the *β-tubulin* gene. Competitive probes were DNA fragments without 6-carboxyfluorescein. *PoxGA15A*, raw starch-degrading glucoamylase gene; *POX_b02418*, glucoamylase gene; *amy13A*, α-amylase gene; *brlA*, conidiation regulatory gene. **d** Schematic diagram indicating truncated promoter region of *PoxGA15A* probes for in vitro EMSA. **e**, **f** In vitro EMSA between rRsrB_165–271_ and truncated probes of *PoxGA15A*. Each reaction contained Trx-His-S-tagged rRsrB_165–271_ (3 μg) and 6-carboxyfluorescein-tagged probe (~50 ng). **g** MEME-predicted conserved DNA sequence bound by rRsrB_165–271_. DNA sequences were from promoter regions of *PoxGA15A*, *POX_b02418* and *amy13A*. **h** EMSA indicating the binding of mutated *PoxGA15A* probes by rRsrB_165–271_ (3 μg). Mutated *PoxGA15A* probe has ‘A’ instead of T and C at the 1st and 15th positions, as shown in panel D.

In vitro electrophoretic mobility shift assay (EMSA) was performed to determine whether RsrB binds to the promoter regions of amylase genes *PoxGA15A*, *POX_b02418* and *amy13A*. The results showed shifts in bands representing DNA-protein complexes when rRsrB_165–271_ was added to the probe tagged with 6-carboxyfluorescein (6-FAM), and the band density increased with an increasing amount of rRsrB_165–271_. As expected, neither control probe *β-tubulin* gene promoter nor proteins BSA and Trx-His-S formed DNA-protein complexes. Subsequent competitive EMSA indicated that the formed complexes markedly decreased or disappeared with increased competitive probes lacking 6-FAM (Fig. 3a, b). Similarly, a shifted band appeared when the mixture of rRsrB_165–271_ and *brlA* probe was loaded, but not when loading the competitive probe lacking 6-FAM (Fig. 3c). These results suggest that RsrB specifically binds to the promoter regions of the tested amylase genes and *brlA*.

### RsrB binds to core DNA sequence 5’-KBKWYSNRKNDVVBS-3’

To identify DNA sequences bound by rRsrB_165–271_, a set of truncated *PoxGA15A* probes was designed, used for in vitro EMSA. The *PoxGA15A*_-1040–-53_ probe was successively truncated when attached to the 3’-terminus, generating several DNA fragments (Fig. 3d). All truncated probes tagged with 6-FAM formed DNA-protein complexes with recombinant rRsrB_165–271_ except *PoxGA15A*_-510–-53_ (Fig. 3e). Similarly, when attached the 5’-terminus, the *PoxGA15A*_-1040–-53_ probe was truncated to different lengths of DNA fragments (Fig. 3d). In vitro EMSA showed that rRsrB_165–271_ could bind to all tested probes except *PoxGA15A*_-1040–-498_ (Fig. 3f). It therefore appears that the core-DNA sequence bound by rRsrB_165-271_ is *PoxGA15A*_-511–-497_ (5’-TCGATCAGGCACGCC-3’).

Analysis by the MEME suite (https://meme-suite.org/meme/) with promoter regions from *PoxGA15A*, *POX_b02418* and *amy13A* identified the core DNA-binding sequence 5’-KBKWYSNRKNDVVBS-3’ (K: G/T; B: G/C/T; W: A/T; Y: C/T; S: G/C; N: G/A/C/T; R: A/G; D: G/A/T; V: G/A/C) shown in Fig. 3g. Based on the above results (Fig. 3e and f), two key nucleotides (T1 and C15) were localised at the binding site of rRsrB_165-271_ at the *PoxGA15A* promoter. When they were separately mutated to A, the shifted bands formed by rRsrB_165-271_ and the mutated probes *PoxGA15A*^T1A^ or *PoxGA15A*^C15A^ became weaker. When they were both mutated to A, the double mutated probe *PoxGA15A*^T1A/C15A^ cannot be bound by rRsrB_165-271_ (Fig. 3h), suggesting that these nucleotides were essential for the target sequence to be bound by rRsrB_165-271_.

### RsrB acts downstream of RsrA but with specific roles in regulation

**Fig. 4:**
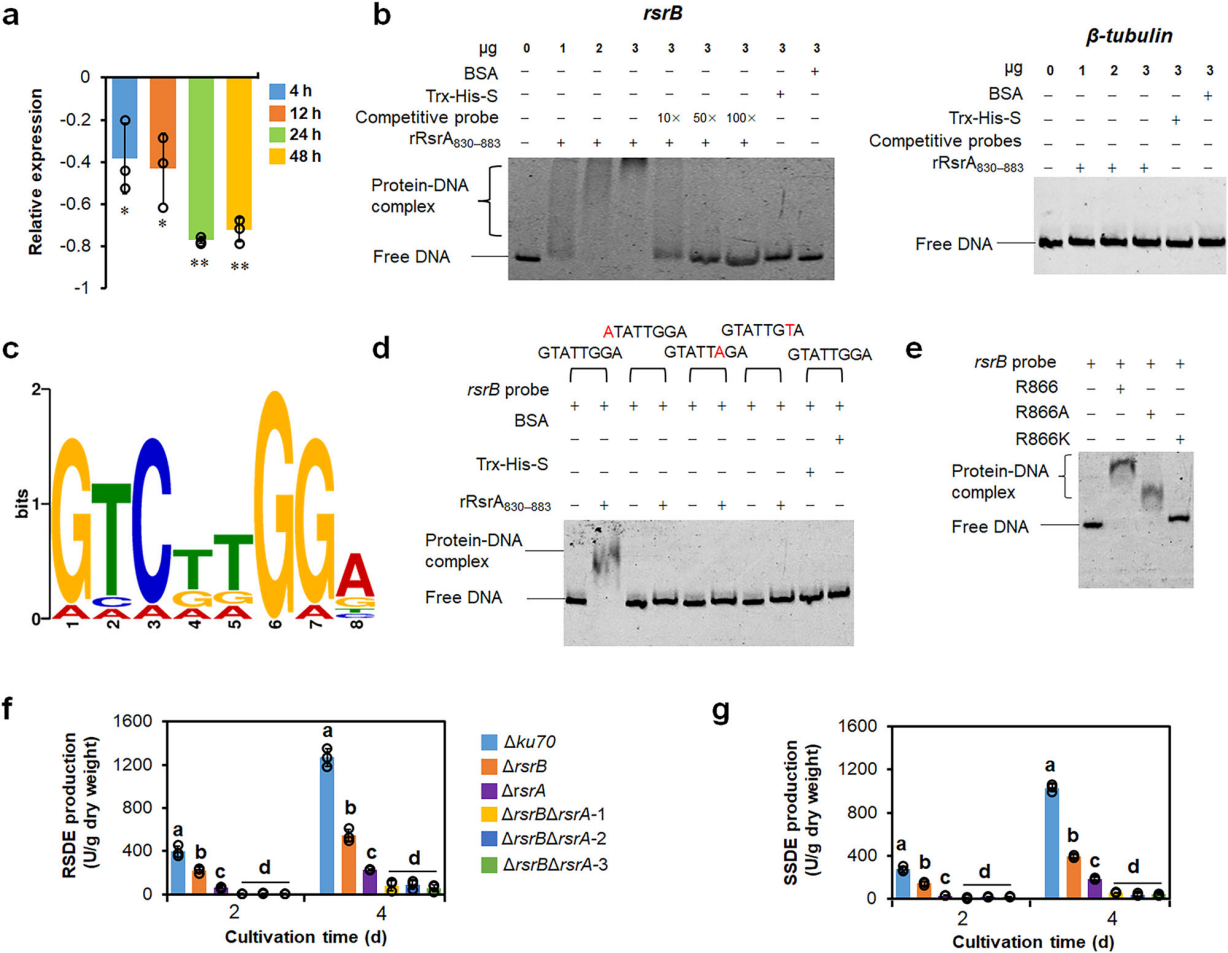
Analysis of the regulatory relationship between RsrA and RsrB. **a** Real-time reverse transcription quantitative PCR (RT-qPCR) indicating expression of *rsrB* in mutant Δ*rsrA* on soluble corn starch (SCS). *P*. *oxalicum* strains were cultured for 4–48 h after transfer. mRNA levels in mutant Δ*rsrA* were normalised to the levels in parental strain Δ*ku70* at the corresponding timepoints. **p < 0.01 and *p < 0.05 by Student’s *t* test represent significant differences between deletion mutant Δ*rsrA* and Δ*ku70*. Results are means ± SD. **b** In vitro EMSA between RsrA and *rsrB* probe. Recombinant rRsrA_830–883_ (0–3 μg) and *rsrB* probe (50 ng) were loaded. Trx-His-S peptide and bovine serum albumen (BSA) served as controls, along with the promoter region of the *β-tubulin* gene. Competitive probes were DNA fragments without 6-carboxyfluorescein. **c** MEME-predicted conserved DNA sequences bound by rRsrA_830–883_. DNA sequences were from the promoter regions of *PoxGA15A*, *POX_b02418, amy13A* and *rsrB*. **d** EMSA indicating the binding of mutated *rsrB* probes by rRsrA_830–883_ (3 μg). Mutated probes have ‘A’ or ‘T’ instead of ‘G at the 1st, 6th and 7th positions, as shown in (**c**). **e** EMSA between mutated rRsrA_830–883_ (3 μg) and *rsrB* probes. In mutated rRsrA_830–883_, R866 is exchanged for A or K. ‘–’ indicates no protein added. (**f, g**) Production of RSDEs and SSDEs by *P. oxalicum* parental strain Δ*ku70*, deletion mutants Δ*rsrB* and Δ*rsrA*, and double mutant Δ*rsrB*Δ*rsrA*. These strains were cultured in medium containing SCS for 2–4 days after transfer from glucose. Each mutant included three independent transformants. Results are mean ± SD. All experiments were performed at least three times. Lowercase letters represent *p* < 0.05. Different letters indicate significant differences, evaluated by one-way ANOVA. RSDE raw starch-degrading enzyme, SSDE soluble starch-degrading enzyme.

Comparative transcriptomic analysis found that the transcript abundance of the *rsrB* gene significantly decreased by 67.8% in Δ*rsrA* relative to Δ*ku70*^18^. RT-qPCR analysis further confirmed that *rsrB* transcription decreased by 38.4%–76.9% after deletion of *rsrA* when cultivated in medium containing SCS for 4–48 h (Fig. 4a). In addition, in vitro EMSA indicated that recombinant rRsrA_830-883_^19^ formed a complex with 6-FAM-tagged *rsrB* probe, and the formed complex markedly decreased or disappeared when untagged competitive probe was loaded. The shifted bands did not appear when loading rRsrA_830-883_ and control *β-tubulin* gene probes, or between control protein BSA or Trx-His-S and tested probes (Fig. 4b). These results showed that RsrA specifically binds to the promoter region of *rsrB*.

Previous studies found that RsrA binds to the core DNA sequence 5’-RHCDDGGD-3’ (R: G/A; H: T/C/A; D: T/G/A)^19^. Analysis using the MEME suite indicated that *rsrB*_-292–-285_ (5’-GTATTGGA-3’) might be required for RsrA binding, and three nucleotides (G1, G6 and G7) were relatively conserved (Fig. 4c). When they were mutated to A, A and T, respectively, the mutated probes couldn’t be bound by RsrA (Fig. 4d), suggesting that these residues are essential for the target sequence to be bound by RsrA.

In addition, residue R866 in RsrA is required for binding DNA^19^. As expected, in vitro EMSA found a clear shifted band when loading rRsrA_830–883_ and *rsrB* probes, but not between mutated rRsrA_830–883_^R866K^ and *rsrB* probe. Notably, a weak shifted band appeared when loading a mixture of mutated rRsrA_830-883_^R866A^ and *rsrB* probe (Fig. 4e). These results show that residue R866 of RsrA is required for binding to the promoter region of *rsrB*.

Double deletion mutant Δ*rsrB*Δ*rsrA* was successfully constructed (Supplementary Fig. S9), and its secreted raw starch-degrading enzyme production was 86.1% and 66.8% less than that of mutants Δ*rsrB* and Δ*rsrA*, respectively (Fig. 4f), and raw starch-degrading enzyme and soluble starch-degrading enzyme production was also diminished by 87.8% and 74.2% (Fig. 4g). SDS-PAGE analysis indicated that the extracellular proteins by Δ*rsrB*Δ*rsrA* was lower than that by mutants Δ*rsrB* and Δ*rsrA* when cultivated for 2 or 4 days (Supplementary Fig. S9c).

When cultivated on solid plates containing PDA, glucose and commercial starch of corn, the colony diameter of mutant Δ*rsrB*Δ*rsrA* was comparable to that of ∆*rsrA* but larger than that of Δ*ku70* and Δ*rsrB* on PDA. However, the diameter of Δ*rsrB*Δ*rsrA* was shorter than that of Δ*rsrB* on glucose and commercial starch of corn (Supplementary Fig. S4a). Moreover, number of asexual spores by Δ*rsrB*Δ*rsrA* was similar to that of ∆*rsrA*, but reduced by 70.0–88.0% compared with Δ*rsrB* on PDA, glucose and commercial starch of corn (Supplementary Fig. S4b). Microscopy revealed that hyphae of mutant Δ*rsrB*Δ*rsrA* were similar to Δ*rsrA* (Supplementary Fig. S5).

### POX_a01508 specifically binds to the promoter region of *rsrA*

To screen for proteins binding to the promoter region of *rsrA*, a DNA fragment without autoactivation activity was required for Y1H assay. We artificially designed 12 different lengths of DNA fragments upstream of the *rsrA* start code ATG, namely R1 to R12 (Supplementary Fig. S10a,) and used them as baits for self-activation testing. The results showed that all tested yeast strains grew well on synthetic deficiency medium (SD)/-Ura. After adding aureobasidin A (AbA), growth of yeast strains Y1H/R11 and Y1H/R12 was gradually inhibited with an increasing amount of AbA, as was that of the Y1H/p53 strain, whereas the other Y1H/R1 to Y1H/R10 was almost unaffected. Furthermore, when AbA was added at 150 ng/mL or more, Y1H/R12 exhibited no growth, indicating that R12 did not have a self-activation effect and could be used for subsequent Y1H screening (Supplementary Fig. S10b).

**Fig. 5:**
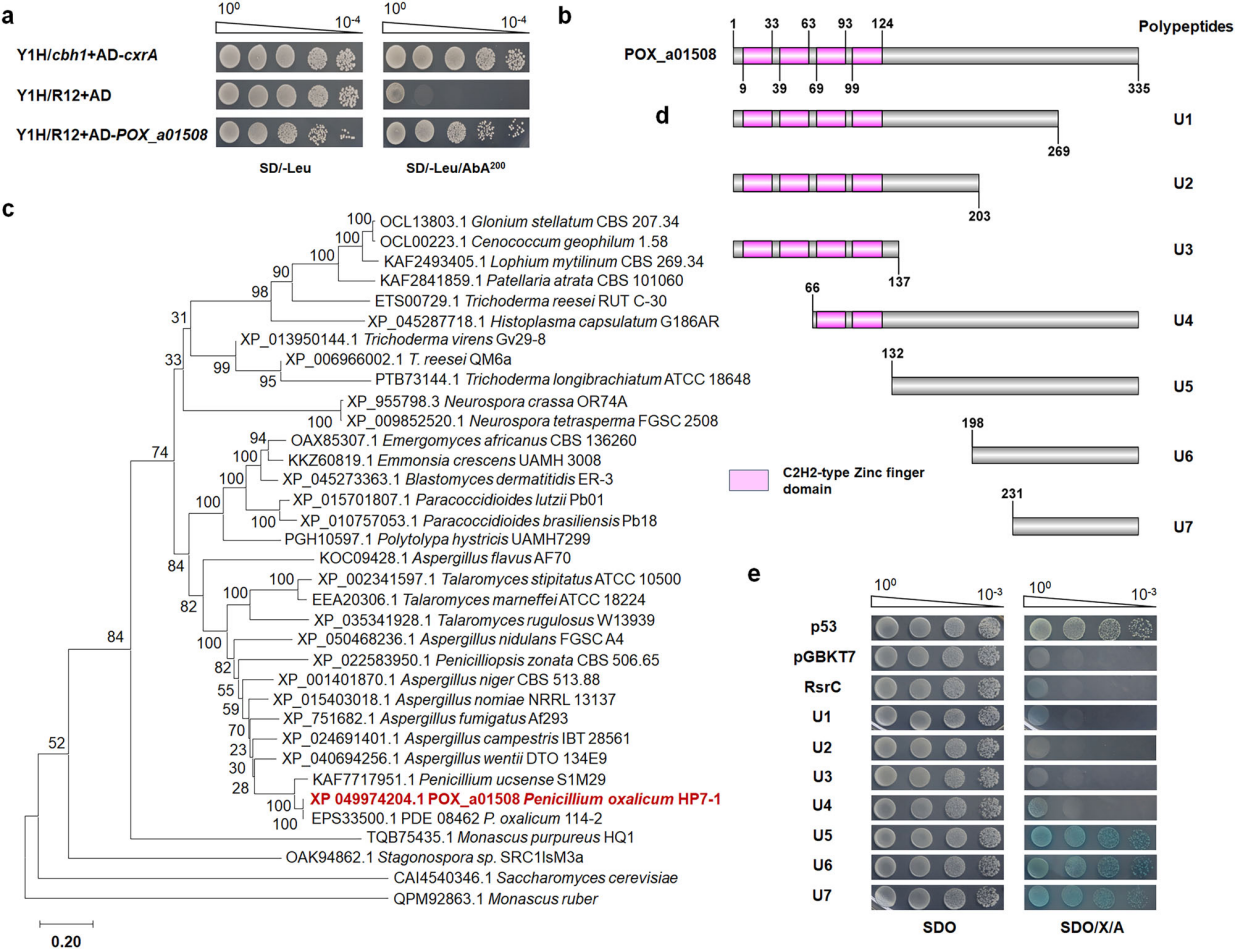
Effects of RsrC (POX_a01508) on binding to the promoter region of *rsrA*, sequence and phylogenetic analyses, and measurement of transcriptional activation ability. **a** Y1H assay. Yeast cells carrying DNA fragments upstream of *rsrA* were cultured on SD/-Leu and SD/-Leu/AbA^200^ for 3 days. **b** Conserved domains in RsrC. Purple areas indicate Cys2His2 (C2H2)-type zinc finger domains. **c** Phylogenetic analysis of RsrC and its homologues. The cladogram was constructed by MEGA version X with the neighbour-joining method and a Poisson model. Values displayed on branches were calculated using 1000 bootstrap replicates. Yeast self-activation assay. Yeast cells carrying different lengths of RsrC peptides (**d**) were cultured on SDO (SD/-Trp) and SDO/X/A (SD-Trp/+ X-α-Gal /+ aureobasidin A) for 3 days (**e**).

A cDNA library comprising 287 putative TF genes from *P*. *oxalicum* strain HP7-1, in which cDNA fragments were integrated into plasmid pGADT7, was constructed for Y1H assay. These 287 recombinant plasmids were individually transformed into yeast strain Y1H/R12 to screen for interacting proteins. When cultured on both SD/-Leu and SD/-Leu/AbA for 3 days, only the Y1H strain containing R12 and pGADT7-*POX*_*a01508* grew normally, consistent with positive strain Y1H/*cbh1*/pGADT7-*cxrA*. TF CxrA was demonstrated to specifically bind the promoter of cellobiohydrolase gene *cbh1*^23^. The negative control Y1H/*cbh1*/ pGADT7 only grew normally on SD/-Leu, but not on SD/-Leu/AbA (Fig. 5a). Therefore, POX_a01508 likely binds specifically to the promoter region of *rsrA*.

### RsrC is a C2H2-type TF

The gene *POX_a01508* is 1329 bp in length, containing three introns and encoding a polypeptide with 335 amino acids. The POX_a01508 protein contains four Cys2His2 (C2H2)-type zinc finger domains based on SMART analysis online (Fig. 5b). Furthermore, POX_a01508 shared 100% sequence identity with PDE_08462 (EPS33500.1) in *P*. *oxalicum* strain 114-2, which has no known function, followed by PECM_003408 from *Penicillium ucsense* strain S1M29 with an identify of 88.72%. By contrast, POX_a01508 shares lower identity (<70%) with homologues from other filamentous fungi such as *Fusarium*, *Aspergillus* and *Trichoderma*. Phylogenetic tree analysis showed that POX_a01508 was closely related to orthologs in *Penicillium* and *Aspergillus* species, while distantly related to those in *Trichoderma*, *Fusarium* and *Neurospora* (Fig. 5c). For convenience in future research, POX_a01508 was re-named as RsrC (Production of **r**aw-**s**tarch-degrading enzyme **r**egulator **C** in *Penicillium oxalicum*).

To test the transcriptional activation activity of RsrC, seven peptides of different lengths, namely U1 to U7, were designed as shown in Fig. 5d. DNA fragments encoding U1 to U7 were ligated to linearised pGBKT7 vector. The resulting recombinant plasmids were transformed into yeast Y2HGold competent cells. The obtained transformants were plated on SDO and SDO/X/A and incubated at 30 °C for 3 days. All colonies grew normally on SDO, indicating successful introduction of plasmids into Y2HGold cells. Strains Y2H/*rsrC*, Y2H/U1, Y2H/U2, Y2H/U3 and Y2H/U4 did not grow on SDO/X/A, consistent with the negative control. On the other hand, strains Y2H/U5, Y2H/U6 and Y2H/U7 grew normally and turned blue (Fig. 5e), indicating that U5, U6, and U7 can activate transcription of reporter genes *AUR1-C* and *MEL1* in the Y2HGold cells, demonstrating a transcriptional activation function.

### RsrC positively regulates amylase production

**Fig. 6:**
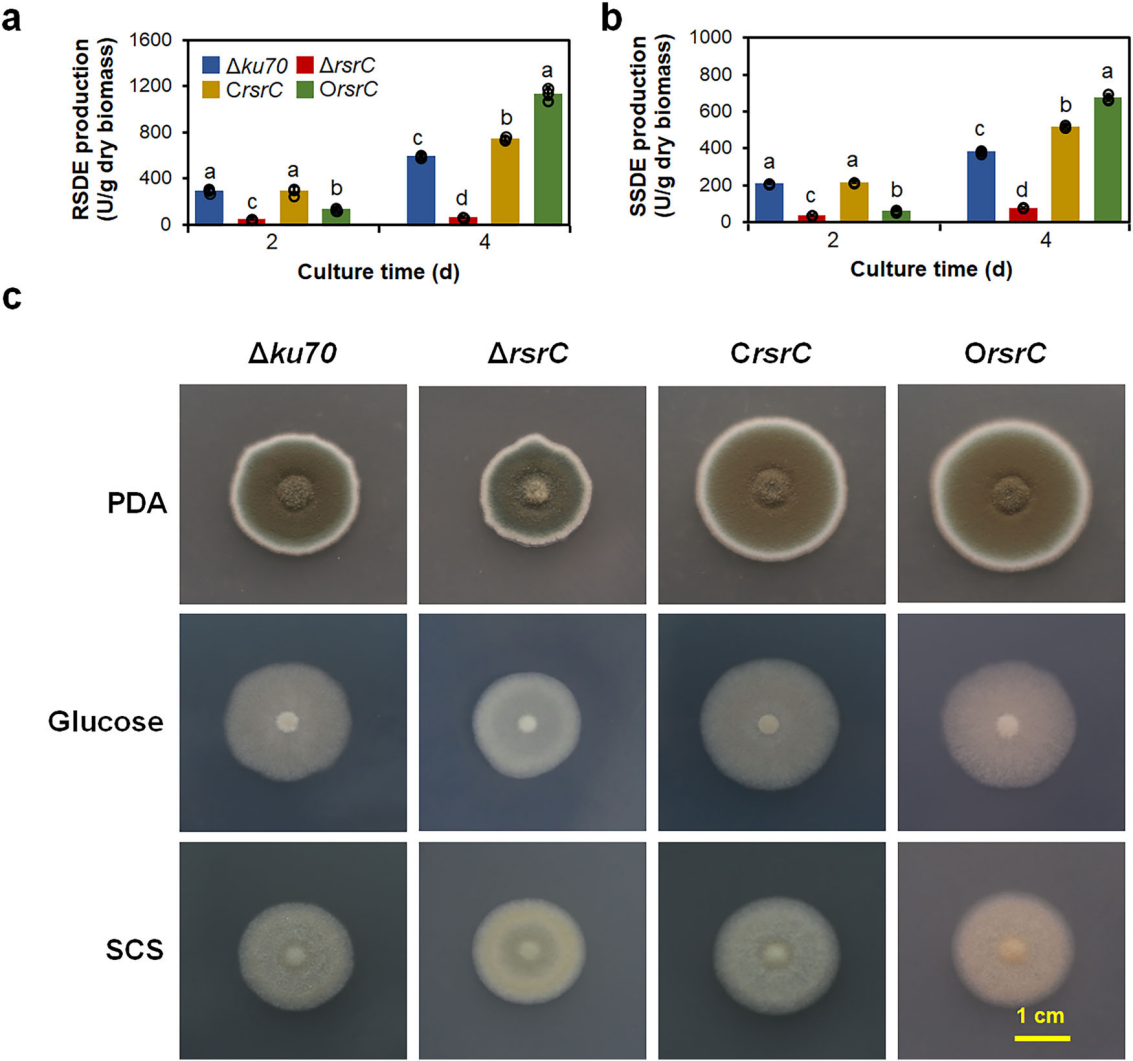
Effects of RsrC on amylase production and phenotypes of *P*. *oxalicum.* **a** Production of RSDEs and SSDEs (**b**) by *P*. *oxalicum* mutant Δ*rsrC*, parental strain Δ*ku70*, complementation strain C*rsrC* and overexpression strain O*rsrC* in the presence of SCS. Lowercase letters represent *p* < 0.05. Different letters indicate significant differences, evaluated by one-way ANOVA. **c** Colony observation of *P*. *oxalicum* mutant Δ*rsrC*, parental strain Δ*ku70*, complementation strain C*rsrC* and overexpression strain O*rsrC* on PDA, glucose and SCS plates for 4–5 days.

To explore the role of RsrC in amylase biosynthesis in *P*. *oxalicum*, deletion mutant Δ*rsrC* was constructed and confirmed by PCR (Supplementary Fig. S11a, b). Compared with Δ*ku70*, mutant Δ*rsrC* exhibited a significant decrease in production of raw starch-degrading enzymes and soluble starch-degrading enzymes, ranging from 79.0% to 85.9% (Fig. 6a, b). Enzyme production by C*rsrC* (Supplementary Fig. S11c and d) was comparable to that by Δ*ku70* after 2 days of induction, but considerably enhanced at 4 days (Fig. 6a, b).

Moreover, the *rsrC* DNA cassette was integrated into the genome of *P. oxalicum* strain Δ*ku70* at the *POX_d05452* gene locus to generate overexpression strain O*rsrC*, in which expression of *rsrC* was activated by constitutive promoter Ptef1 (Supplementary Fig. S11e, f). Further RT-qPCR assay revealed that the transcriptional level of *rsrC* was considerably enhanced by 0.38–2.33-fold in O*rsrC* relative to Δ*ku70* (Supplementary Fig. S11g). When cultivated on commercial starch of corn for 4 days, raw starch-degrading enzyme and soluble starch-degrading enzyme production of overexpression strain O*rsrC* markedly increased by 90.2% and 76.3%, respectively, whereas at 2 days enzyme production decreased by 54.2% and 69.6% (Fig. 6a, b). SDS-PAGE analysis showed that mutant Δ*rsrC* secreted less extracellular proteins than the Δ*ku70* when cultivated for 2 or 4 days, whereas the overexpression strain O*rsrC* secreted more extracellular proteins (Supplementary Fig. S11h). These results suggest that RsrC positively regulates the production of amylase in *P. oxalicum*, especially during the later stages of induction.

### RsrC affects mycelial growth of *P*. *oxalicum*

When inoculated onto solid plates containing PDA, glucose and commercial starch of corn as carbon sources, and incubated for 4–5 days, the colonies of Δ*rsrC* exhibited colour changes to various degrees, and they were considerably smaller than those of Δ*ku70* or C*rsrC*. Comparatively, overexpression of *rsrC* also caused a change of colony colour, but colonies became larger. However, the colony colour and size of complemented strain C*rsrC* were partially restored to those of Δ*ku70* (Fig. 6c).

### RsrC widely regulates gene expression on commercial starch of corn

Both parent Δ*ku70* and mutant Δ*rsrC* were separately cultured in medium containing commercial starch of corn for 24 h after transfer, and total RNAs were extracted and used for transcriptome sequencing analysis. The vales ranging from 0.97 to 0.99 for each sample (Supplementary Fig. S8b), indicating that the transcriptome data were reliable and could be used for further data analysis.

**Fig. 7:**
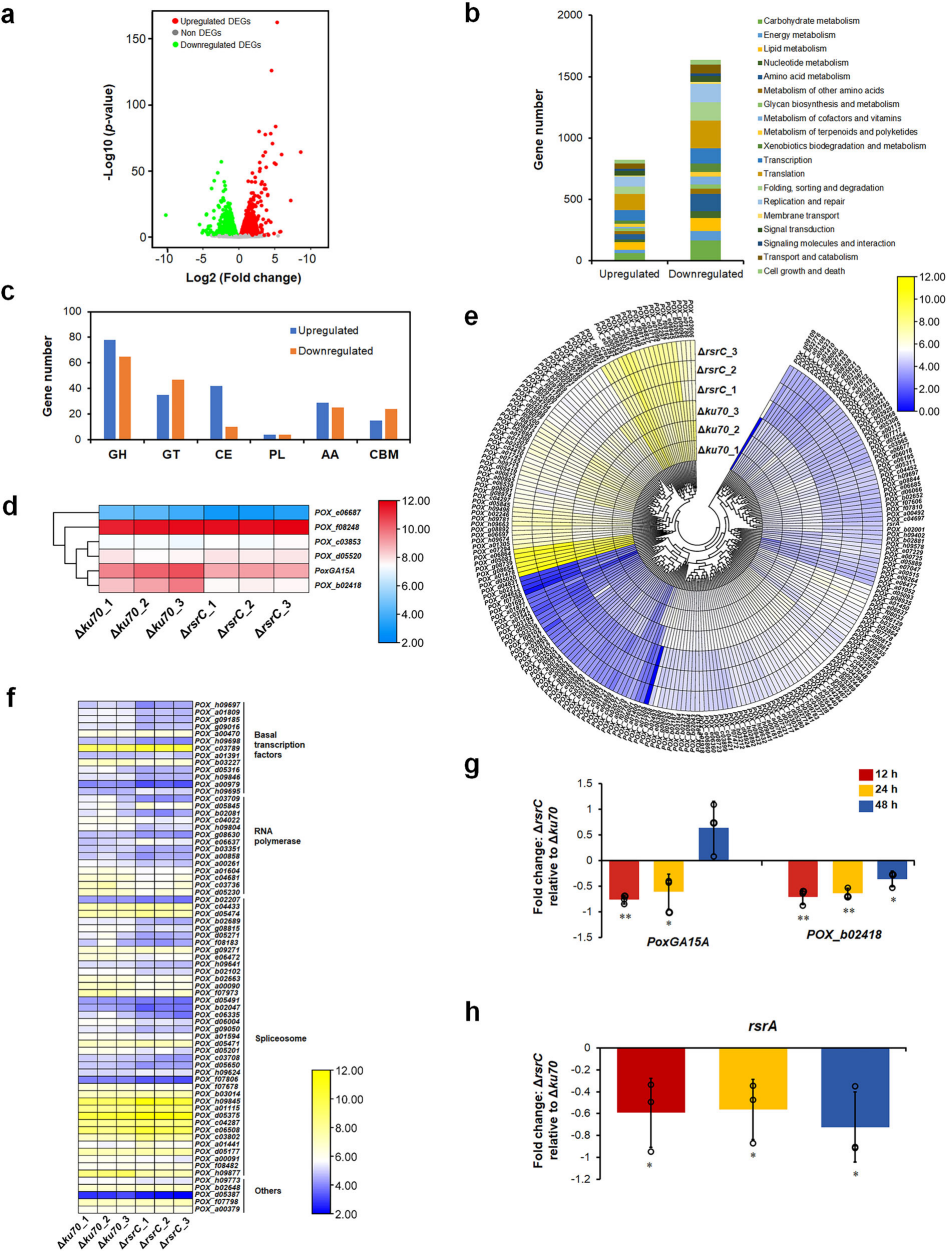
Effects of RsrC on gene expression in *P. oxalicum* in the presence of SCS. **a** Volcano plot showing DEGs. DEGs were selected with threshold *p* ≤ 0.05. **b** KEGG annotations of DEGs modulated by RsrC. **c** DEGs encoding carbohydrate-active enzymes (CAZymes). **d** DEGs encoding amylolytic genes. **e** DEGs encoding putative transcription factors. **f** DEGs encoding factors related to gene transcription. **g** RT-qPCR analysis of genes encoding major amylases, as well as key regulatory gene *rsrA* (**h**). All *P*. *oxalicum* strains were cultured for 12–48 h in the presence of SCS. Gene expression in Δ*rsrC* was normalised to the level of Δ*ku70*. Results are mean ± SD. **p < 0.01 and *p < 0.05 indicate significant differences between Δ*rsrC* and Δ*ku70*, calculated by Student’s t test. PoxGA15A, raw s*t*arch-degrading glucoamylase; POX_b02418, glucoamylase.

Gene expression levels were compared between mutant Δ*rsrC* and parental strain Δ*ku70*, using *p* value < 0.05 as the screening criterion. Based on the annotation of genome of *P*. *oxalicum* strain HP7-1^22^, in mutant Δ*rsrC*, 4137 genes showed marked differences in expression relative to those in Δ*ku70* (Supplementary Data 2). Among these genes, 2059 were significantly upregulated (0.23 <Log2Fold change <8.61), while 2078 were significantly downregulated (-10.2 <Log2Fold change <-0.22; Fig. 7a). These DEGs encoded proteins mainly involved in metabolic processes, particularly carbohydrate metabolism (accounting for 16.7%) and amino acid metabolism (13.5%), followed by genetic information processes including translation (10.9%; Fig. 7b).

Among the DEG regulon of *rsrC*, 345 DEGs encoding carbohydrate-active enzymes (CAZymes) were detected in Δ*rsrC*, including 189 upregulated (0.24 <Log_2_Fold change <4.03) and 156 downregulated (-4.04 <Log_2_Fold change <-0.22) genes. These DEG-encoding proteins that could be classified into six CAZyme families, namely glycosyltransferase (GT; 24.63%), auxiliary oxidoreductase (AA; 15.65%), glycoside hydrolase (GH; 41.45%), carbohydrate esterase (CE; 14.49%), carbohydrate-binding module (CBM 11.30%) and polysaccharide lyase (PL; 2.31%; Fig. 7c). Interestingly, among these DEGs encoding glycoside hydrolases, six genes encoding amylolytic enzymes were found in mutant Δ*rsrC* relative to the parental strain Δ*ku70*, including *PoxGA15A*, *POX_b02418*, 1,4-α-glucan branching enzyme gene *POX_d05520*, and three α-glucosidase genes *POX_f08248*, *POX_c03853* and *POX_e06687*. The transcriptional levels of three genes decreased in Δ*rsrC*; *PoxGA15A* by 49.7%, *POX_b02418* by 63.7% and *POX_e06687* by 55.2% (Fig. 7d).

Additionally, there were 225 genes encoding specific TFs, most containing conserved zinc finger domains (146), followed by winged helix repressor DNA-binding domains (27). Among these, 62 genes showed upregulation (0.25 <Log2 fold change <4.13), while 163 genes showed downregulation in Δ*rsrC* (-10.16 <Log2 fold change <-0.28). Notably, four known regulatory genes (*creA*/*POX_e07192*^24^, *rsrA*/*POX_a00019* and *rsrB*) were identified, and the former one repressed expression of amylase genes, whereas the latter two activated expression^18^. Expression of both *rsrA* and *creA* in Δ*rsrC* was downregulated by 23.2% and 27.7%, respectively, whereas *rsrB* were upregulated by 26.2% (Fig. 7e).

In addition to the specific TFs, other factors relative to gene transcription, such as RNA polymerase complex, basal TFs, and the spliceosome complex^25^, were screened from proteins encoded by DEGs in the *rsrC* regulon. The results revealed 71 relative proteins including 14 RNA polymerase subunits (including RNA polymerase I subunit RPA1, RPA12 and RPA12; RNA polymerase II subunit RPB1 and RPB7, RNA polymerase III subunits RPC1, RPC2, RPC3, RPC7 and RPC11, RNA polymerases I and III subunits RPAC1 and RPAC2; RNA polymerases I, II, and III subunits RPABC1 and RPABC3), 11 basal TFs (including transcription initiation factor TFIID subunits TAF1, TAF2, TAF3, TAF6, TAF7, TAF9, TAF12 and TAP), as well as 40 spliceosome-relative proteins including pre-mRNA-processing factors PRP8, PRP19, PRP43, SLU7 and SLT11; and U4/U6 small nuclear ribonucleoprotein PRP3, PRP4 and PRP31. More than 60% of these were downregulated in Δ*rsrC*, especially RNA polymerase subunits (Fig. 7f).

Absorption of sugars by fungal cells is dependent on sugar transporters. Screening annotation of 4137 DEGs revealed 36 genes encoding sugar transporters, and comparative analysis identified 14 genes that were significantly upregulated (0.42 <Log2 fold change <3.85) in Δ*rsrC*, while 22 were significantly downregulated (-5.46 <Log2Fold change <-0.37). Notably, the predominant cellulose dextrin transporter gene *cdtC* was downregulated by 58.8% (Supplementary Data 2).

Moreover, RT-qPCR analysis was employed to confirm the results from comparative transcriptomics. The results showed that, compared with parental strain Δ*ku70*, the expression level of *PoxGA15A* in Δ*rsrC* was considerably downregulated, by 75.6% and 42.8% after 12 and 24 h of cultivation, respectively. Additionally, the expression level of *POX_b02418* was downregulated by 58.9%, 69.3% and 26.9% after 12, 24 and 48 h of cultivation, respectively (Fig. 7g). As expectedly, expression of *rsrA* decreased by 47.3–90.9% in Δ*rsrC* (Fig. 7h). The results at 24 h were consistent with data from comparative transcriptomics.

### RsrC positively regulates *rsrA* expression to promote amylase production

**Fig. 8:**
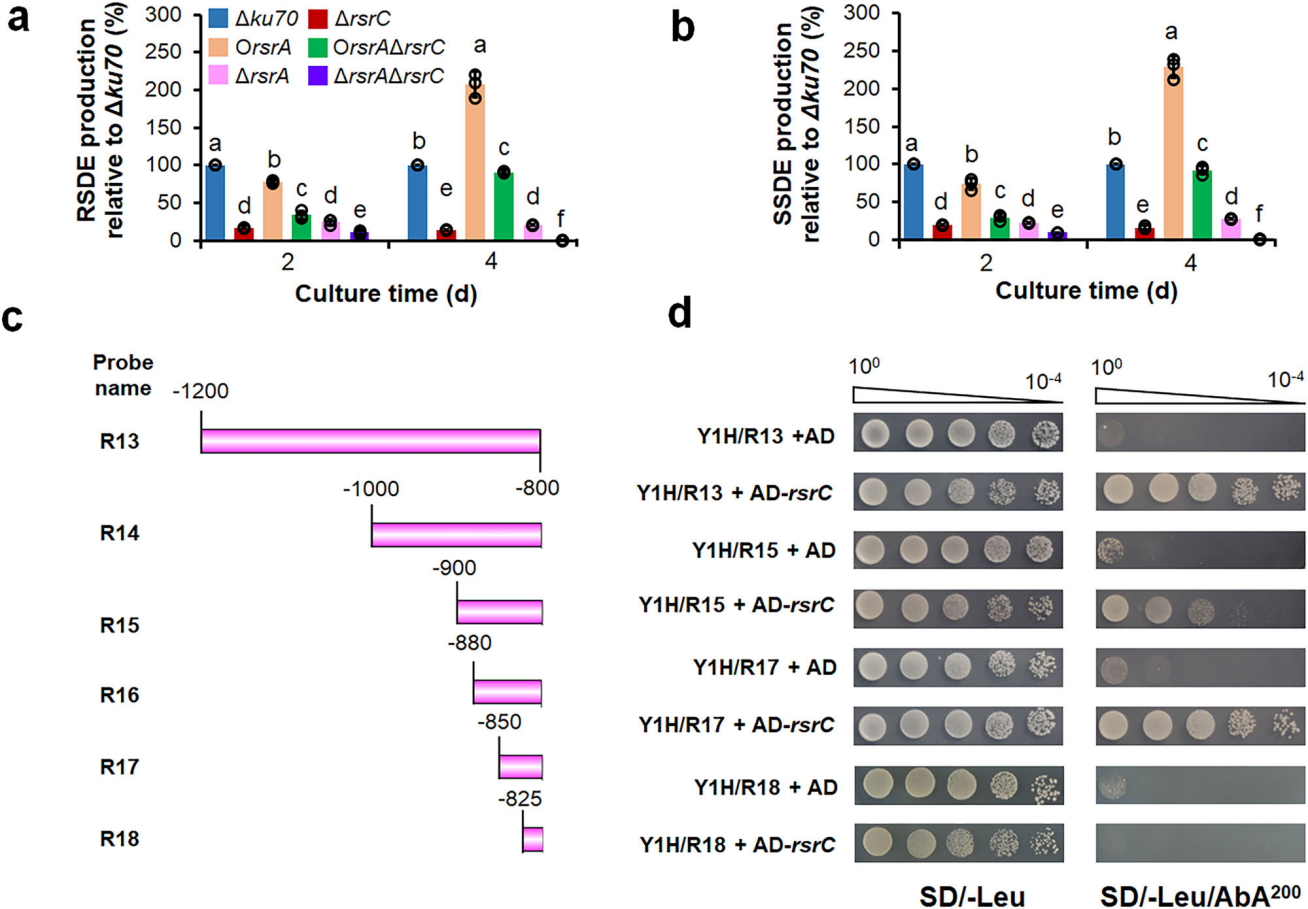
Analysis of regulatory relationship between RsrC and RsrA. **a**, **b** Production of RSDEs and SSDEs by *P. oxalicum* parental strain Δ*ku70*, deletion mutants Δ*rsrC* and Δ*rsrA*, overexpression strain O*rsrA*, mutant O*rsrA*Δ*rsrC*, and double mutant Δ*rsrA*Δ*rsrC*. These strains were cultured in medium containing soluble corn starch for 2–4 days after transfer from glucose. Each mutant included three independent transformants. Results are mean ± SD. All experiments were performed at least three times. Lowercase letters represent *p* < 0.05. Different letters indicate significant differences, evaluated by one-way ANOVA. RSDE, raw starch-degrading enzyme; SSDE, soluble starch-degrading enzyme. **c** Schematic diagram indicating the truncated promoter region of *rsrA* for Y1H assay. **d** Y1H assay between RsrC and the truncated region of upstream DNA sequence of *rsrA*. Yeast cells carrying DNA fragments upstream of *rsrA* are cultured on SD/-Leu and SD/-Leu/AbA^200^ for 3 days.

To investigate the regulatory relationship between RsrA and RsrC concerning the amylase production in *P. oxalicum*, mutant O*rsrA*Δ*rsrC* was constructed and confirmed by PCR (Supplementary Fig. S12a, b), using O*rsrA* as the background strain. In the O*rsrA*, the expression of *rsrA* inserted was promoted by the constitutive promoter Ptef1, and native *rsrA* transcription was controlled by its own promoter^19^. Compared with control strain O*rsrA*, O*rsrA*Δ*rsrC* exhibited a significant decrease of 56.2–60.1% in the production of raw starch-degrading enzymes and soluble starch-degrading enzymes, when cultured in commercial starch of corn medium for 2–4 days after transfer. Conversely, compared with Δ*rsrC*, there was a significant increase of 1.2–7.1-fold (Fig. 8a, b). SDS-PAGE analysis showed that the secreted extracellular proteins by O*rsrA*Δ*rsrC* decreased in comparison with the control strain O*rsrA* when cultivated for 2 or 4 days (Supplementary Fig. S12e). This suggests that RsrC positively regulates the expression of RsrA to promote the amylase production.

In addition, double mutant Δ*rsrA*Δ*rsrC* was also successfully constructed (Supplementary Fig. S12c, d). Analysis of enzymatic activity revealed that during 2–4 days on commercial starch of corn, Δ*rsrA*Δ*rsrC* showed a considerably decrease in production of raw starch-degrading enzymes and soluble starch-degrading enzymes compared with all control strains on day 2, and even produced negligible amylase on day 4 (Fig. 8a, b). SDS-PAGE analysis indicated that the Δ*rsrA*Δ*rsrC* secreted reduced extracellular proteins compared with all control strains when cultivated for 2 or 4 days (Supplementary Fig. S12e). These results indicate that RsrA and RsrC specifically regulate the biosynthesis of amylase, and their regulation clearly overlaps.

### Residues -850 to -825 bp upstream of *rsrA* are required for RsrC binding

To identify the core DNA sequence in the promoter region of *rsrA* that was bound by RsrC, a series of truncated DNA fragments, R13 to R18, were designed as baits for Y1H assay (Fig. 8c). Recombinant plasmid pGADT7-*rsrC* and control pGADT7 were separately transformed into yeast bait strains Y1H/R13, Y1H/R15, Y1H/R17 and Y1H/R18, and plasmid pGADT7-*cxrA* was transformed into Y1H/*cbh1* as a positive control. All obtained yeast transformants were able to grow normally on SD/-Leu, indicating successful transformation of plasmids into bait strains. By comparison, on SD/-Leu/AbA^200^, Y1H/R13/pGADT7-*rsrC*, Y1H/R15/pGADT7-*rsrC* and Y1H/R17/pGADT7-*rsrC* showed normal growth, as did positive control Y1H*/cbh1*/pGADT7-*cxrA*, whereas Y1H/R18/pGADT7-*rsrC* and the Y1H/R13/pGADT7, Y1H/R15/pGADT7 and Y1H/R17/pGADT7 control strains could not grow (Fig. 8d). These results indicate that RsrC can bind to three DNA fragments (residues -1200 to -800, -900 to -800, and -850 to -800 bp) in the promoter region of *rsrA*, but not -825 to -800 bp, meaning that residues -850 to -825 bp are required for binding by RsrC.

### Comparative analysis of regulons of RsrA, RsrB and RsrC

To further investigate similarities and differences between the regulation of RsrA, RsrB and RsrC in *P*. *oxalicum*, we comparatively analysed their regulons under induction after 24 h by commercial starch of corn. Transcriptome profiling of mutant Δ*rsrA* in the presence of commercial starch of corn was assessed by RNA-seq. Sequencing data from three biological replicates of each fungal strain were evaluated statistically and yielded a high Pearson correlation coefficient (Supplementary Fig. S8a).

With thresholds of FDR < 0.05, there were 5656 DEGs in Δ*rsrA* compared with ∆*ku70*, including 1438 upregulated and 4218 downregulated genes, named the RsrA regulon (Supplementary Data 3). Of these, only gene *amy13A* encoding the key α-amylase was downregulated (log2 fold change = -2.53). There were 305 putative TF-encoding genes, 51 of which were upregulated and 254 were downregulated. Notably, the key regulatory genes *amyR* and *rsrC* were found, with 30.0%- and 60.06%-reduced expression in the Δ*rsrA*. Moreover, 50 putative sugar transporter-encoding genes were detected, seven of which were upregulated and 43 were downregulated.

**Fig. 9:**
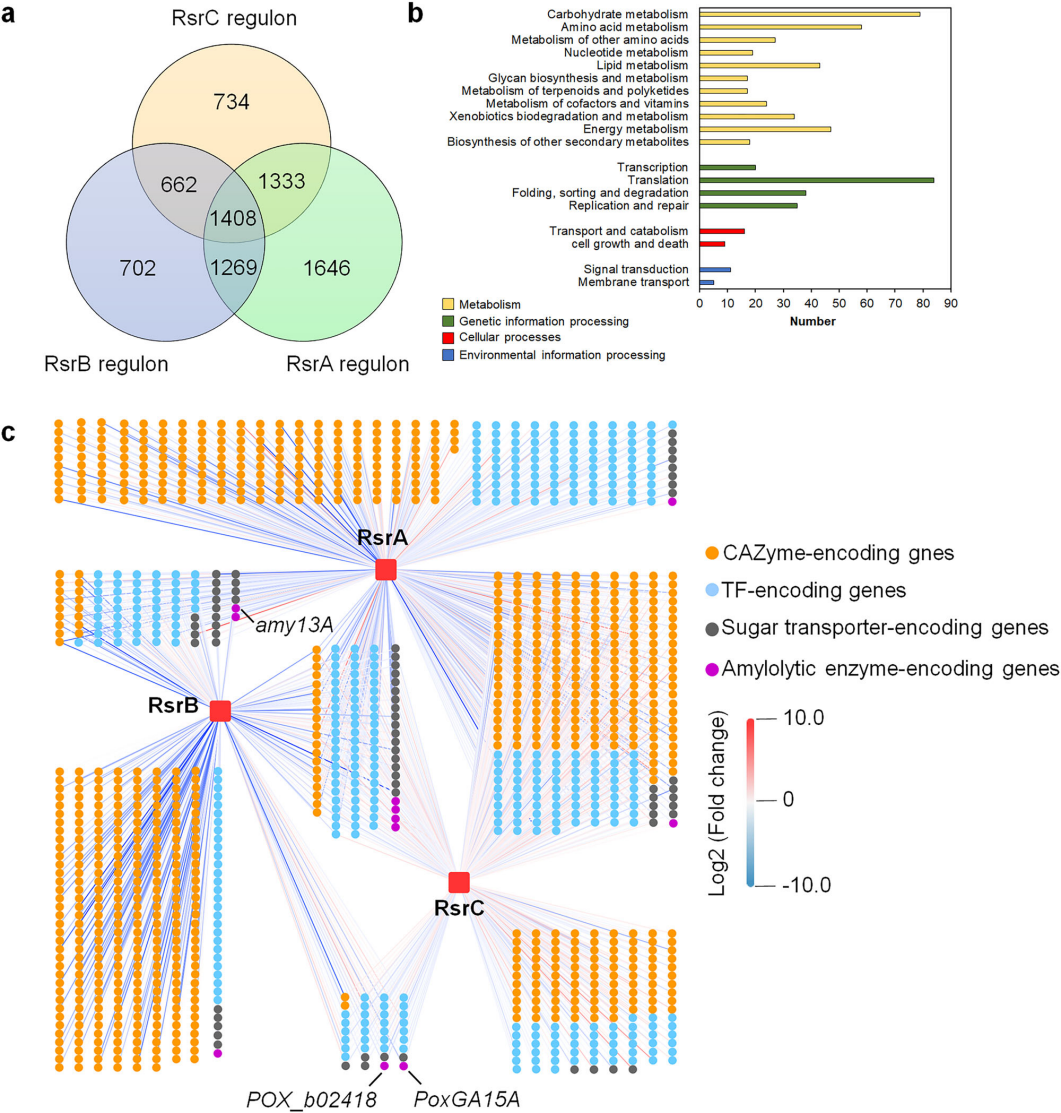
Co-regulation between RsrA, RsrB and RsrC in *P*. *oxalicum.* **a** Number of DEGs between RsrA, RsrB and RsrC regulons. Thresholds were set at False Discovery Rate ≤0.05 or *p* < 0.05. **b** KEGG annotations of genes co-regulated by RsrA, RsrB and RsrC. **c** Genes encoding CAZymes, TFs, sugar transporters and amylolytic enzymes regulated by RsrA, RsrB and RsrC.

Comparative analysis of RsrB and RsrA regulons identified 2677 DEGs co-regulated (Fig. 9a), of which 663 were upregulated and 1272 were downregulated in both Δ*rsrB* and Δ*rsrA*, relative to those in ∆*ku70* (Supplementary Fig. S13a). These co-regulated genes were mainly involved in metabolism and genetic information processing, especially carbohydrate, amino acid metabolism and translation (Supplementary Fig. S13b). Notably, the key *amy13A* was co-regulated, and its expression was downregulated (log2 fold change = -3.10 and -2.53) in Δ*rsrB* and Δ*rsrA*, respectively (Fig. 9b and Supplementary Fig. S13c). There were 125 co-regulated DEGs encoding TFs, 20 of which were upregulated and 74 were downregulated in both Δ*rsrB* and Δ*rsrA* (Fig. 9c and Supplementary Fig. S13d). Moreover, 33 co-regulated DEGs encoding putative sugar transporters were found, 26 of which were downregulated in both Δ*rsrB* and Δ*rsrA* (Fig. 9c and Supplementary Fig. S13e).

By contrast, 2741 DEGs co-regulated by RsrA and RsrC were detected (Fig. 9a), 938 of which were downregulated in both Δ*rsrA* and Δ*rsrC* compared with those in Δ*ku70*, while 425 were upregulated (Supplementary Fig. S14a). These co-regulated DEGs mainly participated in carbohydrate metabolism and translation (Supplementary Fig. S14b). Notably, 79 DEGs involved in ribosome biogenesis and assembly detected, of which 53 were upregulated in both Δ*rsrA* and Δ*rsrC* (Supplementary Fig. S14c). However, only three α-glucosidase genes *POX_c03853*, *POX_e06687* and *POX_g08885*, and one 1,4-α-glucan branching enzyme gene *POX_d05520* were included. Transcription of *POX_e06687* decreased (-3.11 <log2 fold change <-1.16) in both Δ*rsrA* and Δ*rsrC*, whereas expression of *POX_c03853* decreased (log2 fold change = -2.23) in Δ*rsrA* and increased (log2 fold change = 0.29) in Δ*rsrC*, expression of *POX_d05520* and *POX_g08885* decreased (-1.13 <log2 fold change <-0.80) in Δ*rsrA* but increased (0.31 <log2 fold change <1.38) in Δ*rsrC* (Fig. 9c and Supplementary Fig. S14d).

There were 2070 DEGs co-controlled by both RsrB and RsrC (Fig. 9a), including 401 upregulated and 307 downregulated genes in both Δ*rsrB* and Δ*rsrC* relative to those in Δ*ku70* (Supplementary Fig. S15a). These co-regulated genes were mainly involved in translation and carbohydrate metabolism (Supplementary Fig. S15b). Notably, two amylase genes, *PoxGA15A* and *POX_b02418*, were co-regulated, and their expression was downregulated in Δ*rsrB* and Δ*rsrC* (Fig. 9c and Supplementary Fig. S15c). In addition, among the 2070 DEGs, 96 TF-encoding genes (Fig. 9c and Supplementary Fig. S15d) and 23 sugar transporter-encoding genes (Fig. 9c and Supplementary Fig. S15e) were identified.

Comparative analysis of the three regulons descried above identified 1408 DEGs co-regulated by RsrA, RsrB and RsrC, of which 180 were upregulated and 212 were downregulated in Δ*rsrA*, Δ*rsrB* and Δ*rsrC*, relative to ∆*ku70* (Fig. 9a). These co-regulated genes were mainly involved in translation and carbohydrate metabolism (Fig. 9b). Among the 1408 DEGs, no gene encoding key amylases were detected. 69 co-regulated DEGs encoding TFs were identified. Of these, *POX_e06829*, *POX_g08739* and *POX_g08892* were upregulated and 24 were downregulated in Δ*rsrA*, Δ*rsrB* and Δ*rsrC* (Fig. 9c).

## Discussion

**Fig. 10:**
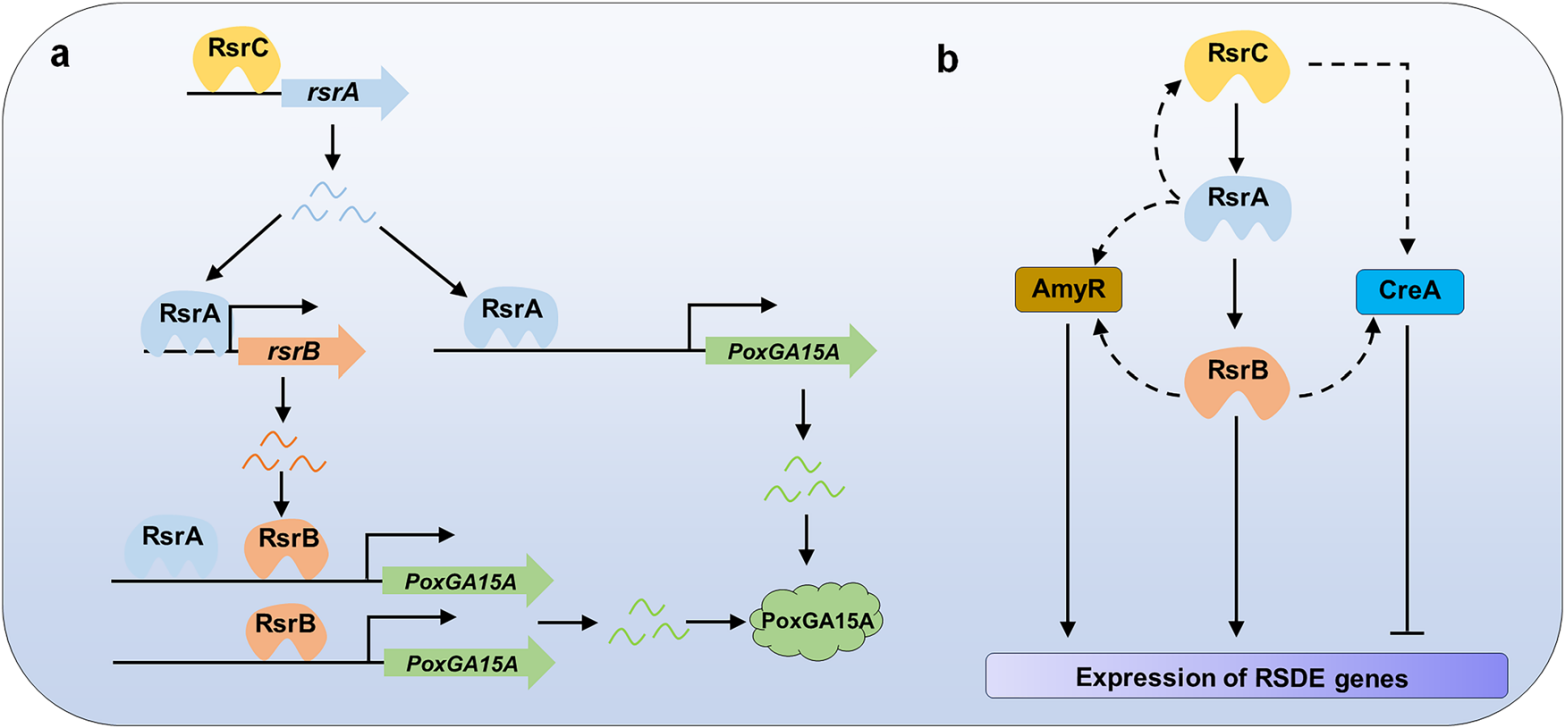
Proposed model of RsrC-RsrA-RsrB regulation in *P. oxalicum* in the presence of soluble corn starch. **a** Model of RsrC-RsrA-RsrB regulation. PoxGA15A, raw starch-degrading glucoamylase. **b** Regulatory network at 24 h of induction. Dashed lines indicate indirect regulation or non-confirmation.

In this study, we identified two TFs, RsrB and RsrC, acting downstream and upstream of RsrA, respectively, in *P. oxalicum*. RsrA is known to activate the expression of raw starch-degrading glucoamylase gene *PoxGA15A* and α-amylase gene *amy13A*^18,19^. RsrB and RsrC were found to regulate the biosynthesis of amylases including raw starch-degrading enzymes in the presence of commercial starch of corn, as well as conidiation and mycelial growth. Furthermore, RsrC stimulates the transcription of *rsrA* by directly binding to residues -850 to -800 bp in the promoter region of *rsrA*. RsrB can bind to the promoter regions of genes related to the biosynthesis of amylases and sporulation, thereby controlling their expression (Fig.10a). These findings enrich and expand the regulatory network of the expression of amylase genes.

Y2H assay indicated that RsrB_176–809_ has transcriptional activation ability, whereas RsrB_176–210_ and RsrB_211–809_ does not. RsrB_176–210_ is predicted to be the Gal4-like Zn2Cys6-type DNA-binding domain. In yeast, Gal4 contains two independently functional domains: an N-terminal DNA-binding domain and a C-terminal transcriptional activation domain^26^. However, RsrB_176–210_ is required for both DNA-binding and transcriptional activation ability. In addition, residues 132–335 in RsrC exhibited transcriptional activation activity. However, the full-length protein RsrB_1–809_ as well as RsrC_1–335_ do not demonstrate the transcriptional activation activity. The reason for these may be because protein folding and tertiary structure are different between yeast and *P. oxalicum*.

RsrA controls the expression of *PoxGA15A* and *amy13A* by directly binding to their promoter regions, which depends on R886. Notably, site-mutated R866A and R866K displayed different effects on raw starch-degrading enzyme production^18,19^. R866 was found to be required for RsrA binding to the *rsrB* promoter. Surprisingly, R866K lost the ability to bind *rsrB* probe, whereas R866A retained weak binding, but the reason is unknown. These differences in binding may explain why raw starch-degrading enzyme production by the R866A mutant was higher than that of R866K^19^.

Moreover, RsrA directly regulates the expression of *rsrB*, but RsrB cannot regulate the expression of *rsrA*. Double mutant Δ*rsrB*Δ*rsrA* exhibited lower amylase production than individual Δ*rsrB* and Δ*rsrA* strains. Additionally, the diameter of ∆*rsrB* colonies is smaller than that of control strain Δ*ku70*, but colonies of ∆*rsrA* were larger than that of Δ*ku70* on PDA. The ∆*rsrB* strain produced more asexual spores than Δ*ku70*, but this decreased for ∆*rsrA*. These results suggest that RsrA and RsrB have some overlap in the regulation of amylase biosynthesis and mycelial growth, but their independent regulatory pathways are not yet known, as confirmed by comparative analysis of their regulons. For example, there were 5656 DEGs in the *rsrA* regulon but only 4041 in the *rsrB* regulon, and 2677 were co-regulated by both RsrB and RsrA, including *amy13A*. However, RsrB regulated the expression of *brlA* and *POX_c03497/rodB* that participate in fungal growth and sporulation^16^, but RsrA did not.

Analysis of amino acid sequences identified RsrC as a C2H2-type zinc finger TF. In the literature, there are two other TFs containing a C2H2-type zinc finger domain, namely CreA^24^ and CxrB^27^, which participate in the biosynthesis of amylases in *P*. *oxalicum*. Interestingly, CreA mediates carbon catabolite repression, and suppresses the expression of amylase genes, whereas CxrB positively regulates the transcription of amylase genes. Additionally, CreA functions in both pathways, by directly binding to the promoter regions of genes encoding major RSDEs, and indirectly controlling the expression of other regulatory genes such as *amyR*^24^. CxrB indirectly controls the expression of amylases by directly regulating the transcription of *amyR*^27^. AmyR is known to be positively involved in amylase biosynthesis in fungi^24^. In addition, comparative transcriptomic analysis found that expression of *creA* was downregulated in Δ*rsrC*, whereas *rsrB* were upregulated. Therefore, RsrC controls the production of amylases via diverse cascade regulation. For instance, RsrC affects the expression of amylase genes, possibly by stimulating the expression of *rsrA* and thereby activating the transcription of *amyR* and *rsrB*; or activating the expression of *creA*, which inhibits the expression of amylase genes and other regulatory genes. Interestingly, comparative transcriptomics also revealed that the expression of *rsrC* is activated by *rsrA* (Fig. 10b). However, these regulations still require to be further confirmed through biochemical and genetic methods. Certainly, the final expression level of amylases gene in *P*. *oxalicum* depends on the balance of the regulatory network. Surprisingly, expression of *rsrB* was enhanced in Δ*rsrC*, suggesting that CreA may repress the expression of *rsrB* in the presence of commercial starch of corn.

Notably, the production of amylases by mutant O*rsrA*Δ*rsrC*, in which the additionally inserted *rsrA* is under the control of constitute promoter Ptef1, could partially restore the altered enzyme production caused by *rsrC* deletion. This indicates that RsrC regulation in the production of raw starch-degrading enzymes involves both RsrA-dependent and RsrA-independent pathways. In addition, double mutant Δ*rsrA*Δ*rsrC* exhibited considerably lower enzyme production than individual mutants Δ*rsrA* and Δ*rsrC*, suggesting that RsrA functions also involve RsrC-independent pathways. Furthermore, comparative analysis between RsrA and RsrC regulons revealed that the transcriptional abundance of genes *PoxGA15A* and *amy13A* was altered to different degrees in Δ*rsrA* and Δ*rsrC* relative to Δ*ku70*.

It should be noted that the promoter region of *rsrA* taken to identify the TF-binding site was very upstream, i.e., -800 to -1318. It is possible that the sites of some other important TFs might lie in the -1 to -800 region and it would be missed in this experiment. Notably, the region from -1 to -800 exhibited autoactivation activity, which was not suitable for Y1H assay.

Moreover, the production of raw starch-degrading enzymes and soluble starch-degrading enzymes by complementation strain C*rsrC* was considerably higher than that of parental strain Δ*ku70* when cultured in medium containing commercial starch of corn for 4 days. The C*rsrB* (*CPOX_g08691*) did not fully complement the phenotypes resulting from deletion of *rsrB*. Those might be due to different effects caused by the alternative locus (*POX_d05452*) integrated via the complementation cassette^22^.

Molecular breeding of fungi through synthetic biology is an efficient strategy to improve the production of proteins such as raw starch-degrading enzymes^28^. Overexpression of *rsrC* and *rsrB* markedly improved the production of amylases in *P. oxalicum*, indicating that RsrC and RsrB are good molecular targets for genetic engineering to enhance fungal enzyme production. It was recently confirmed that overexpression of *rsrA* could enhance amylase production^19^. Therefore, synergistic effect among RsrA, RsrB and RsrC should be considered in molecular breeding.

## Materials and methods

### *P. oxalicum* strains and culture conditions

All *P. oxalicum* strains used in this study are listed in Supplementary Table S1. *P. oxalicum* strains were cultured on PDA plates for 5 days at 28 °C to generate asexual spores. All asexual spores were washed with Tween 80 (Sangon Biotech Co., Ltd., Shanghai, China) and precultured in modified minimal medium containing glucose for 24 h. Approximately 1 g of mycelia was collected, transferred to modified minimal medium containing commercial starch of corn (Sigma-Aldrich, St. Louis, MO, USA), and cultured for 2–4 days to produce crude enzyme used for measurement of enzymatic production, or cultured for 4–48 h after transfer for RNA-seq and RT-qPCR assay.

Cultivation of *Saccharomyces cerevisiae* strains was conducted on yeast extract peptone dextrose for preservation and/or reproduction. Synthetic deficiency (SD) media without different nutrients was used for yeast autoactivation experiments and Y1H assay.

### Total DNA and RNA extraction from *P. oxalicum*

Mycelia of *P. oxalicum* were fractured by liquid nitrogen, dissolved in DNA extraction buffer for 15 min, and phenol-chloroform (phenol, Solarbio Life Sciences, Beijing, China; chloroform, Kelon Chemicals Co. Ltd., Chengdu, China) was used for protein removal. DNA was precipitated by anhydrous ethanol (Tianjin Fuyu Fine Chemicals Co., Ltd., Tianjin, China). Total RNA extraction from *P. oxalicum* was conducted using an RNAsimple Total RNA Kit (Tiangen Biotech Co., Ltd., Beijing, China) according to the manufacturer’s instructions.

### Yeast autoactivation experiment

DNA fragments were amplified by PCR with specific primers (Supplementary Table S2) using Δ*ku70* cDNA as template, then subcloned into vector pGBKT7 (TaKaRa, Dalian, China) using restriction enzymes *Eco*R1 and *Bam*H1. Recombinant plasmids were transformed into the *S. cerevisiae* Y2HGold strain (TaKaRa) and transformants were cultured on SDO and SDO/X/A plates at 30 °C for 4 days. The final concentrations of aureobasidin A and X-α-gal added to SDO/X/A were 100 ng/mL and 0.04 mg/mL, respectively. The transformants tested on SDO and SDO/X/A plates yielded white and blue colonies, respectively, indicating the tested protein/peptide had transcriptional activation activity.

### Y1H assay

One-to-one Y1H assay was used for screening TFs binding the *rsrA* promoter in accordance with the instructions provided with the Matchmaker Gold Yeast One-Hybrid System (TaKaRa). Both recombinant pAbAi and pGADT7-AD were co-transformed into Y1H Gold cells and positive transformants were selected on plates containing SD/-Leu medium with AbA (200 ng/mL). Meanwhile, the Y1H Gold strain carrying pAbAi-*cbh1* and pGADT7-*PoxCxrA* served as a positive control.

### RNA-seq analyses

Total RNA of *P. oxalicum* was sequenced on the BGI-SEQ-500 platform at BGI (Shenzhen, China). Acquired data were further analysed by BWA software version 0.7.10-r789 and Bowtie2 version 2.1.0^29^. RSEM software version 1.2.12^30^ and NOISeq tool^31^ were also employed.

### RT-qPCR assay

Total RNA of *P. oxalicum* was converted to single-stranded cDNA by HiScript III RT SuperMix for qPCR plus gDNA wiper (Vazyme Biotech Co, Ltd., Nanjing, China). Mixtures comprising cDNA used as template, primers corresponding to genes, and ChamQTM Universal SYBR qPCR Master Mix (Vazyme Biotech Co, Ltd.) were subjected to PCR amplification on a 7500 Real Time PCR System. The relative expression of each detected gene was analysed using the 2^−ΔΔCT^ method^32^.

### Construction of *P. oxalicum* mutants

DNA fragments were obtained via PCR amplification with specific primers (Supplementary Table S2) and purified using DNA purification Kit (Tiangen Biotech Co., Ltd., Beijing, China). The knockout cassette was generated by fusion PCR and introduced into *P. oxalicum* protoplasts by the PEG chemical method^19^. Transformants were screened using the antibiotic bleomycin (100 μg/mL) and/or G418 (800 μg/mL), then verified by PCR with specific primers (Supplementary Table S2).

### Measurement of raw starch-degrading enzyme and soluble starch-degrading enzyme activity

Activities of raw starch-degrading enzyme and soluble starch-degrading enzyme of culture supernatant from *P*. *oxalicum* were tested using the 3,5-dinitrosalicylic acid method^33^. Briefly, raw cassava flour from a farmer’s market in Nanning (China) and commercial starch of corn (Sigma-Aldrich) was used for substrates to determine raw starch-degrading enzyme and soluble starch-degrading enzyme activities, respectively. Culture supernatant of 50 μL was mixed with the citrate phosphate buffer of 450 μL (pH 4.5) containing 1% (w/v) substrate, and the mixture reacted at 65 °C and 55 °C for 30 min, respectively. The inactivated crude enzyme was used as control. The released reducing sugar was measured with 3,5-dinitrosalicylic acid. One unit (U) of RSDE and SSDE activity was defined as the amount of enzyme that produced 1 μmol of reducing sugar per min from the appropriate substrate. The production of RSDE and SSDE was recorded as U/gram of dry mycelial weight.

### Determination of *P. oxalicum* biomass

Fresh asexual spores (1 × 10^8^) of *P. oxalicum* were inoculated into liquid media containing glucose and commercial starch of corn, then cultivated for 3 days at 28 °C with shaking at 180 rpm. The resulting mycelia were collected every 12 h, dried at 50 °C, and weighted.

### Observation of *P. oxalicum* colony phenotype and mycelial development

Fresh asexual spores (1×10^8^) of *P. oxalicum* were inoculated onto solid plates containing PDA, glucose and SCS, and cultivated at 28 °C for between 48 h and 5 days. A Canon EOS 6D camera (Canon Inc., Tokyo, Japan) and an Olympus DP480 light microscope (Olympus, Tokyo, Japan) were respectively used to take photographs of *P. oxalicum* colony phenotype and mycelial development. Images were analysed by cellSence Dimension digital imaging software (Olympus).

### Heterologous expression of recombinant polypeptides and in vitro EMSA

Target DNA fragments were amplified by PCR with corresponding primer pairs (Supplementary Table S2) and products were cloned into plasmid pET-32a(+) to generate recombinant plasmids. Recombinant plasmids were introduced into freshly competent *Escherichia coli* Rossetta cells (TaKaRa) to obtain positive transformants which were screened using kanamycin antibiotic (50 µg/mL). *E. coli* cells were cultured in Luria-Bertani medium for 5 h at 30 °C, 1 mM isopropyl-β-d-thiogalactopyranoside (Solarbio Life Sciences, Beijing, China) was added to induce target protein expression, and culture was continued for 24 h at 16 °C. *E. coli* Rossetta cells were collected and disrupted to extract recombinant polypeptides, which were purified using ProteinIso Ni-NTA Resin (TransGen, Shanghai, China).

The procedure for in vitro EMSA was performed as described previously^19^. Probes of detected genes were amplified by PCR using corresponding primer pairs (Supplementary Table S2) and mixed with recombinant polypeptides for 20 min at 28 °C. Mixtures were passed through non-denatured polyacrylamide adhesive and band shifts were recorded using a Bio-Rad ChemiDoc MP Imaging System (Bio-Rad Laboratories, Hercules, CA, USA).

### Bioinformatics software

Homologous alignment and evolution analyses were performed using MUSCLE and MEGA version X^32^, respectively.

### Statistics and reproducibility

Statistical analysis of experimental data was performed using Microsoft Excel (Office 2019, Microsoft, Redmond, WA) and SPSS (IBM, Armonk, NY) with Student’s t test and one-way analysis of variance (ANOVA). Results were generated from at least three independent experiments and reproducibility was confirmed. Data values indicate mean ± standard deviation, where *N* equals the number of independent experiments.

### Reporting summary

Further information on research design is available in the Nature Portfolio Reporting Summary linked to this article.

### Supplementary information

Supplementary material

Description of Additional Supplementary Materials

Supplementary Data 1

Supplementary Data 2

Supplementary Data 3

Supplementary Data 4

Reporting Summary

## Data Availability

All data for gene sequences could be found in the submitted genome of *P. oxalicum* strain HP7-1 in GenBank (accession number JRVD02000000). Transcriptomic data for *P*. *oxalicum* strains have been deposited in the Sequence Read Archive database (Accession No. GSE245046 and GSE248520). All uncropped and unedited blots/gels were found in Supplementary Fig. S16. The numerical source data behind the graphs can be found in Supplementary Data 4.
