# Supplementary material for: A RsrC-RsrA-RsrB transcriptional circuit positively regulates polysaccharide-degrading enzyme biosynthesis and development in Penicillium oxalicum
Source: Commun Biol. 2024 Jul 11;7:848. doi: 10.1038/s42003-024-06536-4 (PMC11239660; doi:10.1038/s42003-024-06536-4)
Supplement: Supplementary file 1 — Supplementary material [file 42003_2024_6536_MOESM1_ESM.pdf]

Supplementary Information

A RsrC-RsrA-RsrB transcriptional circuit positively regulates polysaccharide-degrading enzyme biosynthesis and development in *Penicillium oxalicum*

Yuan-Ni Ning[1,2,3#], Xue Liang[1,2,3#], Xin Shen[1,2,3], Di Tian[1,2,3], Wen-Tong Li[1,2,3], Xue-Mei Luo[3], Jia-Xun Feng[1,2,3*], Shuai Zhao[1,2,3*]

[1]State Key Laboratory for Conservation and Utilization of Subtropical Agro-bioresources, Guangxi University, 100 Daxue Road, Nanning, Guangxi 530004, People's Republic of China.

[2]Guangxi Research Center for Microbial and Enzyme Engineering Technology, Guangxi University, 100 Daxue Road, Nanning, Guangxi 530004, People's Republic of China.

[3]College of Life Science and Technology, Guangxi University, 100 Daxue Road, Nanning, Guangxi 530004, People's Republic of China.

Content:

Supplementary Figures 1–15 and legends

Supplementary Table S1

[#] These authors contributed equally to this work.

***Correspondence:** shuaizhao0227@gxu.edu.cn; jiaxunfeng@sohu.com

Tel: +86-771-323-9401

Mailing address: College of Life Science and Technology, Guangxi University, 100 Daxue Road, Nanning, Guangxi 530004, People's Republic of China

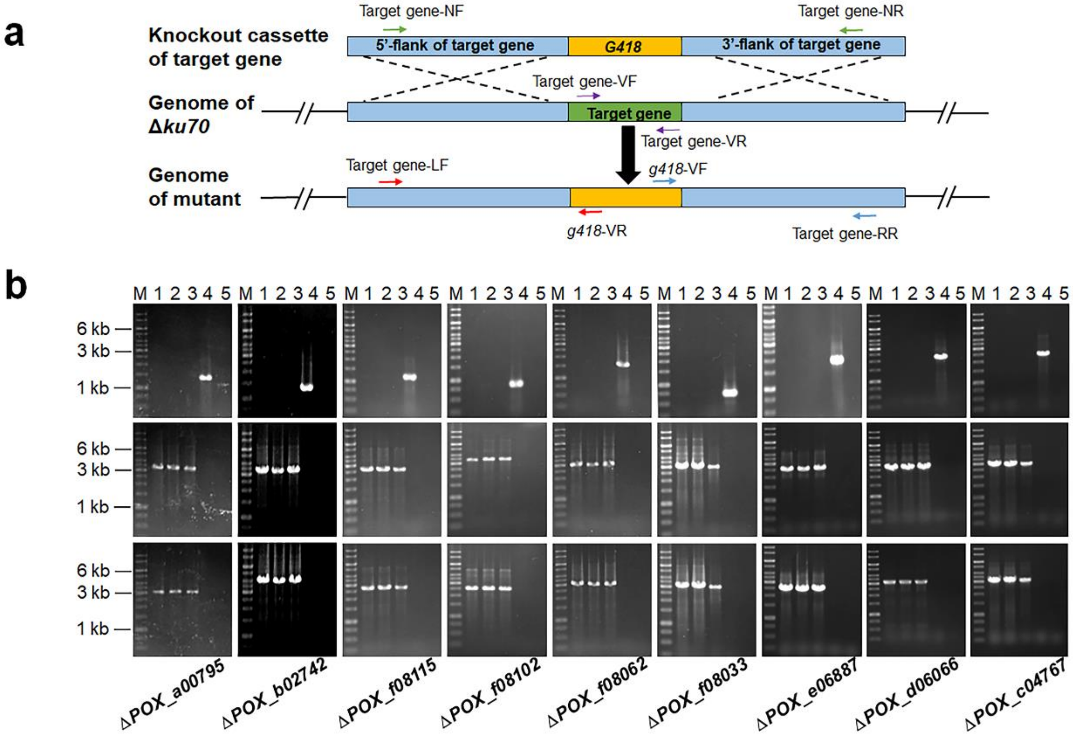

Supplementary Fig. S1 Construction strategy (a) and PCR confirmation (b) of deletion mutants of candidate regulatory genes acting downstream of RsrA. In panel b, M = 1 kb markers; 1–3 = three transformants; 4 = Δ*ku70*; 5 = ddH_2O. Panels from top to bottom indicate PCR production of target genes, left-cross fragments, and right-cross fragments. *G418*, geneticin resistance gene.

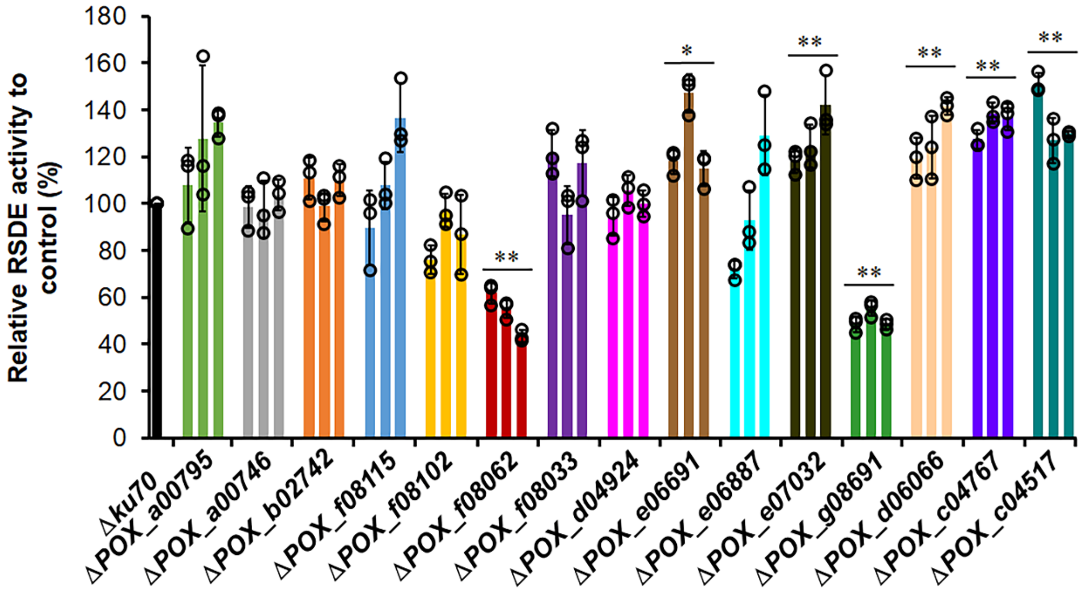

Supplementary Fig. S2 RSDE production on soluble corn starch. All *P. oxalicum* strains were cultured in medium containing SCS for 6 days. RSDE production by each mutant was normalised to that in parental strain Δ*ku70*; **p* <0.05 and ***p* <0.01 indicate significant differences between mutants and parental strain, assessed by Student's *t*-test. RSDE, raw starch-degrading enzyme.

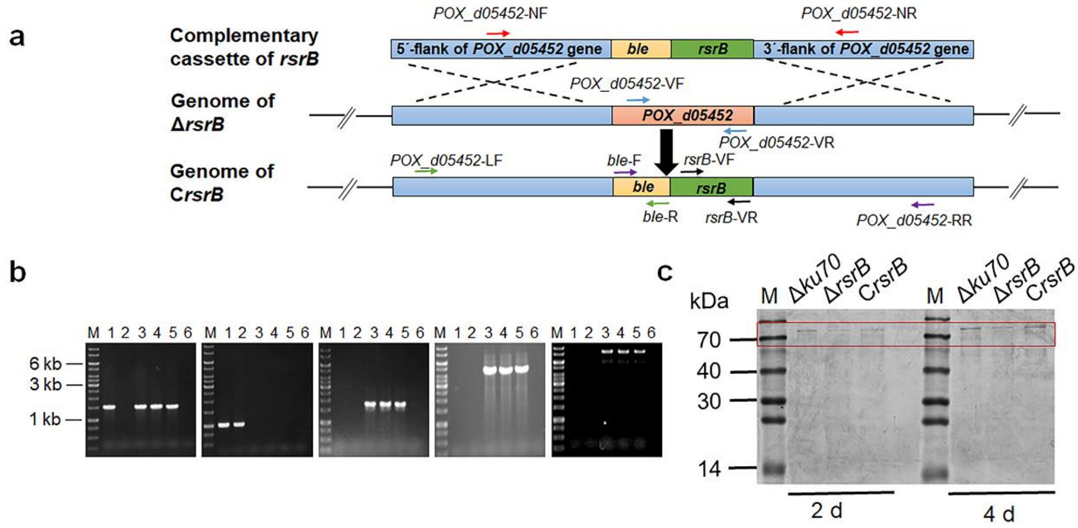

Supplementary Fig. S3 Construction strategy (a) and PCR verification (b) of complementation strain C*rsrB*, and SDS-PAGE analysis of extracellular proteins (c). In panel b, M: 1 kb markers; 1: Δ*ku70*; 2: Δ*rsrB*; 3: C*rsrB*-1; 4: C*rsrB*-2; 5: C*rsrB*-3; 6: ddH_2O. Panels from left to right are PCR production of *rsrB*, *POX_d05452*, *ble*, left-cross fragments and right-cross fragments. *ble*, bleomycin resistance gene. In panel c, SDS-PAGE analysis of extracellular proteins of the supernatant of culture by *P*. *oxalicum* mutant Δ*rsrB*, parental strain Δ*ku70* and complementation strain C*rsrB* in the presence of soluble corn starch (SCS). Red rectangle indicates target proteins of the right sizes of major amylases including PoxGA15A and PoxAmy13A. Each lane contained supernatant of culture 24 μL. M: 120 kDa protein Marker.

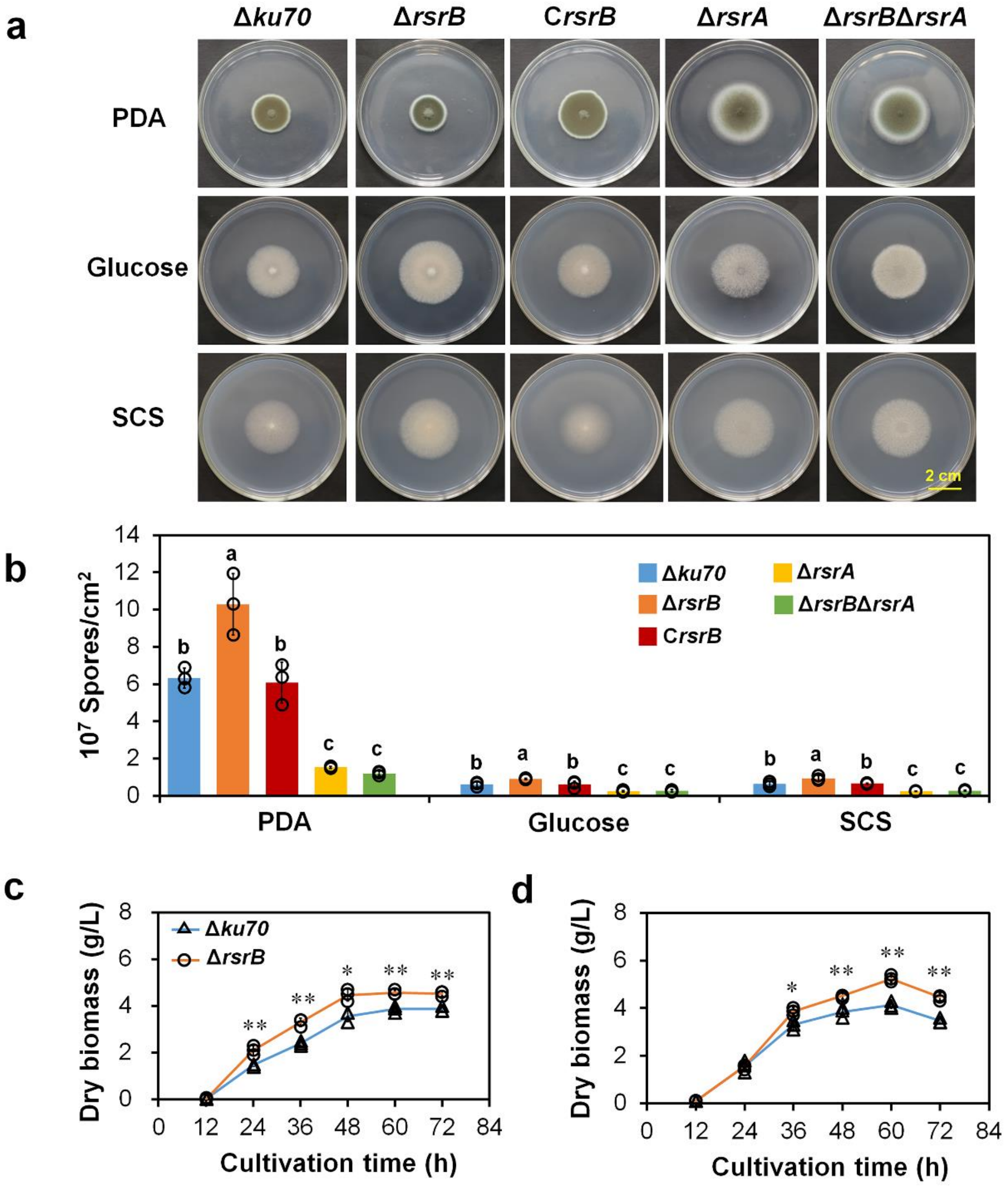

Supplementary Fig. S4 Effects of RsrB on the phenotype and sporulation of *P. oxalicum*. (a) Colony observation. (b) Production of asexual spores. All tested strains were cultured on potato dextrose agar (PDA), glucose and SCS plates for 5 days. Wild-type strain Δ*ku70*, complementation strain C*rsrB* and mutant Δ*rsrA* served as controls. Lowercase letters represent p <0.05, respectively. Different letters indicate significant differences, evaluated by one-way ANOVA. (c) Mycelial growth in media containing glucose or SCS (d).

Mutant Δ*rsrB* and control strain C*rsrB* were directly inoculated into liquid medium and cultured for 12–72 h. Results are means ± standard deviation (SD). $^{**}p < 0.01$ and $^{*}p < 0.05$ (Student's *t*-test) represent significant differences between mutant Δ*rsrB* and Δ*ku70*.

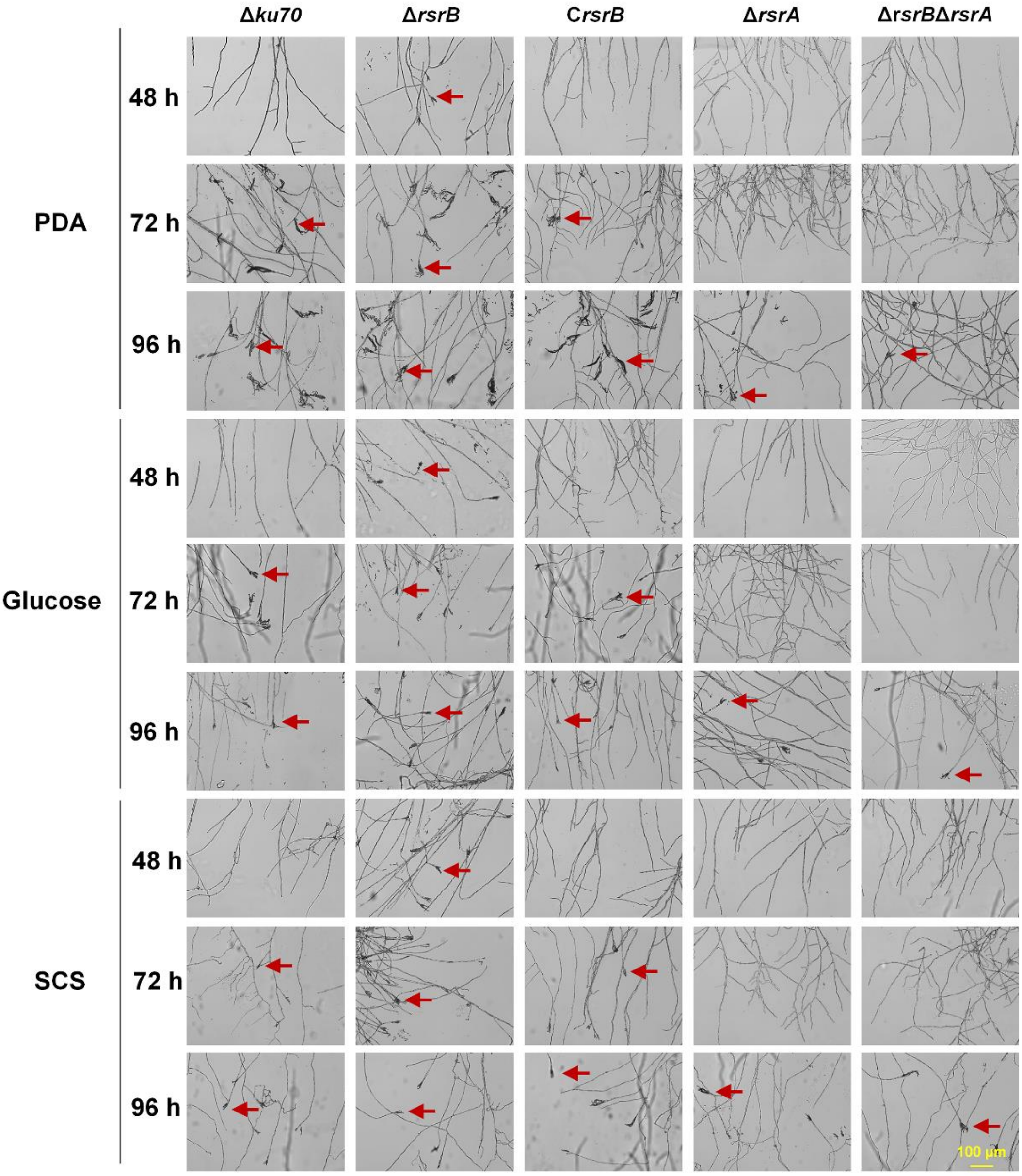

Supplementary Fig. S5 Mycelial observation of *P. oxalicum* mutants. All strains were grown on solid plates containing PDA, glucose and SCS for 48 h, 72 h and 96 h. Red arrows indicate conidiospores.

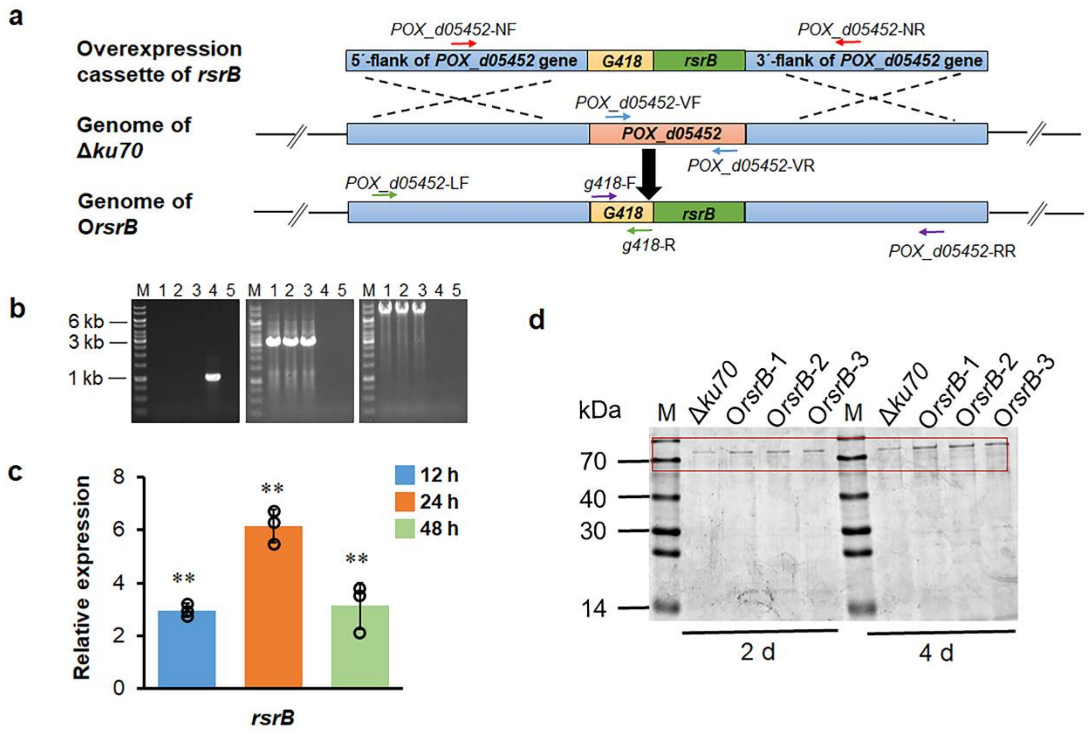

Supplementary Fig. S6 Construction strategy (a) and PCR confirmation (b) of overexpression strain O*rsrB*, and transcription levels of *rsrB* in O*rsrB* (c), and SDS-PAGE analysis of extracellular proteins (d). In panel b, M: 1 kb markers; 1: O*rsrB*-1; 2: O*rsrB*-2; 3: O*rsrB*-3; 4: Δ*ku70*; 5: ddH_2O. Panels from left to right are PCR production of *POX_d05452*, left-cross fragments and right-cross fragments. *G418*, geneticin resistance gene. In panel c, transcription levels of *rsrB* in overexpression strain O*rsrB*. *P*. *oxalicum* strains were cultured in glucose medium for 24 h, then transferred into medium containing SCS and cultivated for 12–48 h. Expression levels of the tested genes in mutants were normalised against those in wild-type strain Δ*ku70*. $^{**}p \leq 0.01$ indicates significant differences between mutant and Δ*ku70* strains. Each experiment was repeated three times. In panel d, SDS-PAGE analysis of extracellular proteins of the supernatant of culture by *P*. *oxalicum* overexpression strain

O*rsrB* and parental strain Δ*ku70* in the presence of soluble corn starch (SCS). Red rectangle indicates target proteins of the right sizes of major amylases including PoxGA15A and PoxAmy13A. Each lane contained supernatant of culture 24 μL. M: 120 kDa protein Marker.

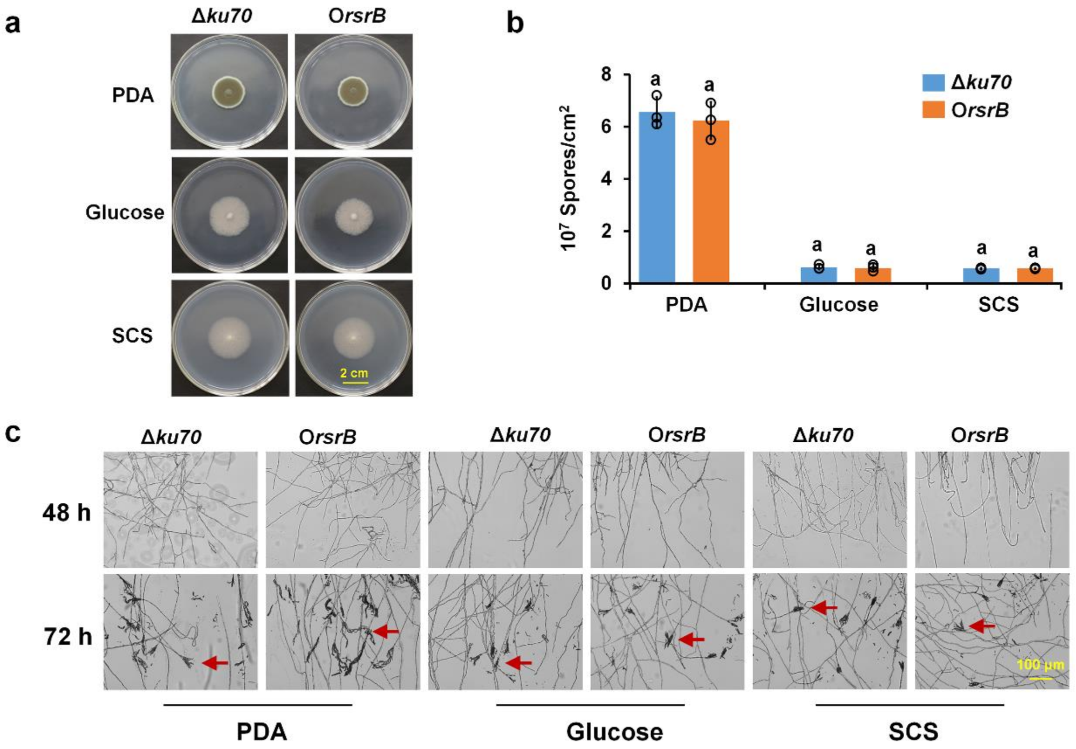

Supplementary Fig. S7 Colony observation (a), production of asexual spores (b) and mycelial observation (c) of *P. oxalicum* mutants O*rsrB*. In panels a and b, all tested strains were cultured on PDA, glucose or SCS solid plates for 5 days. Wild-type strain Δ*ku70* served as a control. In panel b, lowercase letters represent p <0.05, respectively. Different letters indicate significant differences, evaluated by one-way ANOVA. In panel c, all strains were grown on solid plates containing PDA, glucose or SCS for 48 h and 72 h. Wild-type strain Δ*ku70* served as a control. Red arrows indicate conidiospores.

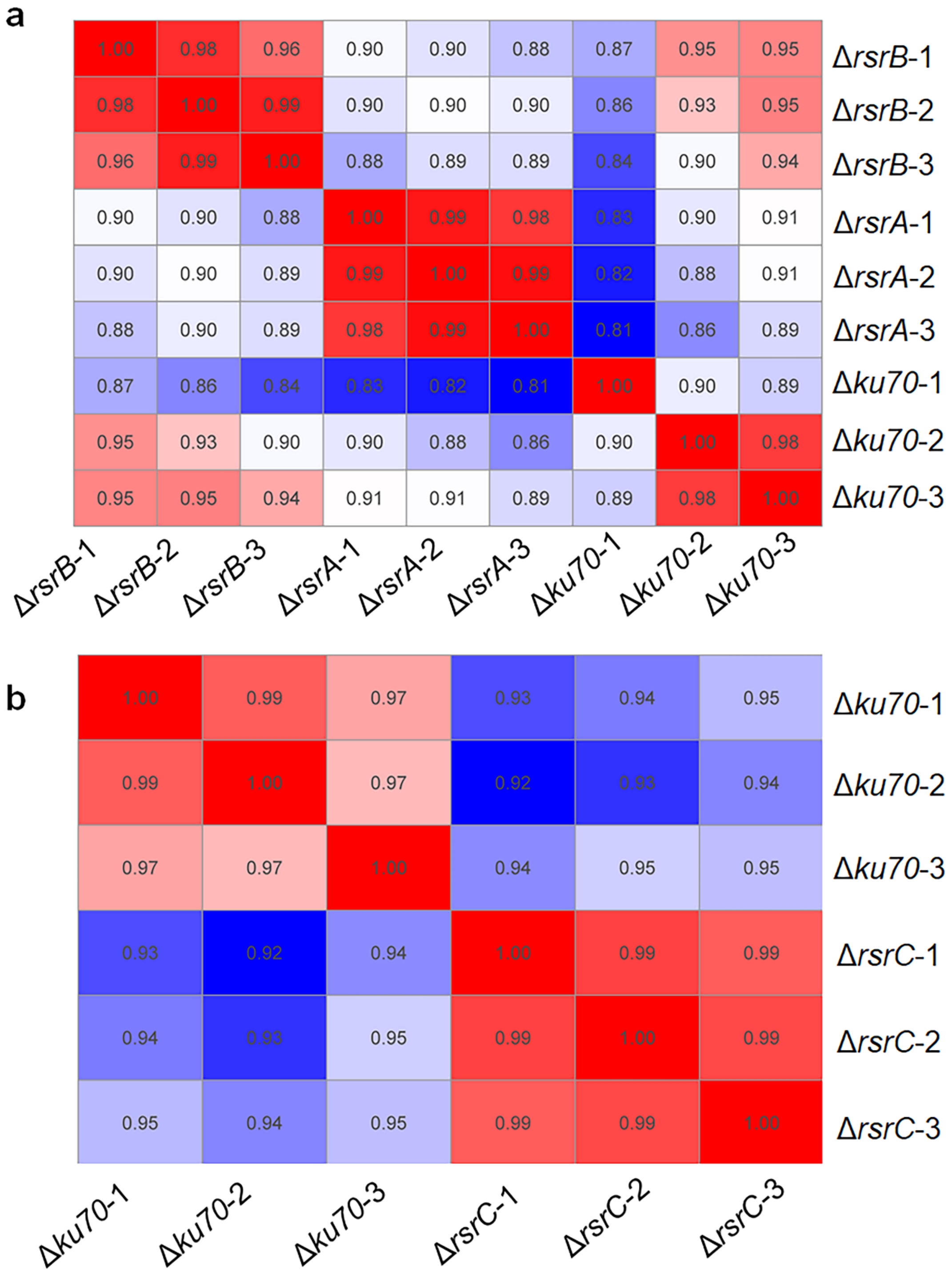

Supplementary Fig. S8 Pearson's correlation heatmap of Δ*rsrB* and Δ*rsrA* (a), and Δ*rsrC* (b) transcriptomes compared with that of controls strain Δ*ku70*. Total RNA was extracted from the mycelia of each strain after culture on SCS for 24 h, following a transfer from glucose.

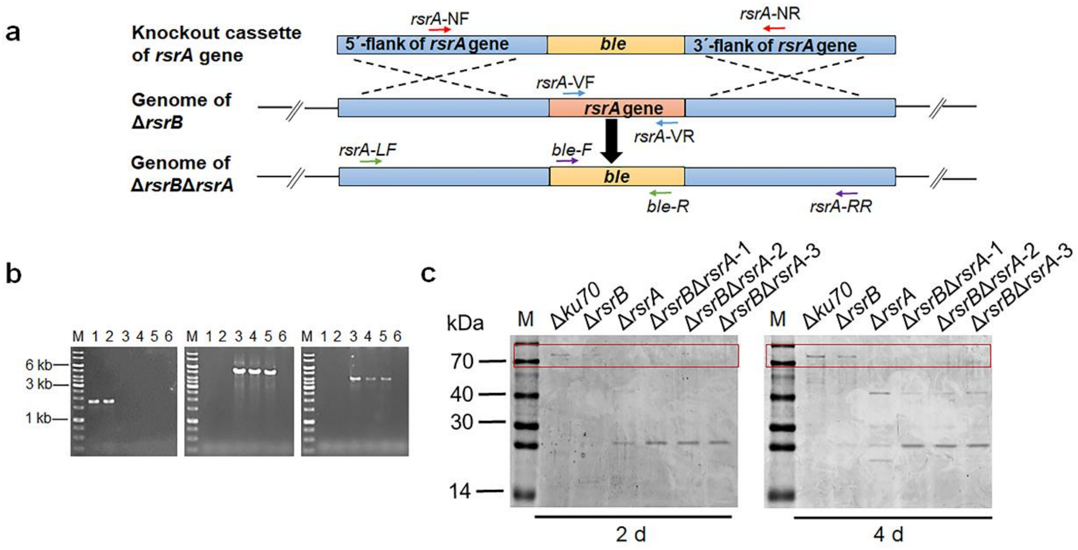

Supplementary Fig. S9 Construction (a) and PCR verification (b) of double deletion strain Δ*rsrB*Δ*rsrA*, and SDS-PAGE analysis of extracellular proteins (c). In panel b, M: 1 kb markers; 1: Δ*ku70*; 2: Δ*rsrB*; 3: Δ*rsrB*Δ*rsrA*-1; 4: Δ*rsrB*Δ*rsrA*-2; 5: Δ*rsrB*Δ*rsrA*-3; 6: ddH_2O. Panels from left to right are PCR production of *rsrA*, left-cross fragments and right-cross fragments. *ble*, bleomycin resistance gene. In panel c, SDS-PAGE analysis of extracellular proteins of the supernatant of culture by *P*. *oxalicum* double deletion strain Δ*rsrB*Δ*rsrA*, parental strain Δ*ku70*, mutants Δ*rsrB* and Δ*rsrA* in the presence of soluble corn starch (SCS). Red rectangles indicate target proteins of the right sizes of major amylases including PoxGA15A and PoxAmy13A. Each lane contained supernatant of culture 24 μL. M: 120 kDa protein Marker.

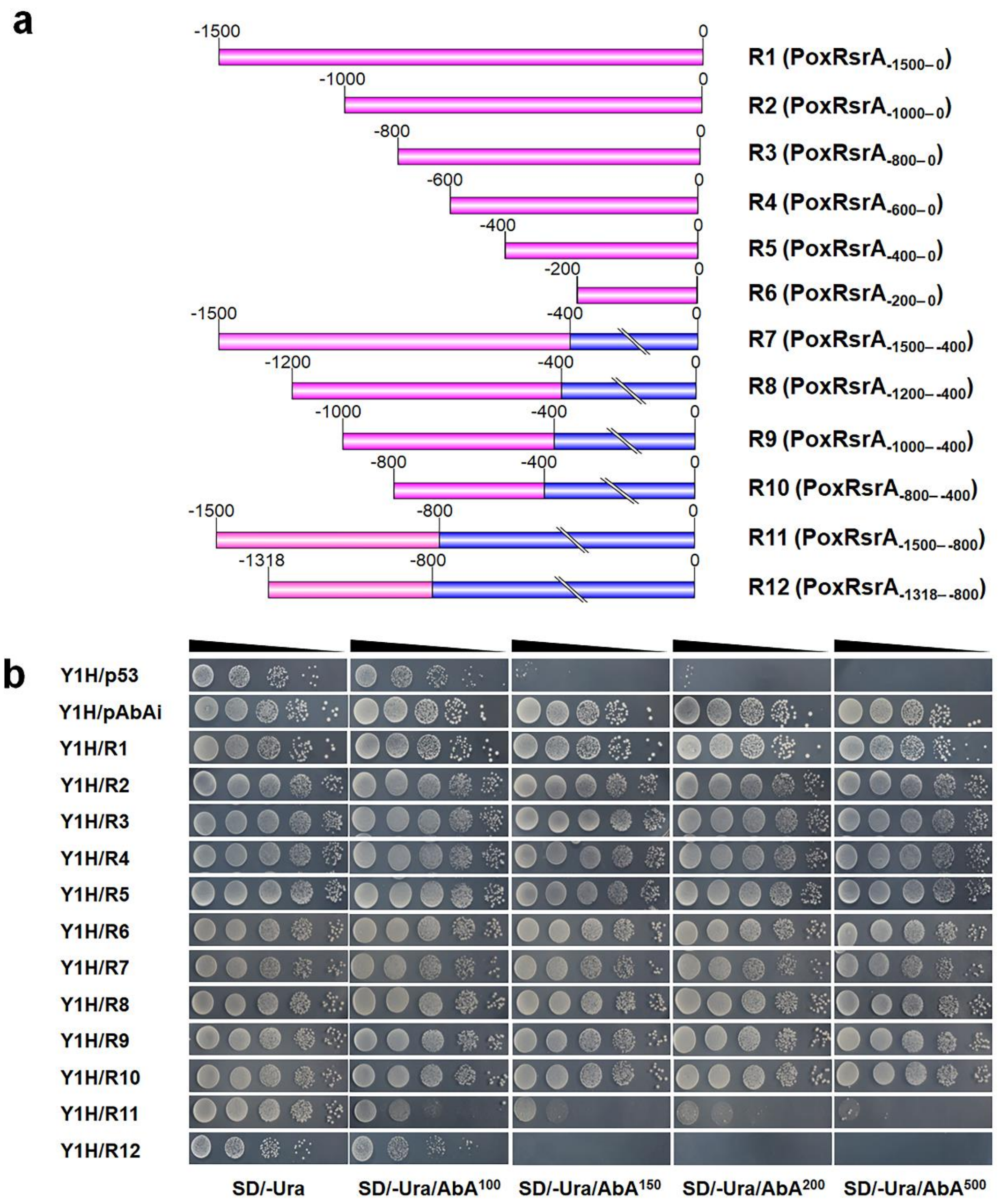

Supplementary Fig. S10 Y1H assay of different length DNA fragments upstream of *rsrA* self-activation. (a) Different length DNA fragments of *rsrA* upstream. (b) Recombinant Y1HGold cells cultured on (SD)/-Ura and different concentrations (0, 100, 150, 200 and 500 ng/mL) of aureobasidin A (AbA) incubated at 30°C for 3 days.

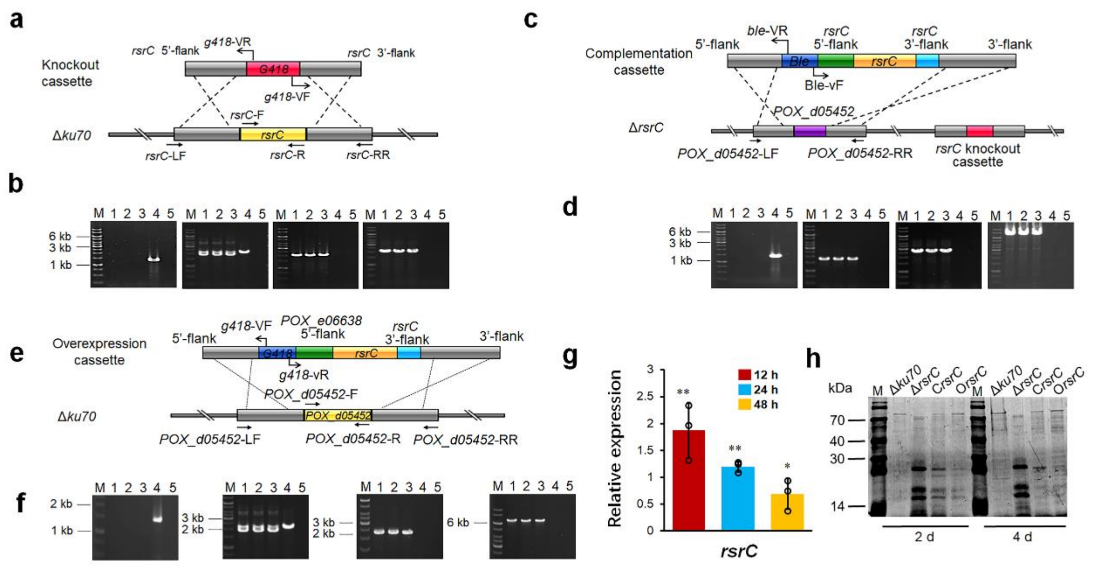

Supplementary Fig. S11 Construction strategy and PCR verification of mutant Δ*rsrC*, complementation strain C*rsrC* and overexpression strain O*rsrC*, and transcription levels of *rsrC* in O*rsrC*, and SDS-PAGE analysis of extracellular proteins (h). (a) Construction strategy of Δ*rsrC*. (b) PCR verification of Δ*rsrC*. M: 1 kb marker; 1: Δ*rsrC*-1; 2: Δ*rsrC*-2; 3: Δ*rsrC*-3; 4: Δ*ku70*; 5: ddH_2O. Panels from left to right are PCR production of *rsrC*, *g418*, left-cross fragments and right-cross fragments. (c) Construction strategy of C*rsrC*. (d) PCR verification of C*rsrC*. M: 1 kb marker; 1: C*rsrC*-1; 2: C*rsrC*-2; 3: C*rsrC*-3; 4: Δ*rsrC*; 5: ddH_2O. Panels from left to right were PCR production of *POX_d05452*, *rsrC*, left-cross fragments and right-cross fragments. (e) Construction strategy of O*rsrC*. (f) PCR verification of O*rsrC*. M: 1 kb markers; 1: O*rsrC*-1; 2: O*rsrC*-2; 3: O*rsrC*-3; 4: Δ*ku70*; 5: ddH_2O. Panels from left to right are PCR production of *POX_d05452*, *g418*, left-cross fragments and right-cross fragments. (g) Transcription levels of *rsrC* in overexpression strain O*rsrC*. *P. oxalicum* strains were cultured in glucose medium for 24 h, then transferred into

medium containing SCS and cultivated for 12–48 h. Expression levels of the tested genes in mutants were normalised against those in wild-type strain Δ*ku70*. ***p* ≤0.01 and **p* ≤0.05 indicate significant differences between mutant and Δ*ku70* strains. Each experiment was repeated three times. *ble*, bleomycin resistance gene*. G418*, geneticin resistance gene. In panel h, SDS-PAGE analysis of extracellular proteins of the supernatant of culture by *P*. *oxalicum* mutant Δ*rsrC* and overexpression strain O*rsrC* in the presence of soluble corn starch (SCS). Red rectangle indicates target proteins of the right sizes of major amylases including PoxGA15A and PoxAmy13A. Each lane contained supernatant of culture 24 μL. M: 120 kDa protein Marker.

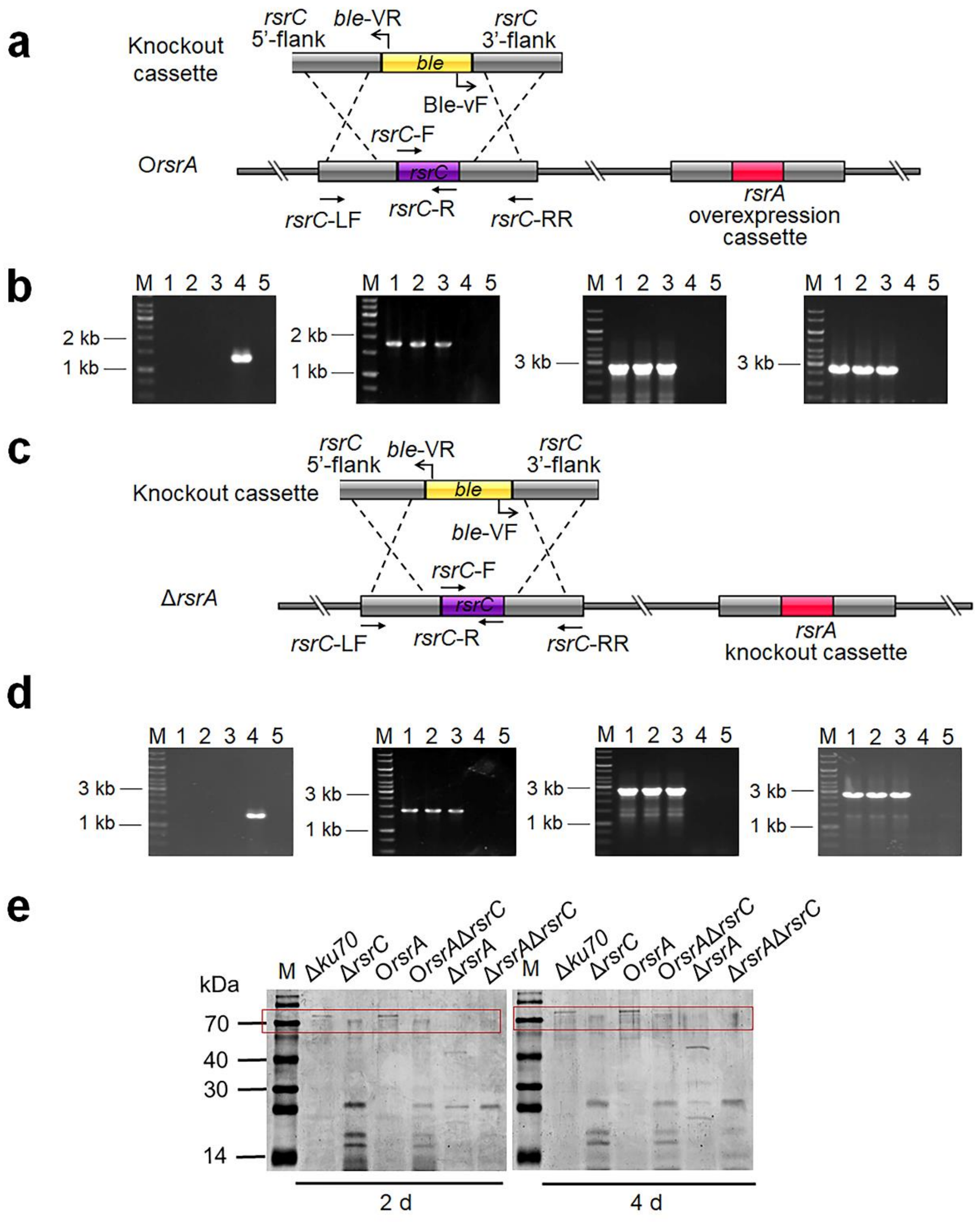

Supplementary Fig. S12 Construction strategy and PCR verification of mutants O*rsrA*Δ*rsrC* and Δ*rsrA*Δ*rsrC*, and SDS-PAGE analysis of extracellular proteins (e). (a) Construction strategy of O*rsrA*Δ*rsrC*. (b) PCR verification of O*rsrA*Δ*rsrC*. M: 1 kb marker; 1: O*rsrA*Δ*rsrC*-1; 2: O*rsrA*Δ*rsrC*-2; 3: O*rsrA*Δ*rsrC*-3; 4: Δ*ku70*; 5: ddH_2O. Panels from left to right are PCR production of *rsrC*, *ble*, left-cross fragments and right-cross fragments. (c)

Construction strategy of Δ*rsrA*Δ*rsrC*. (d) PCR verification of Δ*rsrA*Δ*rsrC*. M: 1 kb marker; 1: Δ*rsrA*Δ*rsrC*-1; 2: Δ*rsrA*Δ*rsrC*-2; 3: Δ*rsrA*Δ*rsrC*-3; 4: Δ*ku70*; 5: ddH_2O. Panels from left to right are PCR production of *rsrC*, *ble*, left-cross fragments and right-cross fragments. *ble*, bleomycin resistance gene. In panel e, SDS-PAGE analysis of extracellular proteins of the supernatant of culture by *P*. *oxalicum* mutant O*rsrA*Δ*rsrC* and Δ*rsrA*Δ*rsrC* in the presence of soluble corn starch (SCS). Red rectangles indicate target proteins of the right sizes of major amylases including PoxGA15A and PoxAmy13A. Each lane contained supernatant of culture 24 μL. M:120 kDa protein Marker.

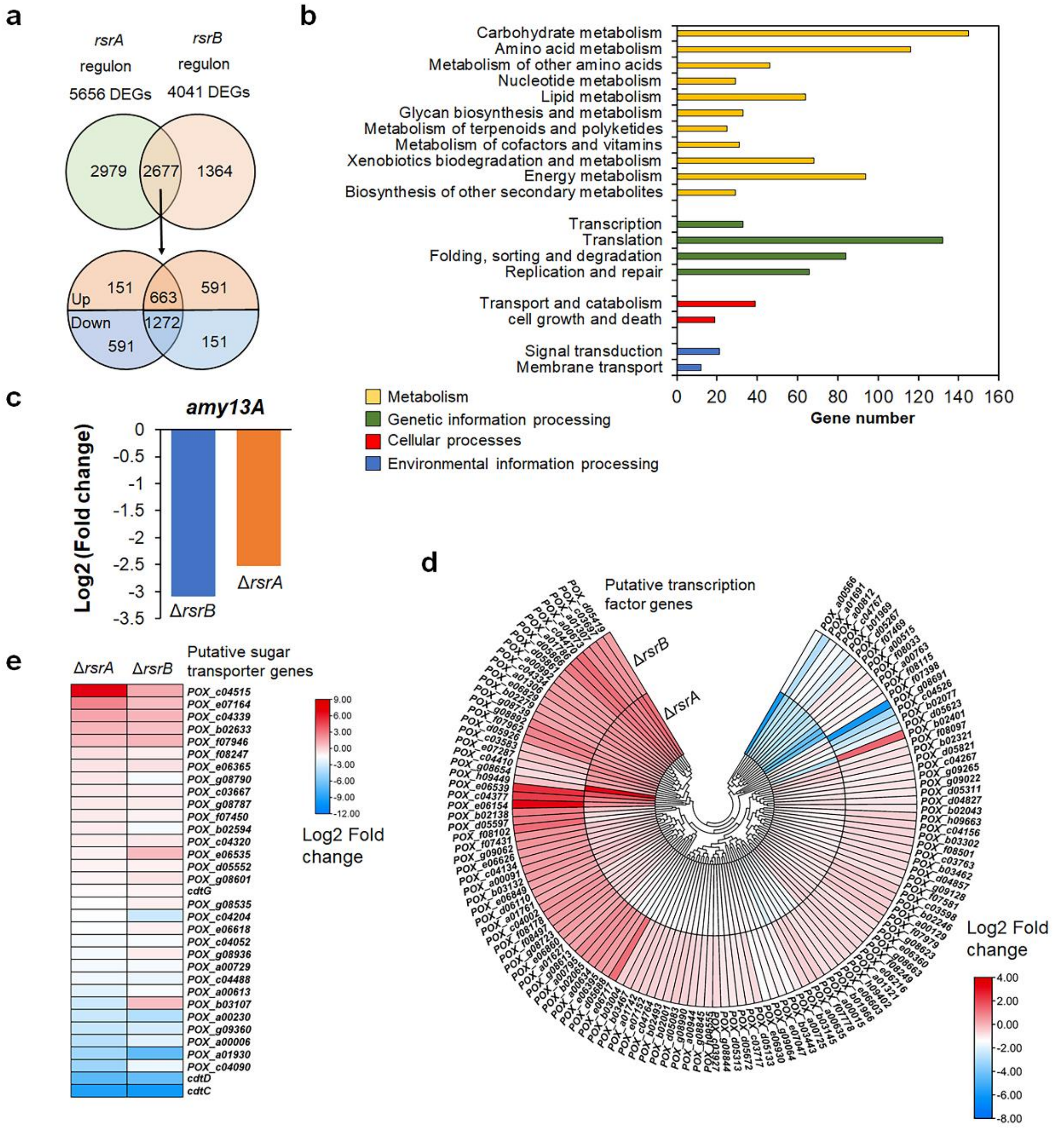

Supplementary Fig. S13 Co-regulation between RsrA and RsrB in *P. oxalicum*. (a) Number of DEGs between RsrB and RsrA regulons. ‘Up’ and ‘Down’ indicate up- and downregulation. (b) KEGG annotations of genes co-regulated by RsrB and RsrA. (c) Expression of α-amylase gene *amy13A* in mutants Δ*rsrB* and Δ*rsrA* compared with Δ*ku70*. (d) Heatmap showing the expression of DEGs encoding putative transcription factors. (e) Expression of DEGs encoding sugar transporters.

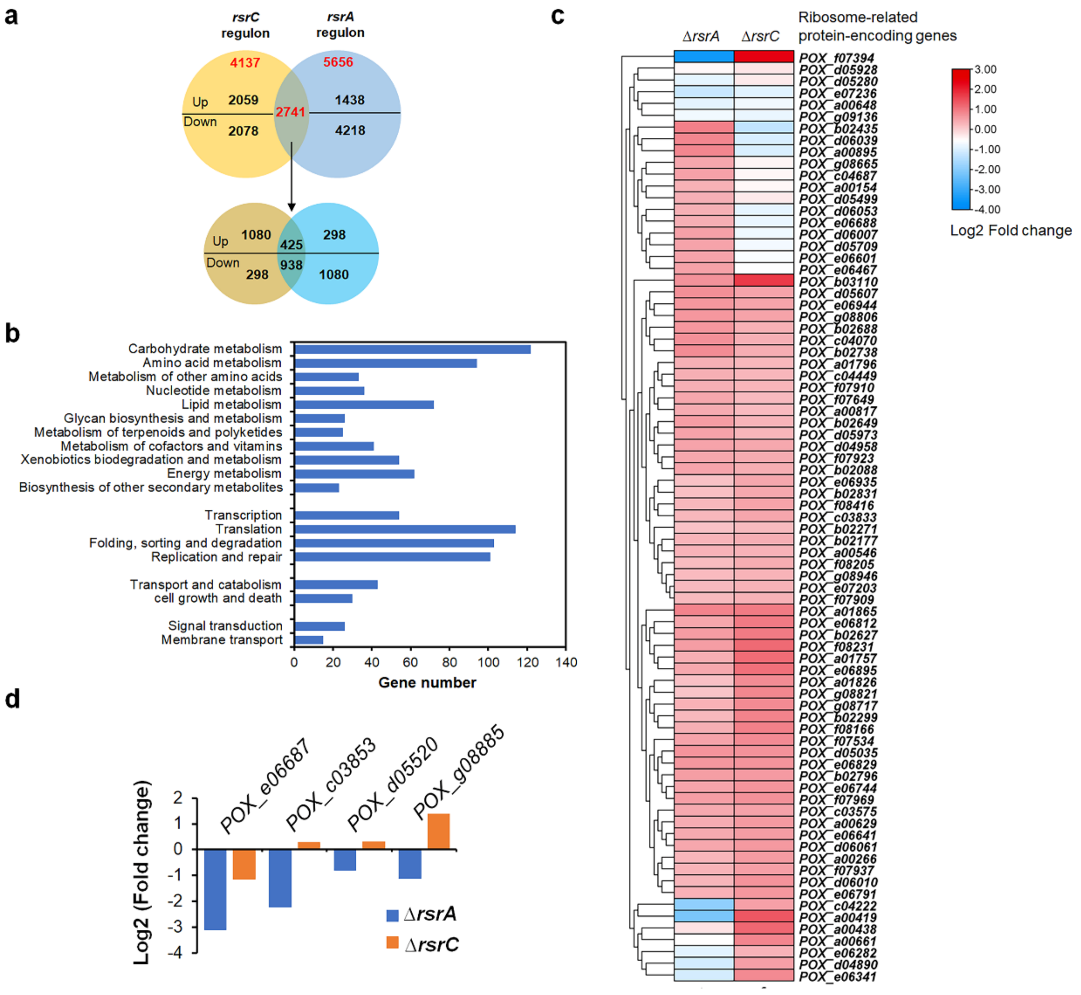

Supplementary Fig. S14 Co-regulation between RsrA and RsrC in *P. oxalicum*. (a) Number of DEGs between RsrC and RsrA regulons. ‘Up’ and ‘Down’ indicate up- and downregulation. (b) KEGG annotations of genes co-regulated by RsrC and RsrA. (c) Heatmap showing the expression of DEGs involved in ribosome biogenesis and assembly in mutants Δ*rsrA* and Δ*rsrC* compared with Δ*ku70*. (d) Expression of amylase genes in mutants Δ*rsrA* and Δ*rsrC*.

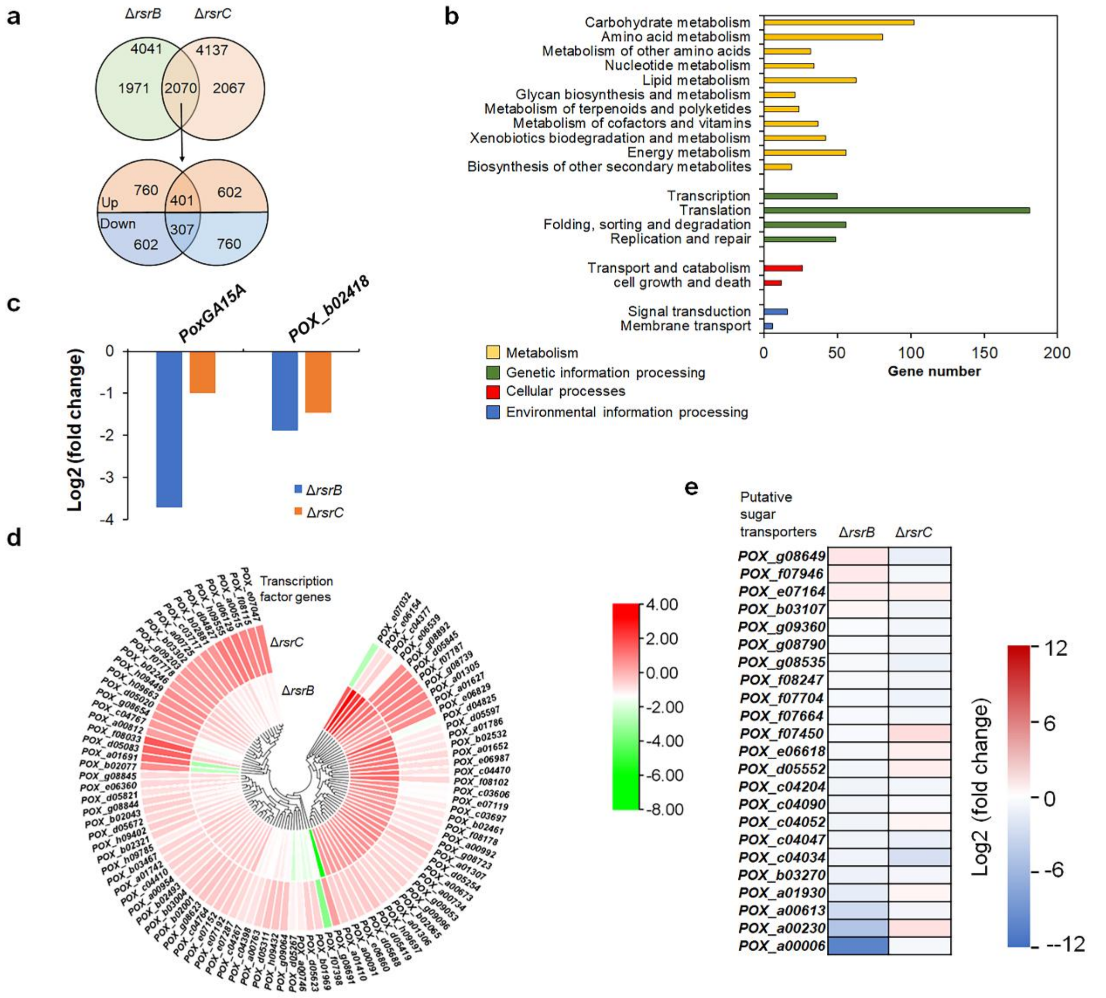

Supplementary Fig. S15 Co-regulation between RsrB and RsrC in *P. oxalicum*. (a) Number of DEGs between RsrB and RsrC regulons. ‘Up’ and ‘Down indicate up- and downregulation. (b) KEGG annotations of genes co-regulated by RsrB and RsrC. (c) Expression of amylase genes *PoxGA15A* and *POX_b02418* in mutants Δ*rsrB* and Δ*rsrC* compared with Δ*ku70*. (d) Heatmap showing the expression of DEGs encoding putative transcription factors in mutants Δ*rsrB* and Δ*rsrC* compared with Δ*ku70*. (e) Expression of DEGs encoding sugar transporters in mutants Δ*rsrB* and Δ*rsrC* compared with Δ*ku70*.

Supplementary Table S1 *Penicillium oxalicum* strains used in this study

Strain	Description	Genotypes	Reference
Δ*ku70*	Deletion strain of *ku70*	*Ku70*$^{-}$; *Hph*$^{R+}$	1
Δ*rsrA*	Deletion strain of *rsrA*	*Ku70*$^{-}$; *rsrA*$^{-}$; *Hph*$^{R+}$; G418^{R+}	2
C*rsrA*	Complementation strain of Δ*rsrA*	*Ku70*$^{-}$; *Hph*$^{R+}$; G418^{R+}; *Ble*$^{R+}$	2
O*rsrA*	Overexpression strain of gene *rsrA*	*Ku70–; rsrA++; HphR+; G418R+*	3
Δ*POX_a00795*	Deletion strain of *POX_a00795*	*Ku70*$^{-}$; *POX_a00795*$^{-}$; *Hph*$^{R+}$; *G418*$^{R+}$	This study
Δ*POX_a00746*	Deletion strain of *POX_a00746*	*Ku70*$^{-}$; *POX_a00746*$^{-}$; *Hph*$^{R+}$; *G418*$^{R+}$	This study
Δ*POX_b02742*	Deletion strain of *POX_b02742*	*Ku70*$^{-}$; *POX_b02742*$^{-}$; *Hph*$^{R+}$; *G418*$^{R+}$	This study
Δ*POX_f08115*	Deletion strain of *POX_f08115*	*Ku70*$^{-}$; *POX_f08115*$^{-}$; *Hph*$^{R+}$; *G418*$^{R+}$	This study
Δ*POX_f08102*	Deletion strain of *POX_f08102*	*Ku70*$^{-}$; *POX_f08102*$^{-}$; *Hph*$^{R+}$; *G418*$^{R+}$	This study
Δ*POX_f08062*	Deletion strain of *POX_f08062*	*Ku70*$^{-}$; *POX_f08062*$^{-}$; *Hph*$^{R+}$; *G418*$^{R+}$	This study
Δ*POX_f08033*	Deletion strain of *POX_f08033*	*Ku70*$^{-}$; *POX_f08033*$^{-}$; *Hph*$^{R+}$; *G418*$^{R+}$	This study
Δ*POX_d04924*	Deletion strain of *POX_d04924*	*Ku70*$^{-}$; *POX_d04924*$^{-}$; *Hph*$^{R+}$; *G418*$^{R+}$	This study
Δ*POX_e06691*	Deletion strain of *POX_e06691*	*Ku70*$^{-}$; *POX_e06691*$^{-}$; *Hph*$^{R+}$; *G418*$^{R+}$	This study
Δ*POX_e06887*	Deletion strain of *POX_e06887*	*Ku70*$^{-}$; *POX_e06887*$^{-}$; *Hph*$^{R+}$; *G418*$^{R+}$	This study
Δ*POX_e07032*	Deletion strain of *POX_e07032*	*Ku70*$^{-}$; *POX_e07032*$^{-}$; *Hph*$^{R+}$; *G418*$^{R+}$	This study
Δ*POX_d06066*	Deletion strain of *POX_d06066*	*Ku70*$^{-}$; *POX_d06066*$^{-}$; *Hph*$^{R+}$; *G418*$^{R+}$	This study
Δ*POX_c04767*	Deletion strain of *POX_c04767*	*Ku70*$^{-}$; *POX_c04767*$^{-}$; *Hph*$^{R+}$; *G418*$^{R+}$	This study
Δ*POX_c04517*	Deletion strain of *POX_c04517*	*Ku70*$^{-}$; *POX_c04517*$^{-}$; *Hph*$^{R+}$; *G418*$^{R+}$	This study
Δ*POX_g08691*	Deletion strain of *POX_g08691*	*Ku70*$^{-}$; *POX_g08691*$^{-}$; *Hph*$^{R+}$; *G418*$^{R+}$	This study
C*POX_g08691*	Complementation strain of Δ*POX_g08691*	*Ku70*$^{-}$; *Hph*$^{R+}$; *G418*$^{R+}$; *Ble*$^{R+}$	This study
Δ*rsrb*Δ*rsrA*	Mutant where *rsrB* and *rsrA* are deleted	*Ku70*$^{-}$; *rsrB*$^{-}$; *rsrA*$^{-}$; *Hph*$^{R+}$; *G418*$^{R+}$; *Ble*$^{R+}$	This study
O*rsrB*	Overexpression strain of gene *rsrB*	*Ku70–; rsrB++; HphR+; G418R+*	This study
Δ*rsrC*	Deletion strain of *rsrC*	*Ku70*$^{-}$; *rsrC*$^{-}$; *Hph*$^{R+}$; G418^{R+}	This study

C*rsrC*	Complementation strain of Δ*rsrC*	*Ku70*$^{-}$; *Hph*$^{R+}$; G418^{R+}; *Ble*$^{R+}$	This study
O*rsrC*	Overexpression strain of gene *rsrC*	*Ku70–; rsrC++; HphR+; G418R+*	This study
O*rsrA*Δ*rsrC*	Mutant where overexpression *rsrA* and *rsrC* are deleted	*Ku70*$^{-}$; *rsrC*$^{-}$; *rsrA*$^{++}$; *Hph*$^{R+}$; *G418*$^{R+}$; *Ble*$^{R+}$	This study
Δ*rsrA*Δ*rsrC*	Mutant where *rsrA* and *rsrC* are deleted	*Ku70*$^{-}$; *rsrC*$^{-}$; *rsrA*$^{-}$; *Hph*$^{R+}$; *G418*$^{R+}$; *Ble*$^{R+}$	This study

Supplementary References

1. Zhao, S., Yan, Y.S., He, Q.P., Yang, L., Yin, X., Li, C.X., Mao, L.C., Liao, L.S., Huang, J.Q., Xie, S.B., Nong, Q.D., Zhang, Z., Jing, L., Xiong, Y.R., Duan, C.J., Liu, J.L., & Feng, J.X. Comparative genomic, transcriptomic and secretomic profiling of *Penicillium oxalicum* HP7-1 and its cellulase and xylanase hyper-producing mutant EU2106, and identification of two novel regulatory genes of cellulase and xylanase gene expression. *Biotechnol Biofuels* **9**, 203 (2016).
2. Zhang, M. Y., Zhao, S., Ning, Y. N., Fu, L. H., Li, C. X., Wang, Q., You, R., Wang, C. Y., Xu, H. N., Luo, X. M., & Feng, J. X. Identification of an essential regulator controlling the production of raw-starch-digesting glucoamylase in *Penicillium oxalicum*. *Biotechnol. Biofuels* **12**, 7 (2019).
3. Ning, Y. N., Tian, D., Tan, M. L., Luo, X. M., Zhao, S., & Feng, J. X. Regulation of fungal raw-starch-degrading enzyme production depends on transcription factor phosphorylation and recruitment of the Mediator complex. *Commun. Biol*. **6**, 1032 (2023).

Supplementary Data

A RsrC-RsrA-RsrB transcriptional circuit positively regulates polysaccharide-degrading enzyme biosynthesis and development in *Penicillium oxalicum*

Yuan-Ni Ning[1,2,3#], Xue Liang[1,2,3#], Xin Shen[1,2,3], Di Tian[1,2,3], Wen-Tong Li[1,2,3], Xue-Mei Luo[3], Jia-Xun Feng[1,2,3*], Shuai Zhao[1,2,3*]

[1]State Key Laboratory for Conservation and Utilization of Subtropical Agro-bioresources, Guangxi University, 100 Daxue Road, Nanning, Guangxi 530004, People's Republic of China.

[2]Guangxi Research Center for Microbial and Enzyme Engineering Technology, Guangxi University, 100 Daxue Road, Nanning, Guangxi 530004, People's Republic of China.

[3]College of Life Science and Technology, Guangxi University, 100 Daxue Road, Nanning, Guangxi 530004, People's Republic of China.

Content:

Supplementary Fig. S16 Uncropped and unedited blots/gels corresponding to Supplementary Figures 3, 4, S1, S3, S6, S9, S11, S12

[#] These authors contributed equally to this work.

***Correspondence:** shuaizhao0227@gxu.edu.cn; jiaxunfeng@sohu.com

Tel: +86-771-323-9401

Mailing address: College of Life Science and Technology, Guangxi University, 100 Daxue Road, Nanning, Guangxi 530004, People's Republic of China

Fig. 3

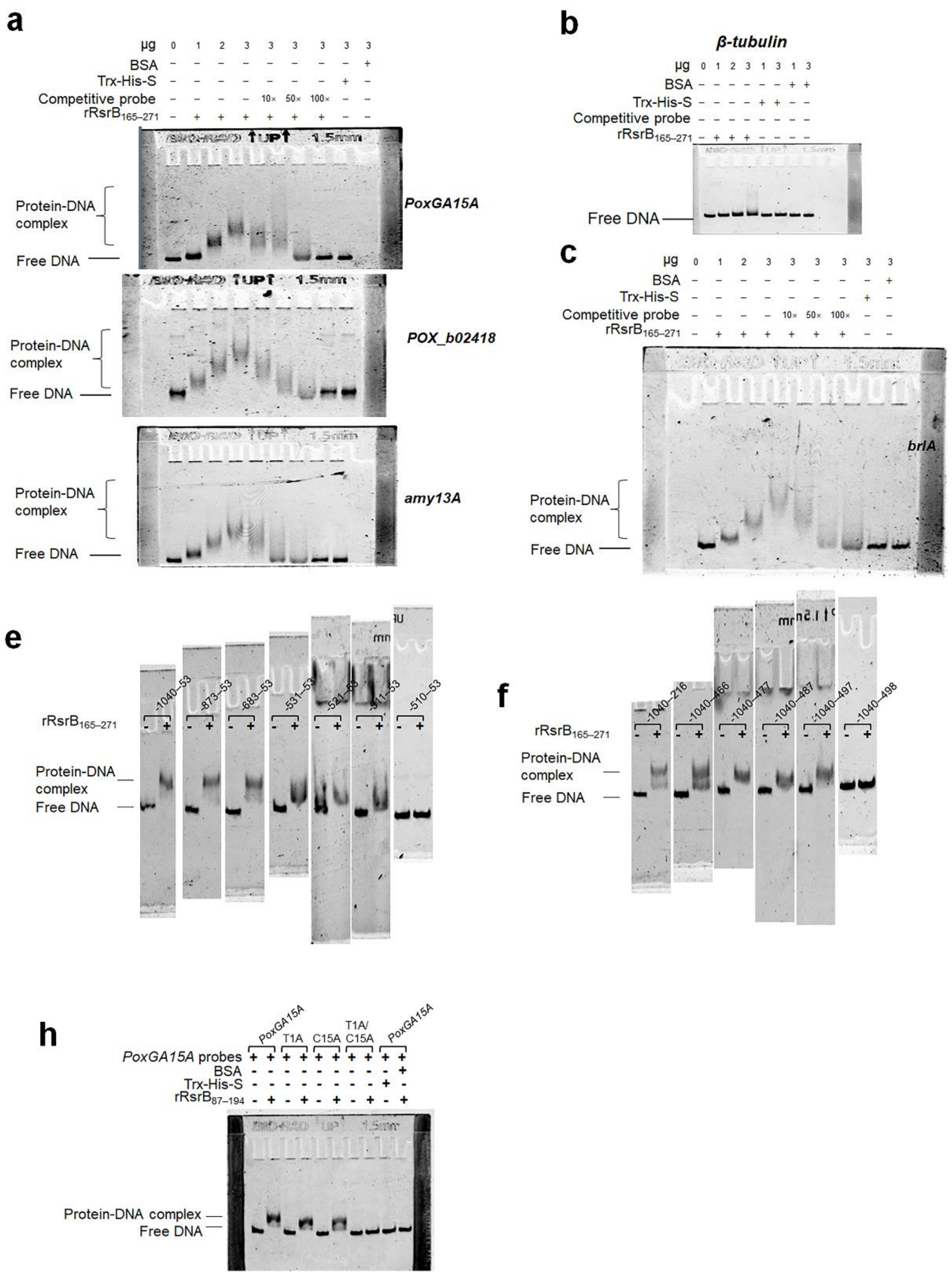

a
µg 0 1 2 3 3 3 3 3 3
BSA – – – – – – – – +
Trx-His-S – – – – – – – + –
Competitive probe – – – – 10× 50× 100× – –
rRsrB165–271 – + + + + + + – –
Protein-DNA complex
Free DNA
PoxGA15A
POX_b02418
amy13A
b
β-tubulin
µg 0 1 2 3 1 3 1 3
BSA – – – – – – + +
Trx-His-S – – – – + + – –
Competitive probe – – – – – – – –
rRsrB165–271 – + + + – – – –
Free DNA
c
µg 0 1 2 3 3 3 3 3 3
BSA – – – – – – – – +
Trx-His-S – – – – – – – + –
Competitive probe – – – – 10× 50× 100× – –
rRsrB165–271 – + + + + + + – –
brlA
Protein-DNA complex
Free DNA
e
rRsrB165–271
-1040–53
-873–53
-683–53
-531–53
-521–53
-511–53
-510–53
Protein-DNA complex
Free DNA
f
rRsrB165–271
-1040–216
-1040–466
-1040–477
-1040–487
-1040–497
-1040–498
Protein-DNA complex
Free DNA
h
PoxGA15A
T1A
C15A
T1A/ C15A
PoxGA15A
PoxGA15A probes + + + + + + + + + +
BSA – – – – – – – – – +
Trx-His-S – – – – – – – – + –
rRsrB87–194 – + – + – + – + – +
Protein-DNA complex
Free DNA

Fig. 4

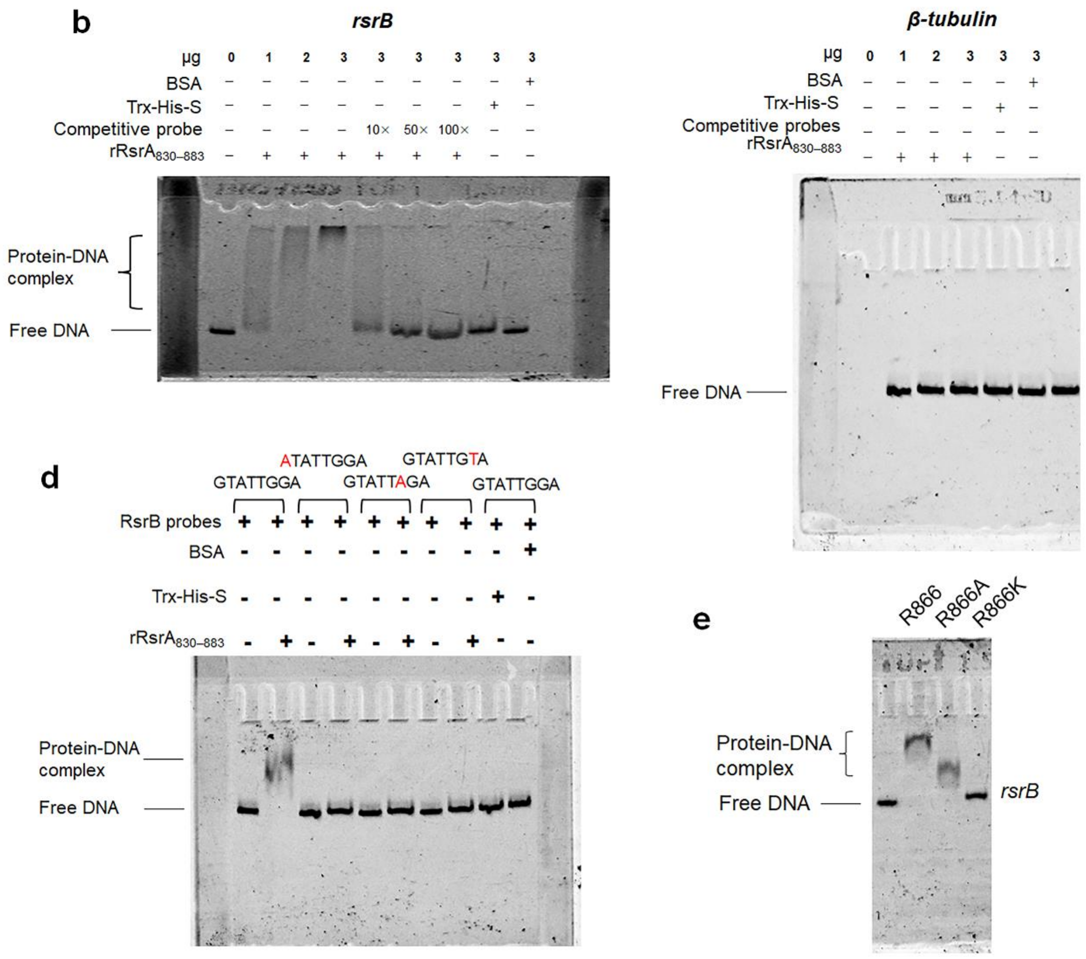
b
rsrB
μg 0 1 2 3 3 3 3 3 3
BSA – – – – – – – – +
Trx-His-S – – – – – – – + –
Competitive probe – – – – 10× 50× 100× – –
rRsrA830–883 – + + + + + + – –
Protein-DNA complex
Free DNA
β-tubulin
μg 0 1 2 3 3 3
BSA – – – – – +
Trx-His-S – – – – + –
Competitive probes – – – – – –
rRsrA830–883 – + + + – –
Free DNA
d
GTATTGGA
ATATTGGA
GTATTAGA
GTATTGTA
GTATTGGA
RsrB probes + + + + + + + + + +
BSA - - - - - - - - - +
Trx-His-S - - - - - - - - + -
rRsrA830–883 - + - + - + - + - -
Protein-DNA complex
Free DNA
e
R866
R866A
R866K
Protein-DNA complex
Free DNA
rsrB

Supplementary Fig. S1

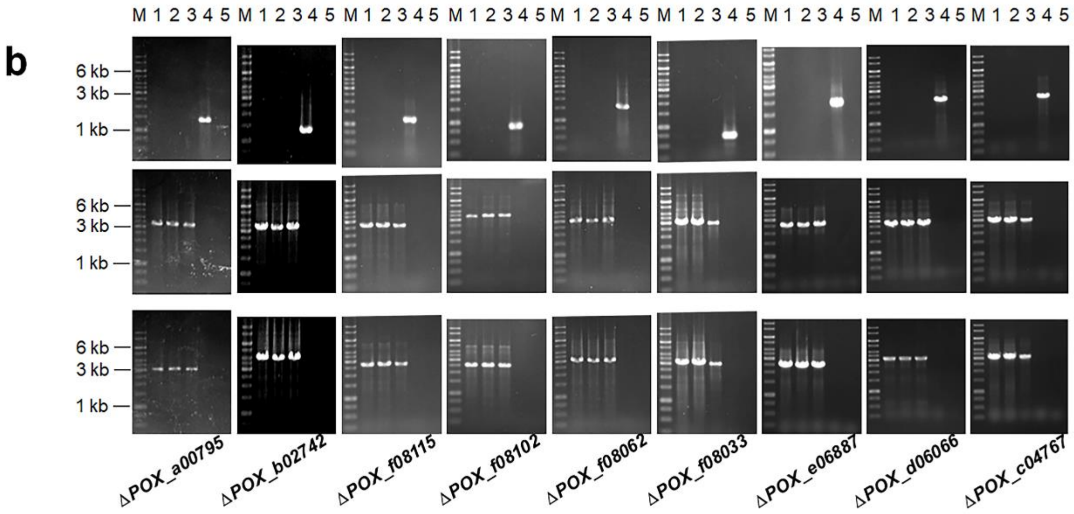

Supplementary Fig. S3

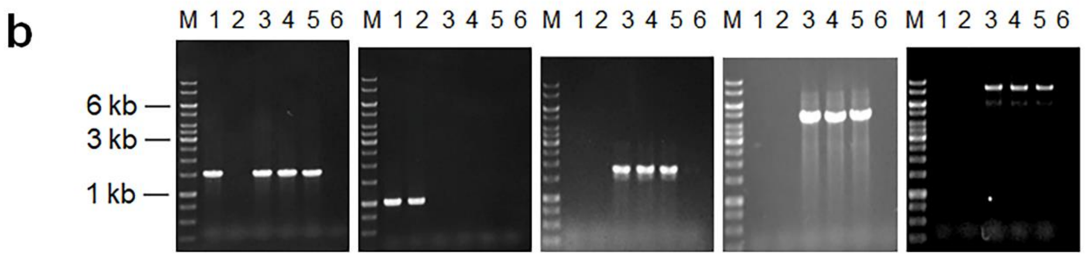

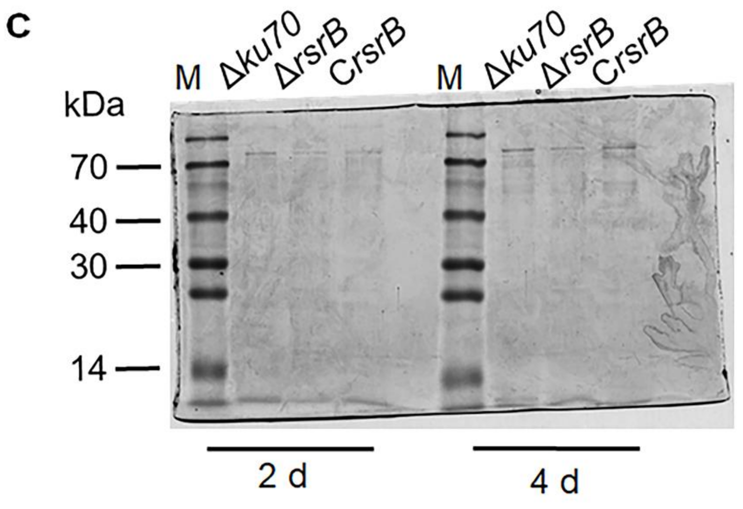

Supplementary Fig. S6

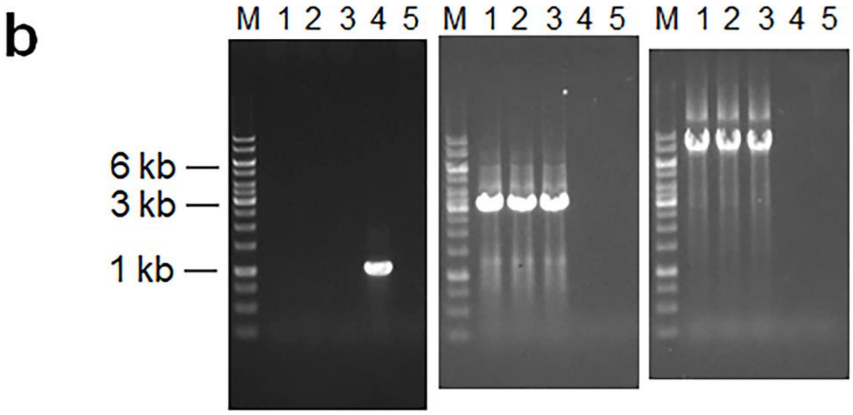

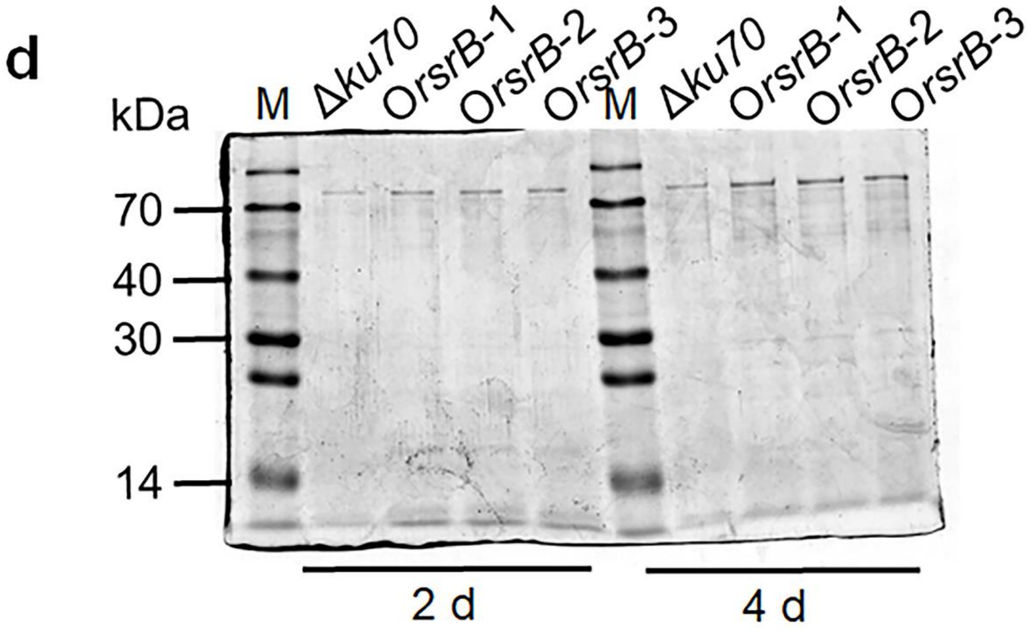

Supplementary Fig. S9

b

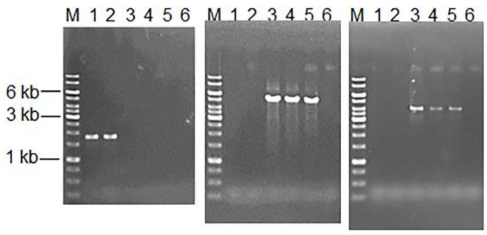

c

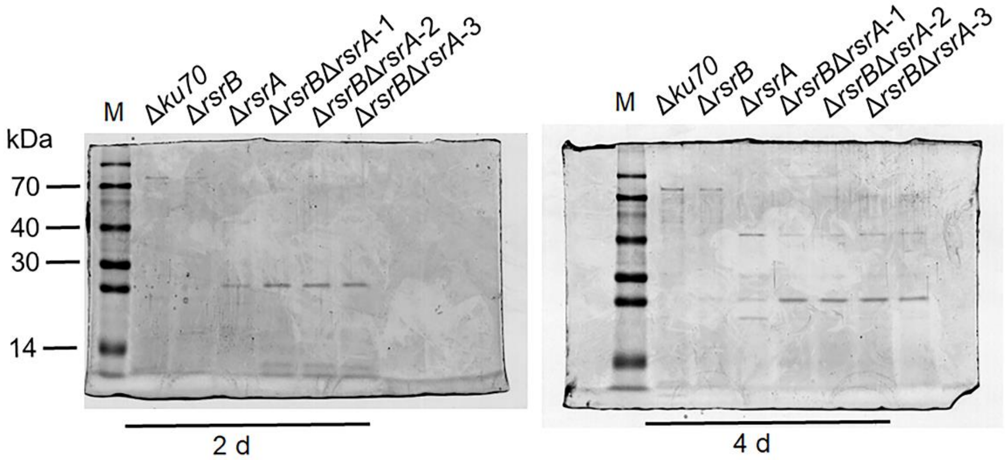

Supplementary Fig. S11

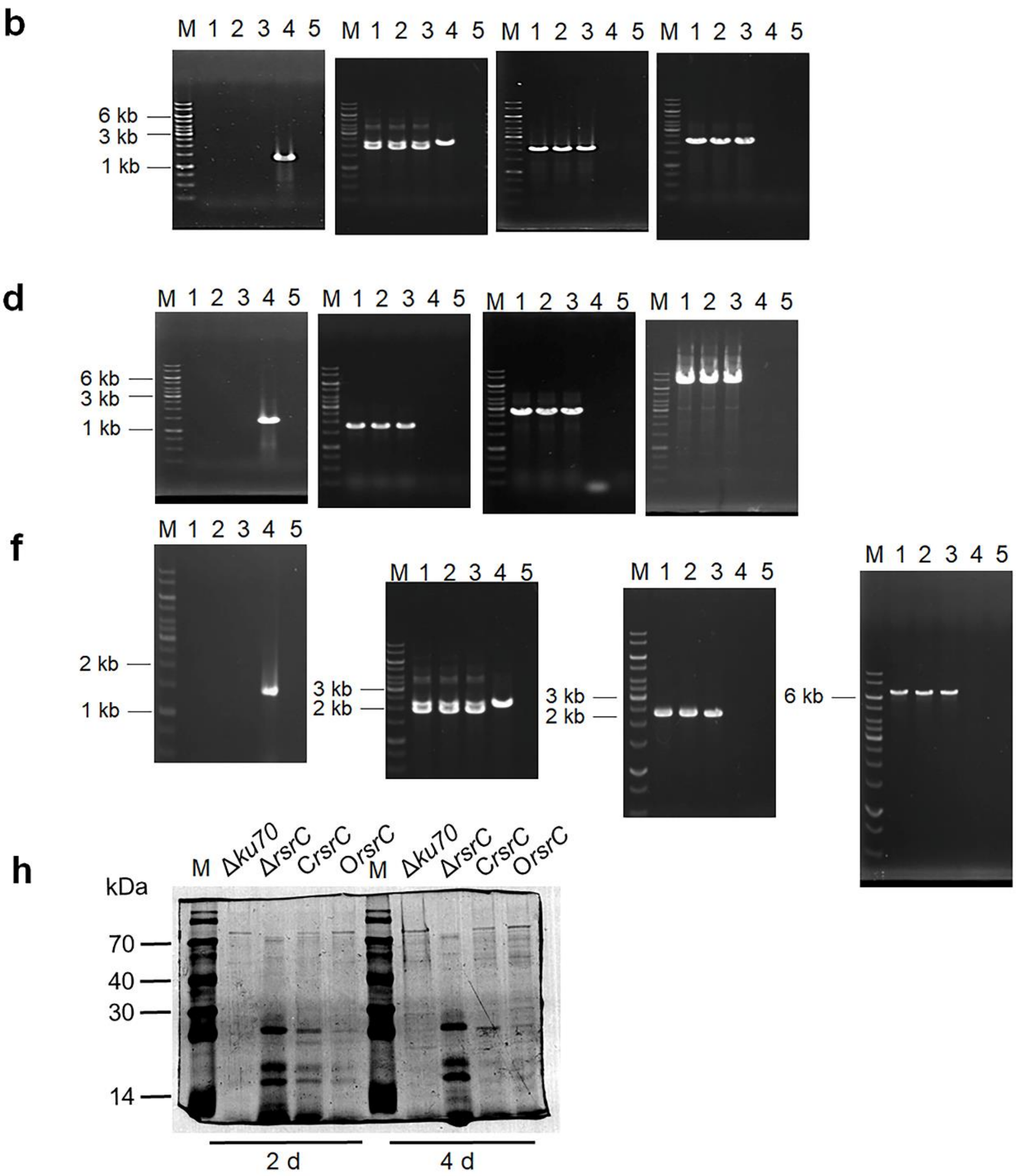

Supplementary Fig. S12

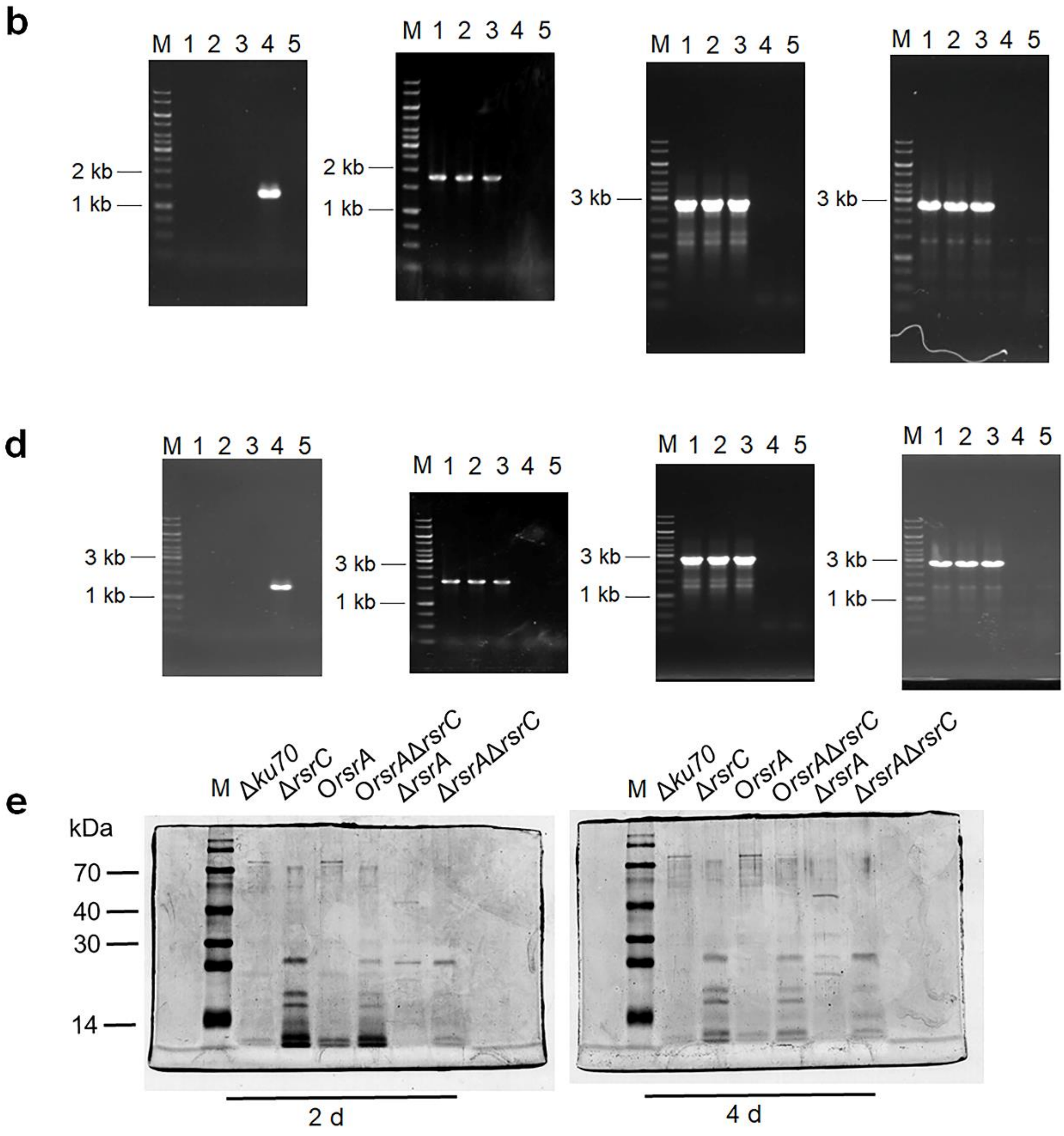

Supplementary Data

A RsrC-RsrA-RsrB transcriptional circuit positively regulates polysaccharide-degrading enzyme biosynthesis and development in *Penicillium oxalicum*

Yuan-Ni Ning[1,2,3#], Xue Liang[1,2,3#], Xin Shen[1,2,3], Di Tian[1,2,3], Wen-Tong Li[1,2,3], Xue-Mei Luo[3], Jia-Xun Feng[1,2,3*], Shuai Zhao[1,2,3*]

[1]State Key Laboratory for Conservation and Utilization of Subtropical Agro-bioresources, Guangxi University, 100 Daxue Road, Nanning, Guangxi 530004, People’s Republic of China.

[2]Guangxi Research Center for Microbial and Enzyme Engineering Technology, Guangxi University, 100 Daxue Road, Nanning, Guangxi 530004, People’s Republic of China.

[3]College of Life Science and Technology, Guangxi University, 100 Daxue Road, Nanning, Guangxi 530004, People’s Republic of China.

Content:

Supplementary Table S2 Primers used in this study

[#] These authors contributed equally to this work.

***Correspondence:** shuaizhao0227@gxu.edu.cn; jiaxunfeng@sohu.com

Tel: +86-771-323-9401

Mailing address: College of Life Science and Technology, Guangxi University,

100 Daxue Road, Nanning, Guangxi 530004, People's Republic of China

Supplementary Table S2 Primers used in this study

Name	Sequence (5'-3')
**Primers used for construction of deletion mutants of genes acting downstream and upstream of RsrA**	
POX_a00795-LF	GTGACCCACTCGACGTAAT
POX_a00795-LR	GGTAATCCTTCTTTCTAGATTTAGAGAATCTTTGTATCCCTTTA
POX_a00795-RF	AATATCATCTTCTGTCGACTGAAGATTATCCCGCATACAAC
POX_a00795 -RR	TTTAGGCTTGTTCACCAGTTATT
POX_a00795 -NF	GACCTATCCTCGGCGACCCT
POX_a00795 -NR	AATGAGTCCCTGGACCTCC
POX_a00795 -VF	ATGATGTTGACCGAAAGCG
POX_a00795 -VR	CCTTCACCAGCTCAAACCC
POX_b02742- LF	TCCAAGCACCCAACCCA
POX_b02742 -LR	GGTAATCCTTCTTTCTAGAGATGAACAGAGAAGTGGGAACAA
POX_b02742- RF	AATATCATCTTCTGTCGACATTGAAGCCTGTAAGGCCTCAA
POX_b02742 -RR	AGGCGGTCGTCAAGTCCA
POX_b02742 -NF	CACATCTGTCAACCTGCCTTAC
POX_b02742- NR	TTGGCGGTAACAGTGGTG
POX_b02742 -VF	CCGTATCATGGTATGTGCC
POX_b02742 -VR	GCGAGATGGACGAGGAG
POX_f08115- LF	TCTTGCCTGGCTAATGCG
POX_f08115 -LR	GGTAATCCTTCTTTCTAGAGACAGTCGGACAGTCCGTTG
POX_f08115 -RF	AATATCATCTTCTGTCGACTTATTTATGATAGATCTAATACTTTTTGA
POX_f08115- RR	CAATGCTCCCGCTCAAGT
POX_f08115- NF	GTGGTACGCCTGATGTTCC
POX_f08115- NR	AAGCAGTGCCGTGATGTTA
POX_f08115 -VF	CTTCCCGTGGCTGCTGTT
POX_f08115 -VR	TGGAGCCTGAGGGGTTGG
POX_f08102-LF	GCCGTCACCTTCCCATTA

POX_f08102-LR	GGTAATCCTTCTTTCTAGAGTCGTTGTCTTCGTCCAACAC
POX_f08102-RF	AATATCATCTTCTGTCGACAAACATTCCCAGCATAAGAAGT
POX_f08102-RR	CCGCCTTCTCAGAGTATTG
POX_f08102 - NF	GGTGTGATTCGCTCATTCTAA
POX_f08102-NR	GAAGGTCTAAATGGCACGCTA
POX_f08102-VF	GTCCAGCCCGCTCATAGT
POX_f08102-VR	TGTGGTGCGATTCGTTAG
POX_f08062- LF	CGACAGTACACTGCGGACTGA
POX_f08062- LR	GGTAATCCTTCTTTCTAGACTTGGGGGGTGTAGGTAGTGA
POX_f08062 -RF	AATATCATCTTCTGTCGACGAGCTCCTTGCGCATGA
POX_f08062- RR	GGGTCCGCACTTACATAGA
POX_f08062- NF	AACAGCCGCAGAATCCAAA
POX_f08062 -NR	GGCTGCTGCGTAAGCGATA
POX_f08062 -VF	CATTACGGCAACAAGACCC
POX_f08062 -VR	TTGCGAGATGAGTGGAGAAGAG
POX_f08033 -LF	CCCAGTAGAAGCCCATTAGAC
POX_f08033 -LR	GGTAATCCTTCTTTCTAGATTTGAACCAAGGGGGAGAA
POX_f08033- RF	AATATCATCTTCTGTCGACGAAATGATTTGGTATGACTGTATTG
POX_f08033 -RR	TGGGTCACTCCGTACAAGG
POX_f08033 -NF	ATTCTCGCCCCAACAGGAC
POX_f08033 -NR	TTCTCAGCCTTGAGTGTTTTACTA
POX_f08033 -VF	AGCGTTCTGTCCAGCAATT
POX_f08033 -VR	TACTCGACCCTCCTTCCAC
POX_e06887 -LF	ATCTCTGTGGCATCAAGGTATT
POX_e06887- LR	GGTAATCCTTCTTTCTAGAGATGGGTGATCTTAAACAGAACG
POX_e06887 -RF	AATATCATCTTCTGTCGACGAGAAACATTTCCAACTGCTGC
POX_e06887 -RR	TGCCGAGACACGCAGGATA
POX_e06887 -NF	GCAAAAGTGAGTGCTTCTACCA
POX_e06887- NR	GCCCAGTTGGTTGAAGAGGTT

POX_e06887 -VF	TCTCGCCCTGCCCTATC
POX_e06887 - VR	CGGGTGGTTCGTTCCTA
POX_d06066- LF	CGTGTACCATTTGACCGTGTC
POX_d06066 -LR	GGTAATCCTTCTTTCTAGAGATCTTGTGATCAATCAGATCGC
POX_d06066 -RF	AATATCATCTTCTGTCGACGTCCGCAATGTGGTGAGA
POX_d06066- RR	AACGCCAGTGATACCGATAG
POX_d06066 -NF	TGGGTCAGGTTCCTTCCTGG
POX_d06066 -NR	CTTGAACCAAACTATCAATAACCT
POX_d06066 -VF	CCCAAGATTCCACGACCT
POX_d06066 -VR	AACACTCCCGCACATACAC
POX_c04767 -LF	ATGCGTTCACGGAGTTGG
POX_c04767 -LR	GGTAATCCTTCTTTCTAGAATTCGCTGTCGGTGAATTAAA
POX_c04767 -RF	AATATCATCTTCTGTCGACGGCATTAAATAGCGTCCTGAA
POX_c04767 -RR	CATTGCCACGCCATCCG
POX_c04767- NF	GGTCTGTTTTACGGGGCTGAAT
POX_c04767 -NR	GCCGCAATATATGTGTCCGTCT
POX_c04767 -VF	GCATTTGCGATGGCTACA
POX_c04767 -VR	CATCAAGGACACACCACTACC
g418-F	TCTAGAAAGAAGGATTACC
g418-R	GTCGACAGAAGATGATATT
g418-VR	GTGAATGCTCCGTAACACCCAAT
g418-VF	CGCTACTGCTTACAAGTGGGCTGAT
**Primers for construction of complementation C*rsrB* strains**	
POX_d05452-LF	GGCACCAAACACCGCTACA
POX_d05452-LR	TTTAGAGGTAATCCTTCTTTCTAGAGATGGACGAGACGGTACGATT
POX_d05452-RF	ATGTAGATCACCAAGTTGCA
POX_d05452-RR	TTCGGACGGTGTCACTTCG
POX_d05452-NF	AATGAGCGTAGTGTGAGAGCACCAAT
POX_d05452-NR	GTCGCACATCCAATCGCATAGAGT

POX_d05452-VF	CGCCTACGGAAAATGACG
POX_d05452-VR	ATGTCGCCGAAGATGGAG
*POX_g08691*P-F	TCTTACCGCTGTTGAGATCCAGAACATCTATATCGCGCCAA
*POX_g08691*Ter-R	TGCAACTTGGTGATCTACATTGATTGAAGTTGTAGGCAGGC
ble-F	TCTAGAAAGAAGGATTACCTC
ble-R	CTGGATCTCAACAGCGGTA
**Primers used for Yeast Y2HGold autoactivation experiment**	
rsrB-F	CATGGAGGCCGAATTCATGCCAGGGTTCCTCTTCAGAC
rsrB-R	GCAGGTCGACGGATCCTTATACCCAAAAGTCTAGCG
$rsrB_{176}$-F	CATGGAGGCCGAATTCATGAAAGTCAATGCGCCAGCCTGC
$rsrB_{210}$-R	GCAGGTCGACGGATCCTTACTGACATTCTAGATCCCGTGT
$rsrB_{211}$-F	CATGGAGGCCGAATTCATGTATATTCTCGAGAAACCAGCA
rsrC-F	CATGGAGGCCGAATTCATGGCGCCAGGAAACG
rsrC-R	GCAGGTCGACGGATCCTTACTGACCCTCCTGTATCTGG
rsrC-U1-F	CATGGAGGCCGAATTCATGGCGCCAGGAAACG
rsrC-U1-R	GCAGGTCGACGGATCCTTACTGCACATACTGCTGCTGC
rsrC-U2-F	CATGGAGGCCGAATTCATGGCGCCAGGAAACG
rsrC-U2-R	GCAGGTCGACGGATCCTTACGTGCCCATCGGG
rsrC-U3-F	CATGGAGGCCGAATTCATGGCGCCAGGAAACG
rsrC-U3-R	GCAGGTCGACGGATCCTTATGCATCCTCCGACGAT
rsrC-U4-F	CATGGAGGCCGAATTCATGCCTCATGTGTGCGACCA
rsrC-U4-R	GCAGGTCGACGGATCCTTAACCTACTCCCAAAAACCC
rsrC-U5-F	CATGGAGGCCGAATTCATGTCGGAGGATGCAGGGT
rsrC-U5-R	GCAGGTCGACGGATCCTTAACCTACTCCCAAAAACCC
rsrC-U6-F	CATGGAGGCCGAATTCATGCCGATGGGCACG
rsrC-U6-R	GCAGGTCGACGGATCCTTAACCTACTCCCAAAAACCC
rsrC-U7-F	CATGGAGGCCGAATTCATGCCGCAGCATCAGCA
rsrC-U7-R	GCAGGTCGACGGATCCTTAACCTACTCCCAAAAACCC
**Primers used for heterologous expression of rsrB**	

rsrB-F	CATGGCTGATATCGGATCCGAATTCATGGATGGGTGTAATTCGGG
rsrB-R	GAGTGCGGCCGCAAGCTTGTCGACGTTAGACAGGTGTTCCTGAGATTGTATCA
**Primers used for amplification of EMSA probes**	
PoxGA15A-F	ATGAAGGATCTCCAAGTGTAGC
PoxGA15A-R	FAM-AGTGATGAGCCTGGTAGAAGAA
POX_b02418-F	TGGGTCGCCTCAACATCTA
POX_b02418-R	FAM-CTTGACCGAGCGAAAATCAGAA
POX_g08691-F	GCTTATCTATCGCCGTGCT
POX_g08691-R	FAM-TGACTTTGCGTGTGTCATCG
PoxAmy13A-F	TCGGACCAACCCATAAGG
PoxAmy13A-R	FAM-TGCCTCCTGATGATACCACA
brlA-F	GGAACTATAAACCCGCCCT
brlA-R	FAM-CATGTCGTCGAGTTCTTCAATT
arpB-F	TCAAGTCTGGCAATGAAGCG
arpB-R	FAM-TATGTATTAGGGATGATGATGTAGT
β-tubulin-F	ACCTCACTTGCTCCGCTCTG
β-tubulin-R	FAM-ACAAACTTCATAGATGGAGTGGACA
$rsrB_{G1A}$-F	GCGCTTATATTGGAGATTGG
$rsrB_{G1A}$-R	CCAATCTCCAATATAAGCGC
$rsrB_{G6A}$-F	GCGCTTGTATTAGAGATTGG
$rsrB_{G6A}$-R	CCAATCTCTAATACAAGCGC
$rsrB_{G7T}$-F	GCGCTTGTATTGTAGATTGG
$rsrB_{G7T}$-R	CCAATCTACAATACAAGCGC
PoxGA15A-F1	ATGAAGGATCTCCAAGTGTAGC
PoxGA15A-F2	FAM -ATGAAGGATCTCCAAGTGTAGC
PoxGA15A-R1	AGTGATGAGCCTGGTAGAAGAA
PoxGA15A-R2	FAM-AGTGATGAGCCTGGTAGAAGAA

PoxGA15A$_{-873}$-F	TTTGGGCTTGCCTATGTCCG
PoxGA15A$_{-683}$-F	ACGGGCAAAAGACGGAGATT
PoxGA15A$_{-531}$-F	GAGTCTTGGAGGGGGCGAAT
PoxGA15A$_{-521}$-F	GGGGGCGAATTCGATCAGGCAC
PoxGA15A$_{-511}$-F	TCGATCAGGCACGCCCGTCCAT
PoxGA15A$_{-510}$-F	CGATCAGGCACGCCCGTCCAT
PoxGA15A$_{-216}$-R	GTTTTCTACTGGGGGGTTCC
PoxGA15A$_{-466}$-R	AAGAACCTAGCCGATAGTGTGA
PoxGA15A$_{-477}$-R	CGATAGTGTGAAGATGGACG
PoxGA15A$_{-487}$-R	AAGATGGACGGGCGTGCCTGAT
PoxGA15A$_{-497}$-R	GGCGTGCCTGATCGAATTCGCC
PoxGA15A$_{-498}$-R	GCGTGCCTGATCGAATTCGCC
PoxGA15A$_{T1A}$-F	CGAATACGATCAGGCACGCCCGTCCATCTTCA
PoxGA15A$_{T1A}$-R	TGAAGATGGACGGGCGTGCCTGATCGTATTCG
PoxGA15A$_{C15A}$-F	CGAATTCGATCAGGCACGCACGTCCATCTTCA
PoxGA15A$_{C15A}$-R	TGAAGATGGACGTGCGTGCCTGATCGAATTCG
PoxGA15A$_{T1A/C15A}$-F	CGAATACGATCAGGCACGCACGTCCATCTTCA
PoxGA15A$_{T1A/C15A}$-R	TGAAGATGGACGTGCGTGCCTGATCGTATTCG
**Primers used for RT-qPCR assay**	
POX_g08691-F	TGGCACTTCACCTTGGACTTAC
POX_g08691-R	AGTGGCACATCCTGGTCAACAA
PoxGA15A-F	CCTCGGTGAGCCCAAGTT
PoxGA15A-R	CCAAAGTCAATCAAGGCAA
POX_b02418-F	TATGTGGATTCCTTCCGCTCTA
POX_b02418-R	ATGGATTGCCTCCTTGGT
POX_g08691-F	TGGCACTTCACCTTGGACTTAC
POX_g08691-R	AGTGGCACATCCTGGTCAACAA

PoxAmy13A-F	CTGACGGCTGCCCAATG
PoxAmy13A-R	CCAAATCGCAGTAAATCCC
brlA-F	CCAGTTGCCTGTTTCGTCAG
brlA-R	GGTAAGGGAATGTCGGGTGTT
arpB-F	AGAGTCTCGGCGTTCAAGC
arpB-R	TGGTCGTAGGCGTTCTTGG
Actin-F	CTCCATCCAGGCCGTTCTG
Actin-R	CATGAGGTAGTCGGTCAAGTCAC
rsrA-F	GAGCTCGGTACCCTCTTCTC
rsrA-R	GCAGGTCGACGGATCCTTAC
rsrC-F	GCACTGTGGCAAGTCTTTCAA
rsrC-R	TTCTGGCAGCCTTCGTGGT
**Primers used for construction of Δ*rsrB*Δ*rsrA* strains**	
rsrA-LF	CGTCAAACTCCTGTGCCGTAT
rsrA-LR	TTTAGAGGTAATCCTTCTTTCTAGACCGGGCCACCCGATGAAGCG
rsrA-RF	TCTTACCGCTGTTGAGATCCAGGTTGGACGTGCGACATGAG
rsrA-RR	CCAAGTCCATTCACCCTGTA
rsrA-NF	GGTTCAATGGGTCCTGCTTC
rsrA-NR	TGAAGCGGACGCCTCATAAC
rsrA-VF	GTTCCTGCCTTCGGTTCA
rsrA-VR	TCGCATCACTCGGGTCAA
ble-F	TCTAGAAAGAAGGATTACCTC
ble-R	CTGGATCTCAACAGCGGTA
**Primers used for construction of O*rsrB* strains**	
POX_d05452-LF	GGCACCAAACACCGCTACA
POX_d05452-LR	GGTAATCCTTCTTTCTAGAGATGGACGAGACGGTACGATT
POX_d05452-RF	AACCTTCTTCCTCTCATCGTCCATGTAGATCACCAAGTTGCA
POX_d05452-RR	TTCGGACGGTGTCACTTCG
g418-F	TCTAGAAAGAAGGATTACC

g418-R	GTCGACAGAAGATGATATT
*rsrB*P-F	AATATCATCTTCTGTCGACAACATCTATATCGCGCCAA
*rsrB*Ter-R	GGACGATGAGAGGAAGAAGGTT
POX_d05452-NF	AATGAGCGTAGTGTGAGAGCACCAAT
POX_d05452-NR	GTCGCACATCCAATCGCATAGAGT
POX_d05452-VF	CGCCTACGGAAAATGACG
POX_d05452-VR	ATGTCGCCGAAGATGGAG
**Primers used for construction of Δ*rsrC* strains**	
rsrC-LF	ATCGGGTCGCCGCCTC
rsrC+ble-R	CACAGTGATCGGCGAGTTG
rsrC-RF	AAGGGAATCCCGCCTAGAT
rsrC-RR	AATCGGCTGGTGGCATG
rsrC-F	ATGGCGCCAGGAAACG
rsrC-R	TTAACCTACTCCCAAAAACCC
g418-F	TCTAGAAAGAAGGATTACCTCTAAA
g418-R	GTCGACAGAAGATGATATTGAAG
g418-VF	CTAAATGAACCATCTTGTCAAACGA
g418-VR	AGCCCTGGGTTCGCAAAG
**Primers used for construction of C*rsrC* strains**	
POX_d05452-LF	GAGCGTAGTGTGAGAGCACCAAT
ble-R	TCAAAAGCCCTGAGGTGAAACTGGATCTCAACAGCGGTAAG
*rsrC*p-F	TTTCACCTCAGGGCTTTTG
*rsrC*Ter-R	CTCCCCCGAACATACCG
POX_d05452-RF	ATCCGGTATGTTCGGGGGAGATGTAGATCACCAAGTTGCACGC
POX_d05452-RR	CCACCGTCGCAATCGTCA
POX_d05452-F	ATGGTTGTCTTCAGCAAGGTTA
POX_d05452-R	CTATGCCTGAGCAGCGAAAC
ble-VF	ATAGCCGCTGCTGGTTTC
ble-VR	AATAAATCCTGGTGTCCCTGTT

Primers used for construction of O*rsrC* strains

*POX_d05452*L+*g418*-F	GAGCGTAGTGTGAGAGCACCAAT
*POX_d05452*L+*g418*-R	GTCACCGCCAAACCACGAGCGTCGACAGAAGATGATATTGAAG
*POX06702*P-F	CAATATCATCTTCTGTCGACGCTCGTGGTTTGGCGG
*POX06702*P-R	CGACCGTTTCCTGGCGCCATGGTGACGGTTGATCAAGAATG
rsrC-F	ATTCTTGATCAACCGTCACCATGGCGCCAGGAAACG
rsrC-R	AGCTCCTCGCCCTTGCTCACACCTACTCCCAAAAACCCCCA
gfp-F	GGGGGTTTTTGGGAGTAGGTGTGAGCAAGGGCGAGGAG
gfp-R	CATCTAGGCGGGATTCCCTTTTACTTGTACAGCTCGTCCATG
*rsrC*Ter-F	TGGACGAGCTGTACAAGTAAAAGGGAATCCCGCCTAGAT
*rsrC*Ter-R	TGCAACTTGGTGATCTACATCTCCCCCGAACATACCG
POX_d05452R-F	ATCCGGTATGTTCGGGGGAGATGTAGATCACCAAGTTGCACG
POX_d05452-RR	GAAGAAAGTGCGAGGCGTGA
POX_d05452-F	ATGGTTGTCTTCAGCAAGGTTA
POX_d05452-R	CTATGCCTGAGCAGCGAAAC
POX_d05452-NF	CCCGTGACCAGGGTCCTTG
POX_d05452-NR	GAGGGTCCCCAATAACTCGG

Primers used for construction of Δ*rsrA*Δ*rsrC* strains

rsrC-LF	ATCGGGTCGCCGCCTC
rsrC-LR	AGGTAATCCTTCTTTCTAGACACAGTGATCGGCGAGTTGA
rsrC-RF	TTACCGCTGTTGAGATCCAGAAGGGAATCCCGCCTAGAT
rsrC-RR	AATCGGCTGGTGGCATG
ble-F	TCTAGAAAGAAGGATTACCTCTAAA
ble-R	CTGGATCTCAACAGCGGTA
ble-vF	ATAGCCGCTGCTGGTTTC
ble-vR	AATAAATCCTGGTGTCCCTGTT

Primers used for construction of O*rsrA*Δ*rsrC* strains

rsrC-LF	ATCGGGTCGCCGCCTC
rsrC-LR	AGGTAATCCTTCTTTCTAGACACAGTGATCGGCGAGTTGA

rsrC-RF	TTACCGCTGTTGAGATCCAGAAGGGAATCCCGCCTAGAT
rsrC-RR	AATCGGCTGGTGGCATG
ble-F	TCTAGAAAGAAGGATTACCTCTAAA
ble-R	CTGGATCTCAACAGCGGTA
ble-VF	ATAGCCGCTGCTGGTTTC
ble-VR	AATAAATCCTGGTGTCCCTGTT
**Primers used for construction of Y1H assay**	
*rsrA*R1-F	TGAATTCGAGCTCGGTACCCCTCCGTTCACAGCCTCACT
*rsrA*R1-R	AGCACATGCCTCGAGGTCGACCGGGCCACCCGATGA
*rsrA*R2-F	TGAATTCGAGCTCGGTACCCCTCCGTTCACAGCCTCACT
*rsrA*R3-F	TGAATTCGAGCTCGGTACCCAGATCCCATCCTCAAGACTCAGT
*rsrA*R4-F	TGAATTCGAGCTCGGTACCCCCCCTTCGTCCGGGTTTA
*rsrA*R5-F	TGAATTCGAGCTCGGTACCCGGTGGTGGTGGTGGAGGT
*rsrA*R6-F	TGAATTCGAGCTCGGTACCCCGTGGATCGCGACTACGGTGG
*rsrA*R7-F	TGAATTCGAGCTCGGTACCCCTCCGTTCACAGCCTCACT
*rsrA*R7-R	AGCACATGCCTCGAGGTCGATCCTCCTCCTCCACTGAAAC
*rsrA*R8-F	TGAATTCGAGCTCGGTACCCGTCGGTGGCGTGCACGGA
*rsrA*R9-F	TGAATTCGAGCTCGGTACCCACCCACAGAGAGGCTGTATATGC
*rsrA*R10-F	TGAATTCGAGCTCGGTACCCAGATCCCATCCTCAAGACTCAGT
*rsrA*R11-F	TGAATTCGAGCTCGGTACCCCTCCGTTCACAGCCTCACT
*rsrA*R11-R	AGCACATGCCTCGAGGTCGATGCACTTTCCTTCCAGTTG
*rsrA*R12-F	TGAATTCGAGCTCGGTACCCAATAAATGGTTAAATCGAGCC
*rsrA*R13-F	TGAATTCGAGCTCGGTACCCGTCGGTGGCGTGCACG
*rsrA*R13-R	AGCACATGCCTCGAGGTCGATGCACTTTCCTTCCAGTTGC
*rsrA*R14-F	TGAATTCGAGCTCGGTACCCACCCACAGAGAGGCTGTATATG
*rsrA*R15-F	TGAATTCGAGCTCGGTACCCCAATTAGGTCCTTCTTCATGTG
*rsrA*R16-F	TGAATTCGAGCTCGGTACCCTGGTCCGTGCAGAAGATCA
*rsrA*R17-F	TGAATTCGAGCTCGGTACCCTCTTCTCGTTGGCTCGGTT
