# Supplementary material for: A RsrC-RsrA-RsrB transcriptional circuit positively regulates polysaccharide-degrading enzyme biosynthesis and development in Penicillium oxalicum
Source: Commun Biol. 2024 Jul 11;7:848. doi: 10.1038/s42003-024-06536-4 (PMC11239660; doi:10.1038/s42003-024-06536-4)
Supplement: Supplementary file 2 — Description of Additional Supplementary Materials [file 42003_2024_6536_MOESM2_ESM.pdf]

Description of Additional Supplementary Files

File name: Supplementary Data 1

Description: List of 4041 differentially expressed genes in the Δ*rsrB* as compared with the parental strain Δ*ku70* grown on SCS

File name: Supplementary Data 2

Description: List of 4137 differentially expressed genes in the Δ*rsrC* as compared with the parental strain Δ*ku70* grown on SCS

File name: Supplementary Data 3

Description: List of 5656 differentially expressed genes in the Δ*rsrA* as compared with the parental strain Δ*ku70* grown on SCS

File name: Supplementary Data 4

Description: The source data behind the graphs in the paper
